# Supplementary material for: Autoimmune alleles at the major histocompatibility locus modify melanoma susceptibility
Source: Am J Hum Genet. 2023 Jun 19;110(7):1138–61. doi: 10.1016/j.ajhg.2023.05.013 (PMC10357503; doi:10.1016/j.ajhg.2023.05.013)
Supplement: Document S1. Figures S1–S17 and Tables S1–S3 [file mmc1.pdf]

The American Journal of Human Genetics, Volume *110*

Supplemental information

Autoimmune alleles at the major histocompatibility locus modify melanoma susceptibility

James V. Talwar, David Laub, Meghana S. Pagadala, Andrea Castro, McKenna Lewis, Georg E. Luebeck, Bryan R. Gorman, Cuiping Pan, Frederick N. Dong, Kyriacos Markianos, Craig C. Teerlink, Julie Lynch, Richard Hauger, Saiju Pyarajan, Philip S. Tsao, Gerald P. Morris, Rany M. Salem, Wesley K. Thompson, Kit Curtius, Maurizio Zanetti, and Hannah Carter

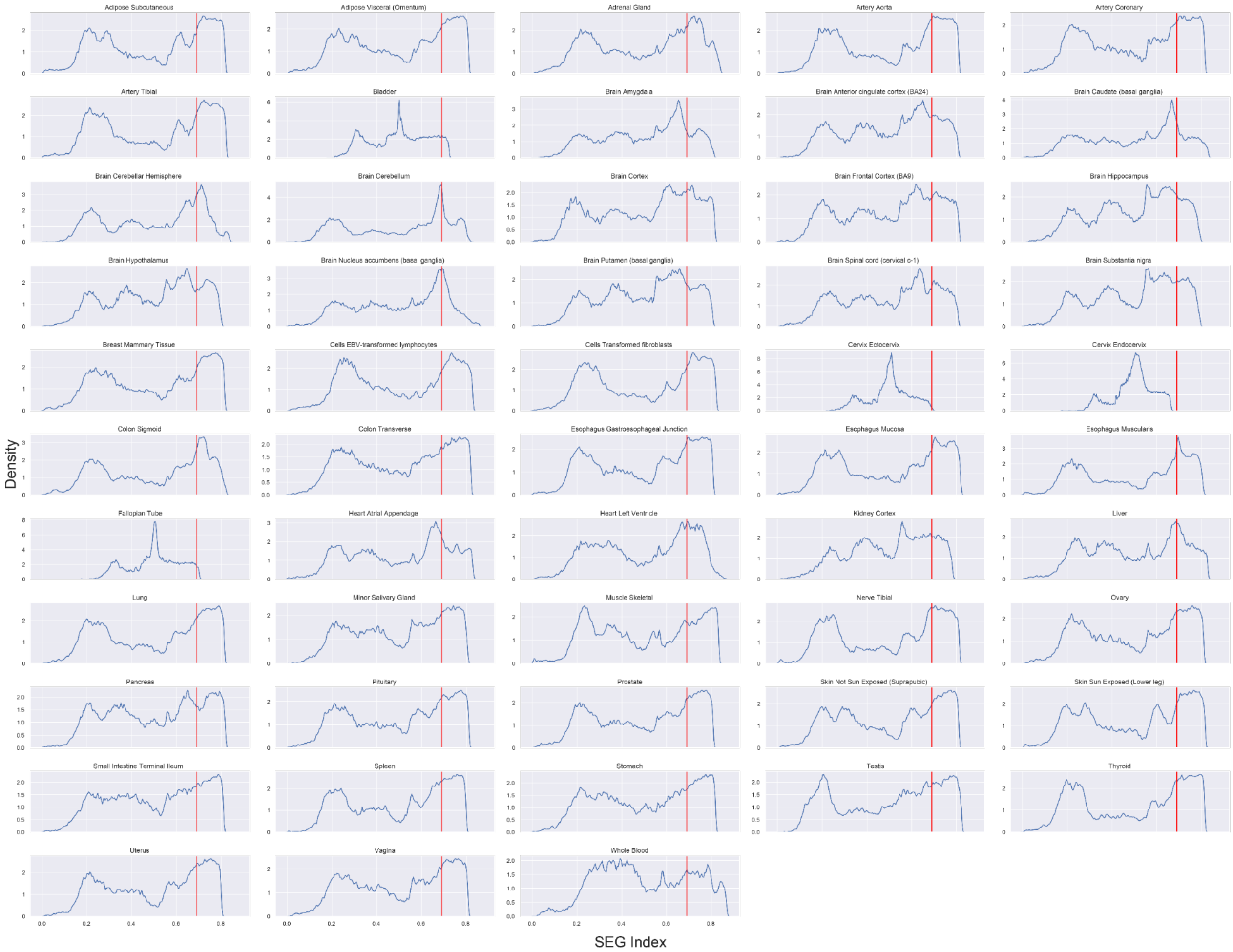

Figure S1: Distributions of stably expressed gene (SEG) scores shown for all 53 tissues in GTEx. The threshold of 0.69 was selected and is shown in red.

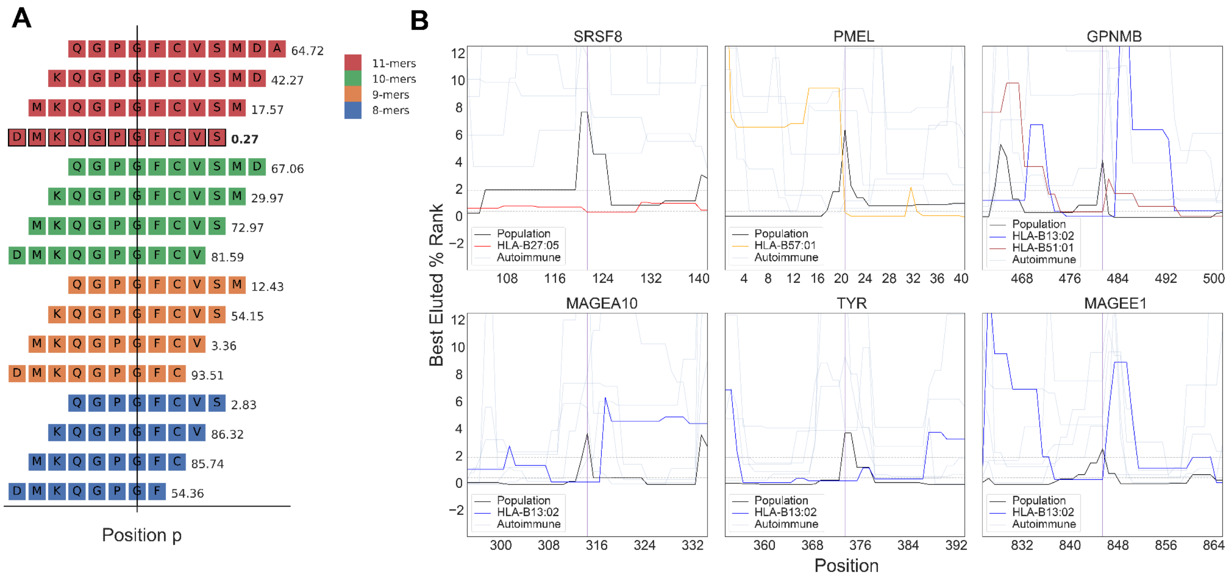

Figure S2: A) Position-wise best percentile rank scores were computed from predictions for 8-11mers by taking the best percentile rank of all overlapping peptides at any given position. B) Greatest position-wise differences in best percentile rank between HLA-B autoimmune and common alleles amongst the top 2 differences (*SRSF8* and the canonical melanocyte gene *PMEL*) and several DE conserved antigens (*GPNMB, MAGEA10, TYR, MAGEE1*). Common alleles are shown in black and autoimmune alleles are shown in transparent blue unless they are predicted to elute at better percentile ranks than common alleles. Vertical purple lines demarcate positions where autoimmune alleles are predicted to elute at better percentile ranks than common alleles. Plots show up to +/- 20 amino acids of the demarcated positions.

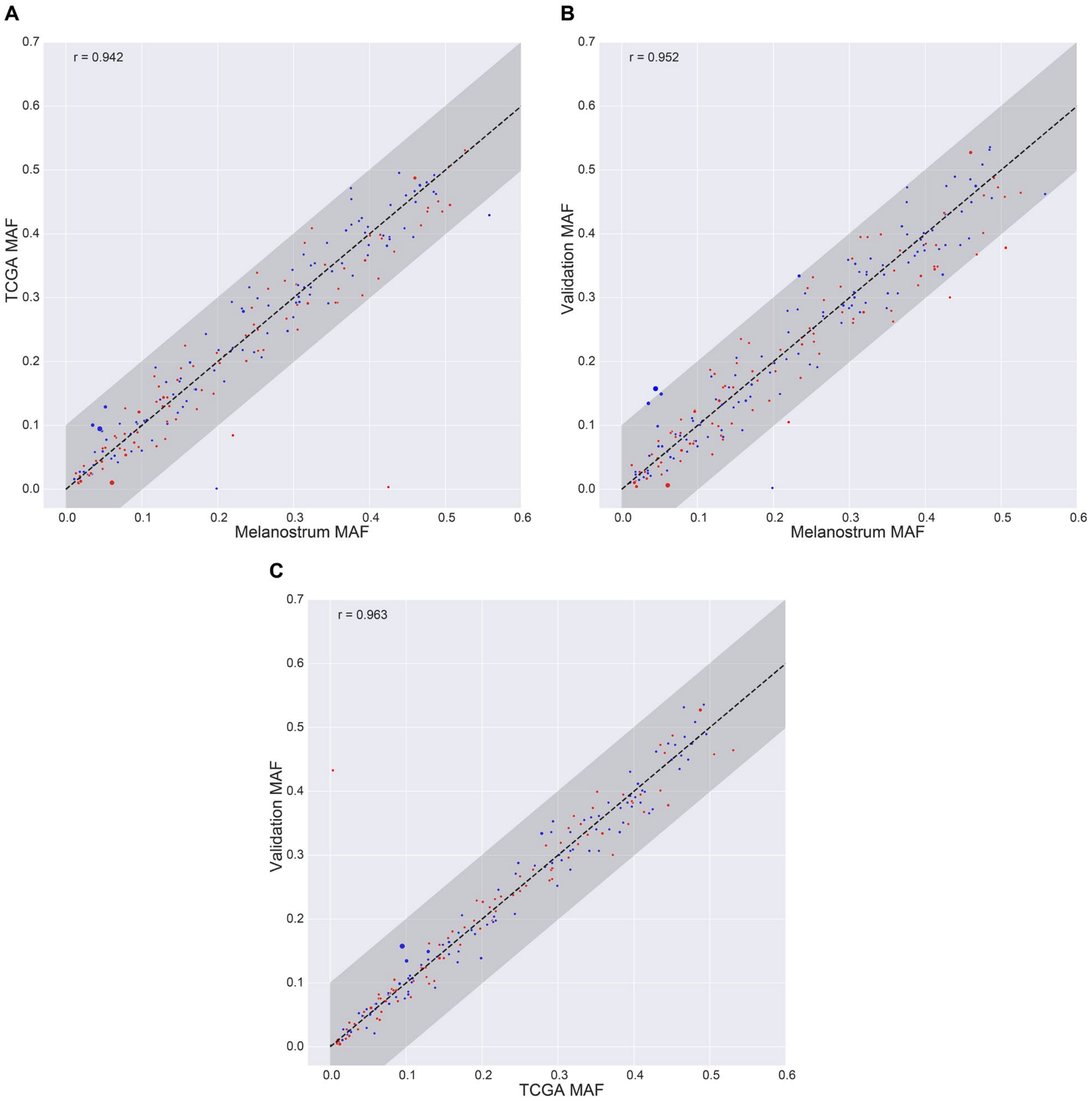

Figure S3: PRS SNP minor allele frequencies (MAFs) relationship between A) Discovery and Melanostrum (Pearson R = 0.942), B) Validation and Melanostrum (Pearson R = 0.952), and C) Validation and Discovery (Pearson R = 0.963). Red points are PRS SNPs with a protective effect in melanoma (negative PRS weight), while blue points are PRS SNPs with a predisposing melanoma effect (positive PRS weight). Points are sized according to the magnitude of their weight in the PRS. SNP effects ranged in PRS weight from -0.4 to 0.49.

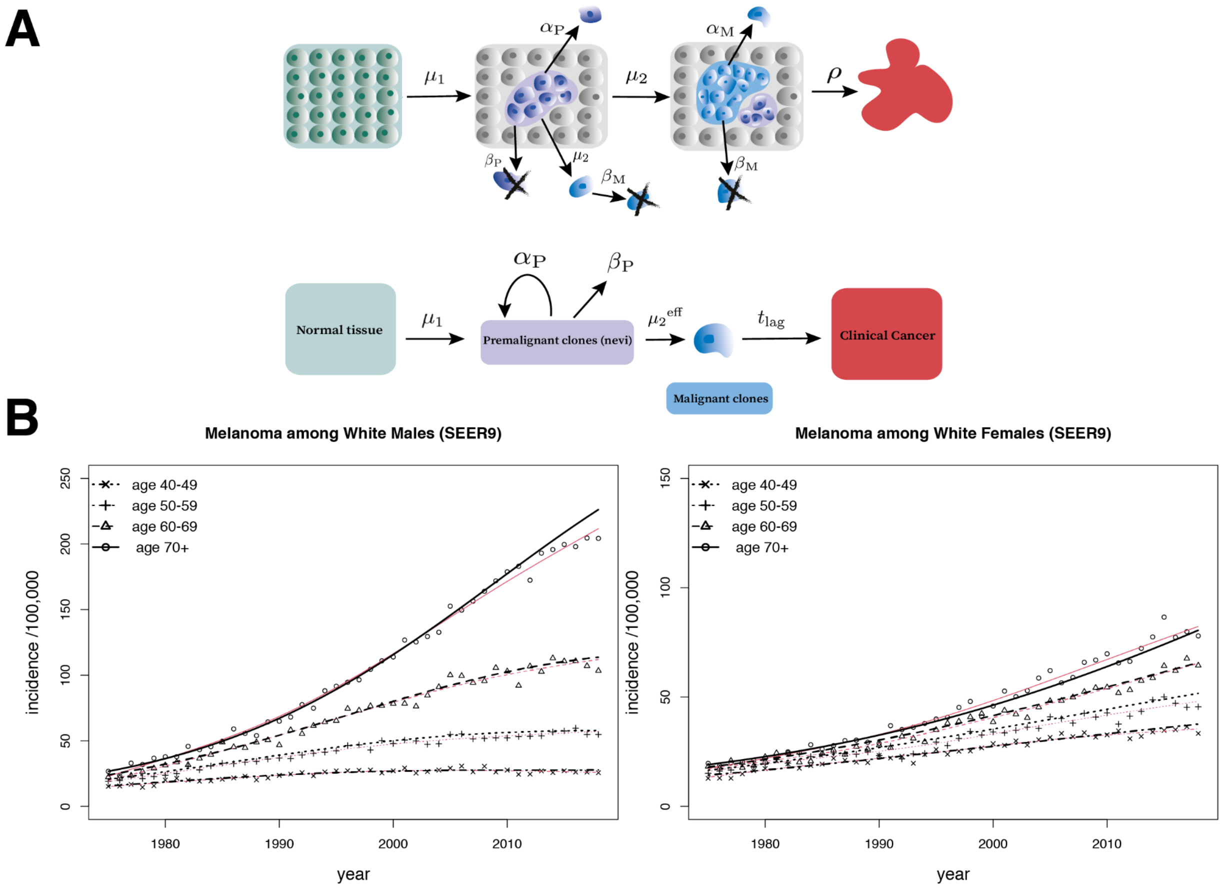

Figure S4: A) *Upper row*: Multistage clonal expansion (MSCE) model - illustration of a stochastic realization of the multi-type branching process. In the two-stage MSCE model, the first 'hit' occurs when normal cells (green) undergo asymmetric division (due to mutation, for example) and create one premalignant daughter cell with rate μ_1 and one normal cell. Premalignant cells can then clonally expand with rates α_P for division and β_P for cell death/differentiation (purple clones, e.g., dysplastic nevi). The second 'hit' occurs at rate μ_2 per cell per year wherein a premalignant cell creates a malignant daughter cell (due to secondary mutation, for example) and 1 premalignant cell. α_M and β_M represent cell division and death/differentiation, respectively, for malignant cells (blue). Clinical detection occurs for malignant clones with rate ρ. *Bottom row*: Mathematical approximation of the two-stage model includes an effective mutation rate μ_2^{eff} for transformation of a malignant cell that survives and a tumor sojourn time/ 'lag-time' representing the time between the first persistent malignant cell and the clinically detected cancer. B) Hazard curves from the MSCE models for melanoma incidence/100,000 (black lines) correspond to US SEER data from 1975-2018 (black shapes) in white females (right) and white males (left) separately by age groups, as represented by the different symbols, over calendar years. Calibrated parameters from the two-stage model yield similar average lag-time (tumor sojourn time) for males and females of ~10 years. For comparison, non-parametric fits using 4*th*-order smoothing splines shown in red.

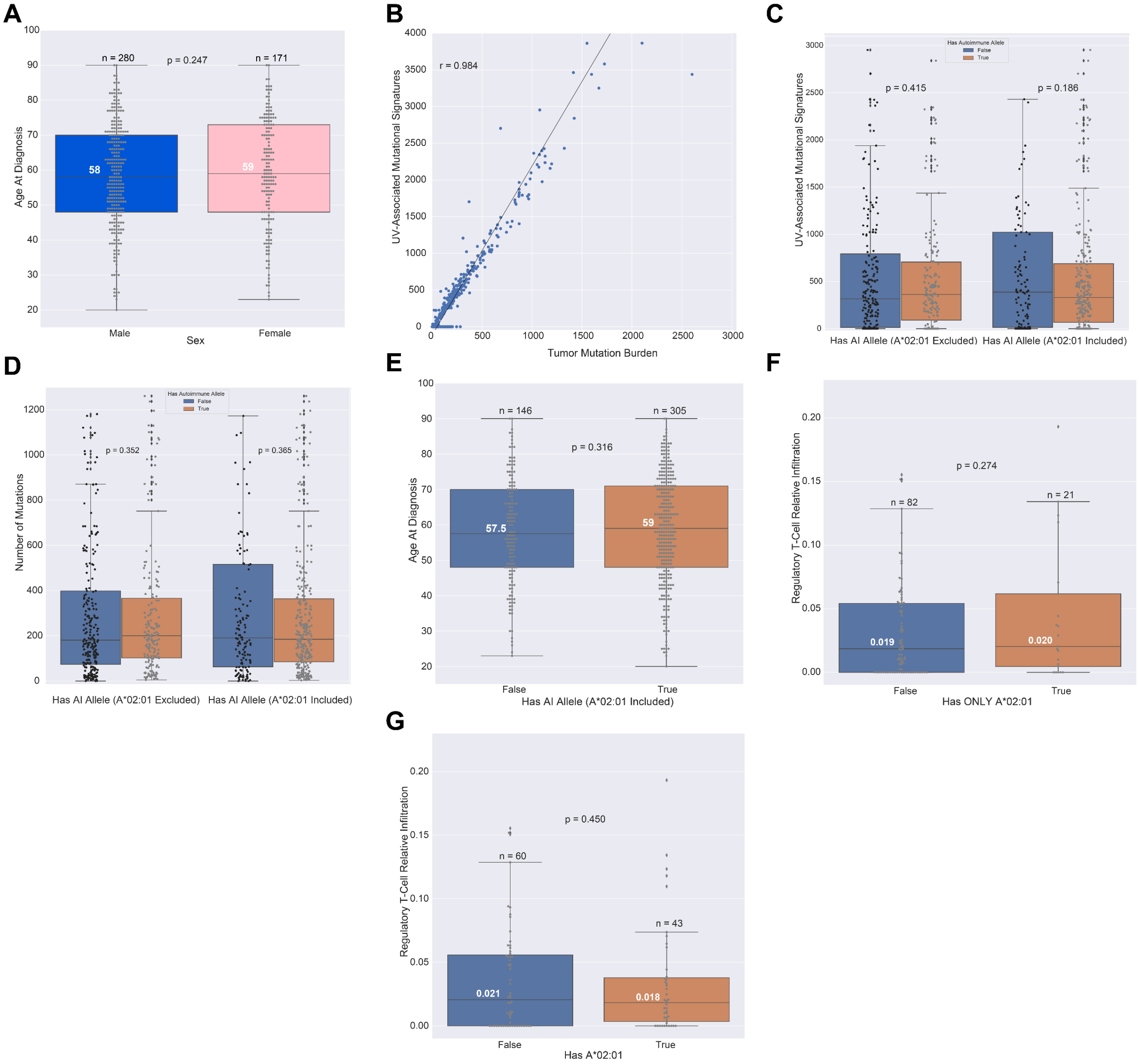

Figure S5: Covariates and associations with melanoma in the TCGA. A) Boxplots comparing sex and melanoma age of diagnosis. B) Correlation between total mutation burden and UV-signature associated mutations. C) Boxplots comparing the number of UV-signature associated mutations across those with and without a MHC-I linked autoimmune allele. D) Boxplots comparing the number of mutations across those with and without a MHC-I linked autoimmune allele. E) Effect of MHC-I autoimmune alleles on age at diagnosis in melanoma including HLA-A*02:01. F) Carriers of HLA-A*02:01 and no other autoimmune alleles do not exhibit a significant difference in regulatory T-cells in primary melanoma tumors. G) Carriers of HLA-A*02:01, independent of other alleles carried, do not exhibit a significant difference in regulatory T-cells in primary melanoma tumors.

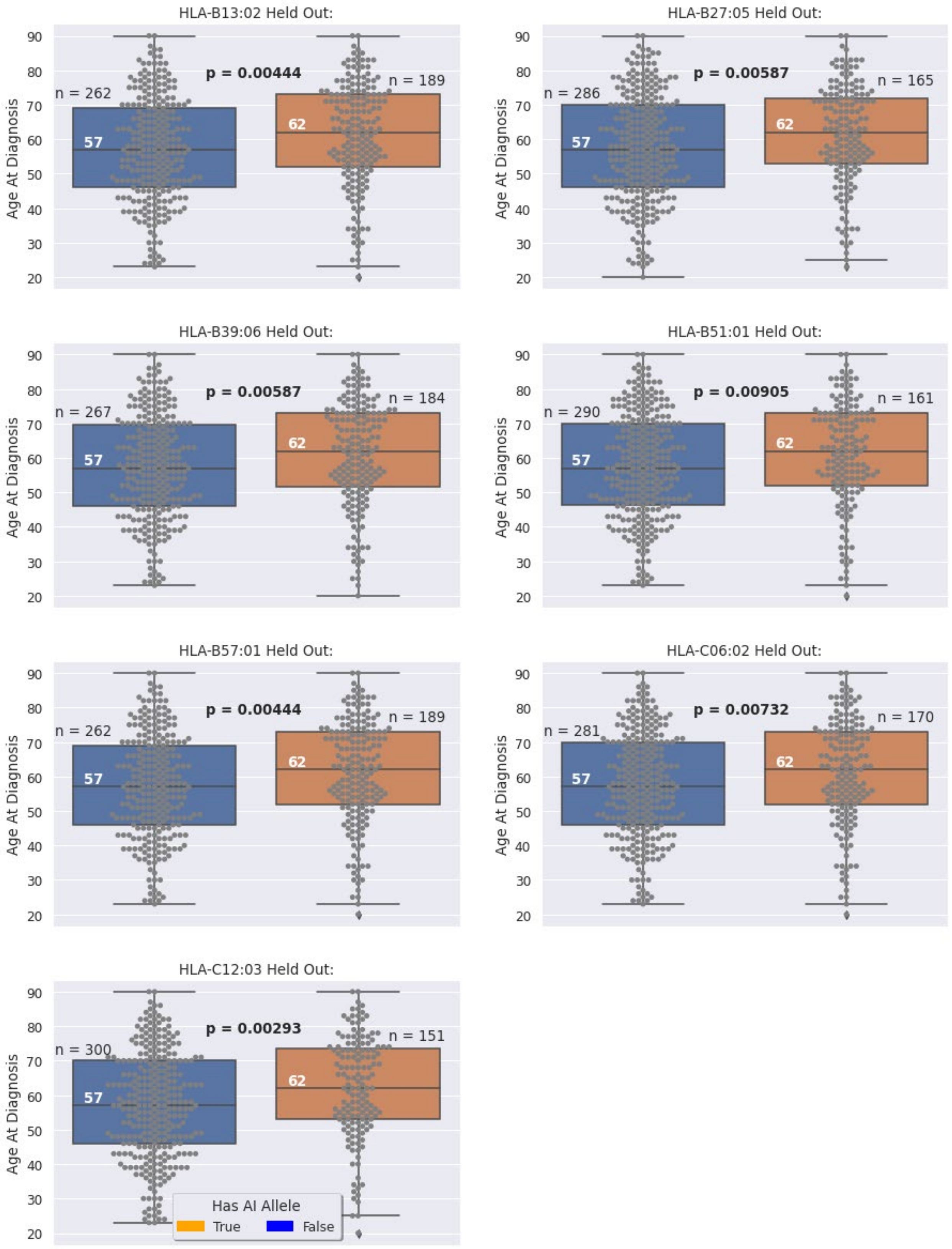
HLA-B13:02 Held Out:
p = 0.00444
n = 262
n = 189
57
62
HLA-B27:05 Held Out:
p = 0.00587
n = 286
n = 165
57
62
HLA-B39:06 Held Out:
p = 0.00587
n = 267
n = 184
57
62
HLA-B51:01 Held Out:
p = 0.00905
n = 290
n = 161
57
62
HLA-B57:01 Held Out:
p = 0.00444
n = 262
n = 189
57
62
HLA-C06:02 Held Out:
p = 0.00732
n = 281
n = 170
57
62
HLA-C12:03 Held Out:
p = 0.00293
n = 300
n = 151
57
62
Age At Diagnosis
90
80
70
60
50
40
30
20
Has AI Allele
True
False

Figure S6: Effect of omitting each of the 7 individual autoimmune alleles from carrier status in TCGA. For each autoimmune allele, individuals carrying only the excluded allele were assigned to non-AI allele carrier status. All p-values are multiple hypotheses corrected by means of the Benjamini-Hochberg method.

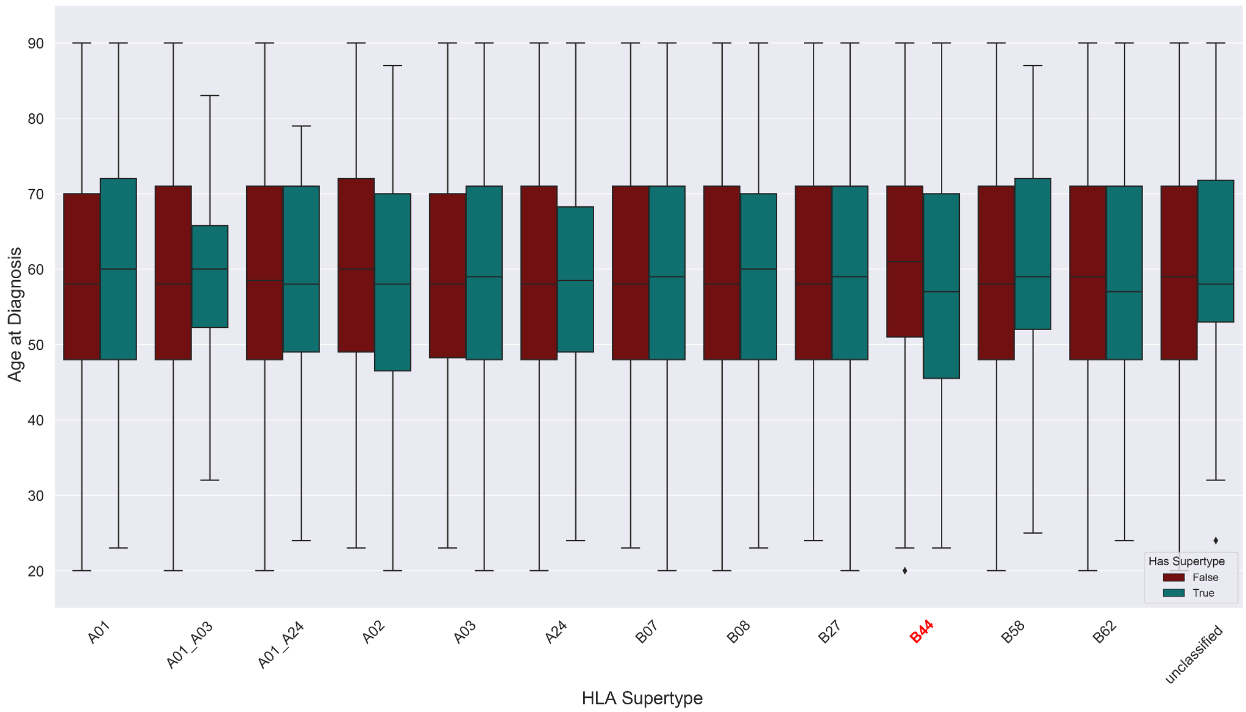

Figure S7: Effect of HLA supertype on age at diagnosis in TCGA. No significant age differences were observed across any supertype, though those with the B44 supertype (red) trended towards an earlier age of diagnosis (p = 0.199, median earlier age of diagnosis difference = 4 years).

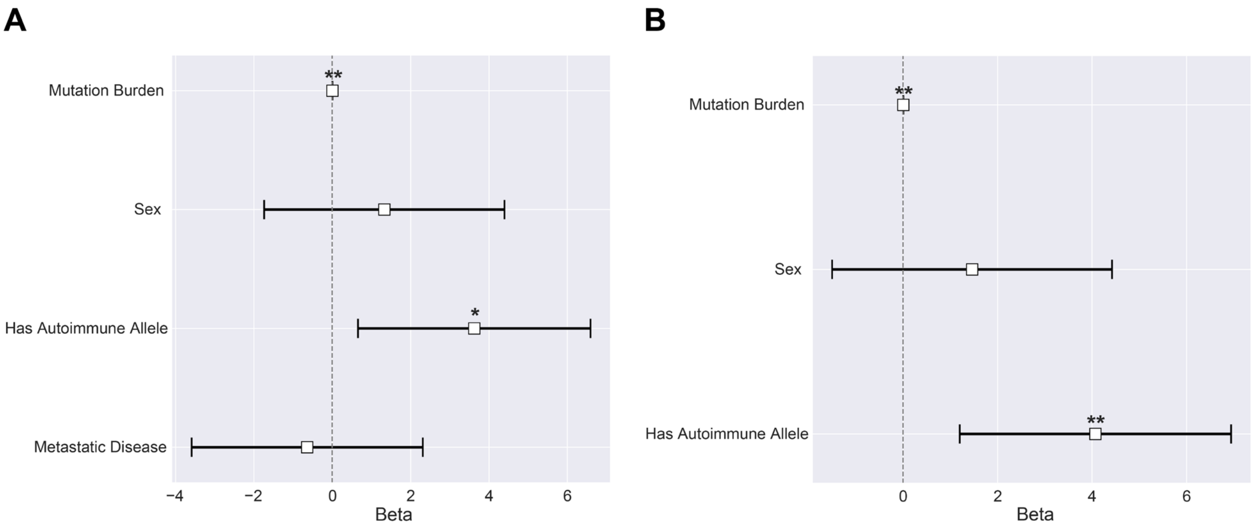

Figure S8: Regression modeling of autoimmune allele effects on melanoma age of diagnosis in TCGA. A) Having at least one MHC-I linked autoimmune allele is significantly associated with 3.62 delayed years to melanoma diagnosis after controlling for primary vs. metastatic disease, sex, and mutation burden (n = 416, $p_{autoimmune} = 0.015$). Those with an AJCC pathologic tumor stage of II or below were assigned a primary disease label, while those with a tumor stage of III or higher were assigned a metastatic disease label. B) Having at least one MHC-I linked autoimmune allele is significantly associated with 4.07 delayed years to melanoma diagnosis after controlling for sex and mutation burden (n = 451, $p_{autoimmune} = 0.005$).

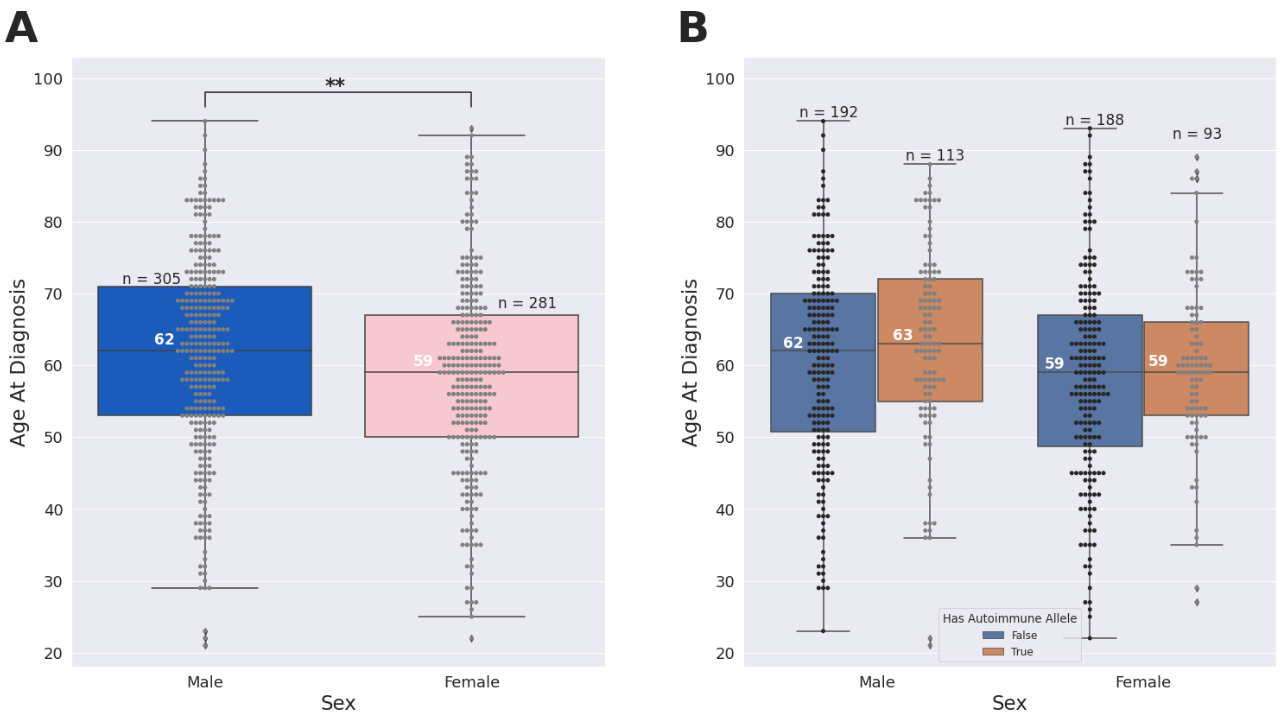

Figure S9: A) Females in the validation set showed a significant earlier age of diagnosis relative to males ($p = 0.003$, median difference = 3 years) B) Direction of AI carrier status effect in males is consistent with later age of diagnosis findings ($p_{males} = 0.055$, $p_{females} = 0.236$).

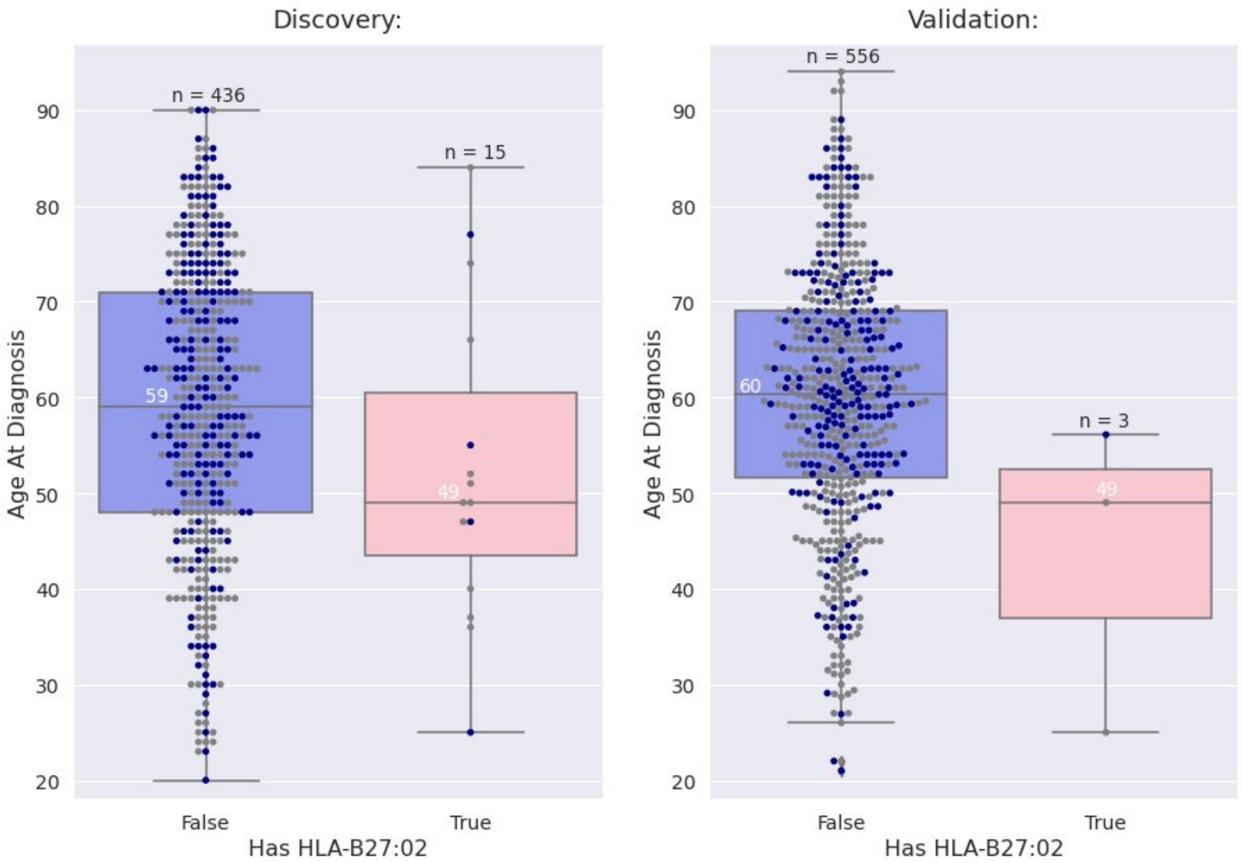

Figure S10: HLA-B*27:02 was associated with an earlier age of diagnosis across both discovery and validation sets. Blue points correspond to MHC-I autoimmune allele carriers, while gray points correspond to those without any MHC-I AI alleles.

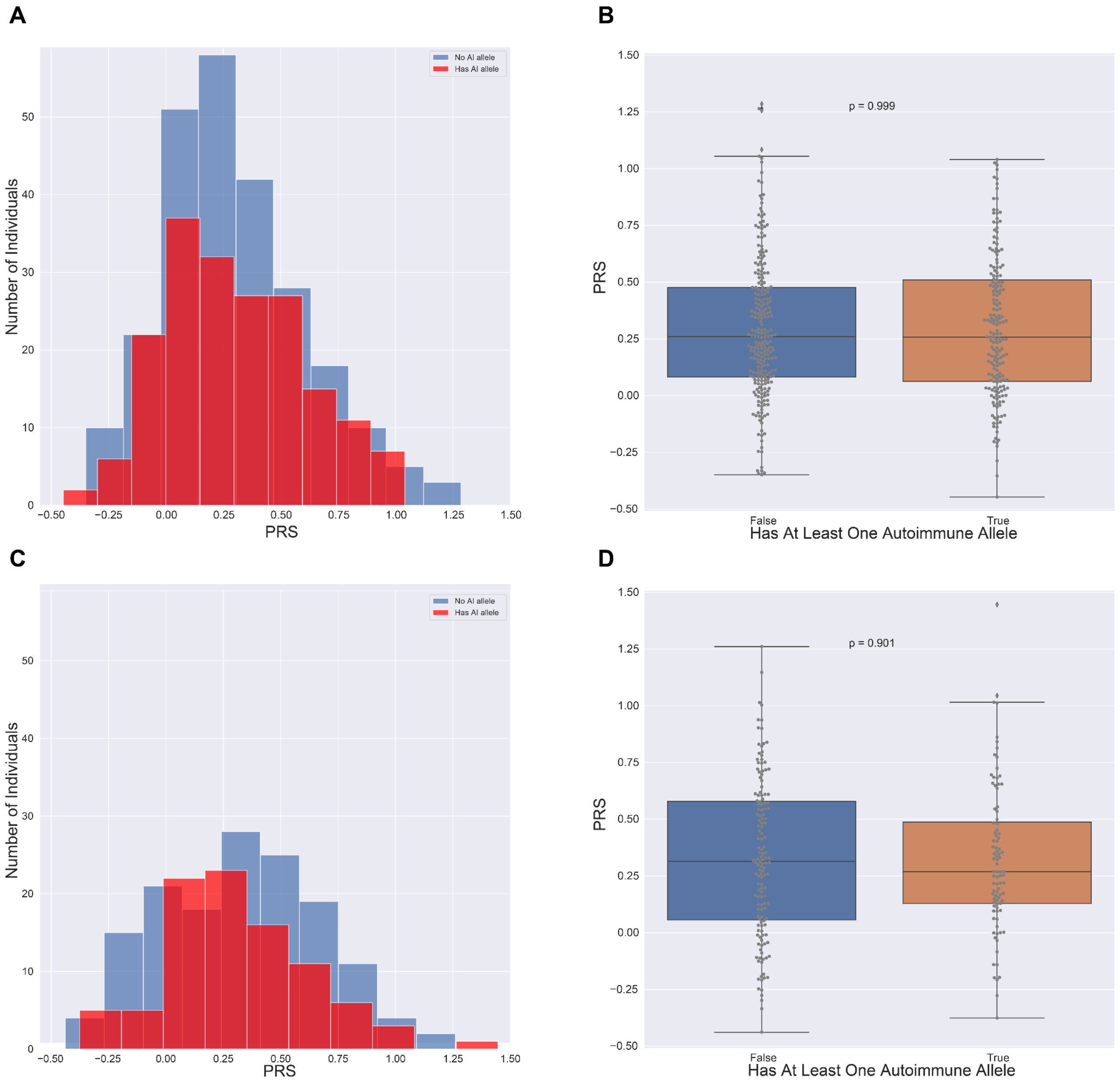

Figure S11: PRS and MHC-I autoimmune alleles exhibit independent effects with age. A) *TCGA*: Histogram of PRS stratified across individuals with and without MHC-I autoimmune alleles. B) *TCGA*: Boxplots stratifying PRS across individuals with and without MHC-I autoimmune alleles (p = 0.999, T-test). C) *Validation*: Histogram of PRS stratified across individuals with and without MHC-I autoimmune alleles. D) *Validation*: Boxplots stratifying PRS across individuals with and without MHC-I autoimmune alleles (p = 0.901, T-test).

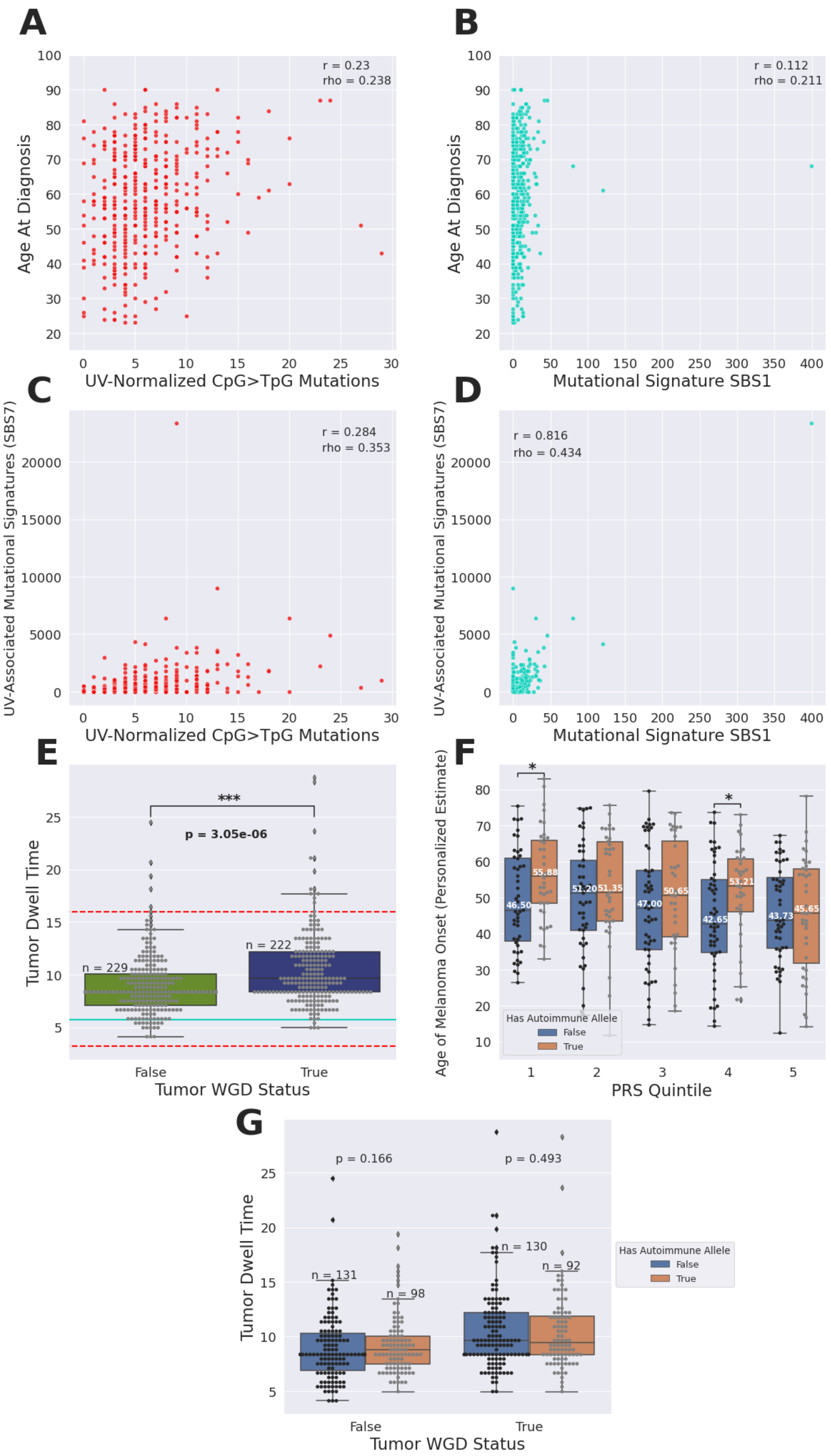
A
r = 0.23
rho = 0.238
Age At Diagnosis
UV-Normalized CpG>TpG Mutations
B
r = 0.112
rho = 0.211
Age At Diagnosis
Mutational Signature SBS1
C
r = 0.284
rho = 0.353
UV-Associated Mutational Signatures (SBS7)
UV-Normalized CpG>TpG Mutations
D
r = 0.816
rho = 0.434
UV-Associated Mutational Signatures (SBS7)
Mutational Signature SBS1
E
***
p = 3.05e-06
n = 229
n = 222
Tumor Dwell Time
False
True
Tumor WGD Status
F
*
*
46.50
55.88
51.20
51.35
47.00
50.65
42.65
53.21
43.73
45.65
Has Autoimmune Allele
False
True
Age of Melanoma Onset (Personalized Estimate)
PRS Quintile
G
p = 0.166
p = 0.493
n = 131
n = 98
n = 130
n = 92
Tumor Dwell Time
False
True
Tumor WGD Status
Has Autoimmune Allele
False
True

Figure S12: *TCGA:* A) Correlation between UV-mutation spectrum corrected CpG>TpG mutations and age of diagnosis. B) Correlation between mutational signature SBS1 and age of diagnosis. C) Correlation between UV-mutation spectrum corrected CpG>TpG mutations and UV-associated mutational signature SBS7. D) Correlation between mutational signature SBS1 and UV-associated mutational signature SBS7. E) Tumor dwell time in WGD and non-WGD melanomas. The solid blue line at 5.7 years equates to literature-reported estimated timing from WGD to melanoma diagnosis. The dotted red lines at 3.2 and 16 years represent the literature-reported WGD to melanoma diagnosis range. F) Boxplots of personalized estimated age of melanoma onset across PRS quintiles. Autoimmune MHC-I carrier status exhibited significant later predicted ages of onset for the lowest (PRS Quintile 1; $p = 0.011$) and second-highest risk quintiles (PRS Quintile 4; $p = 0.019$). G) Autoimmune MHC-I carriers do not show longer tumor dwell times, regardless of tumor WGD status.

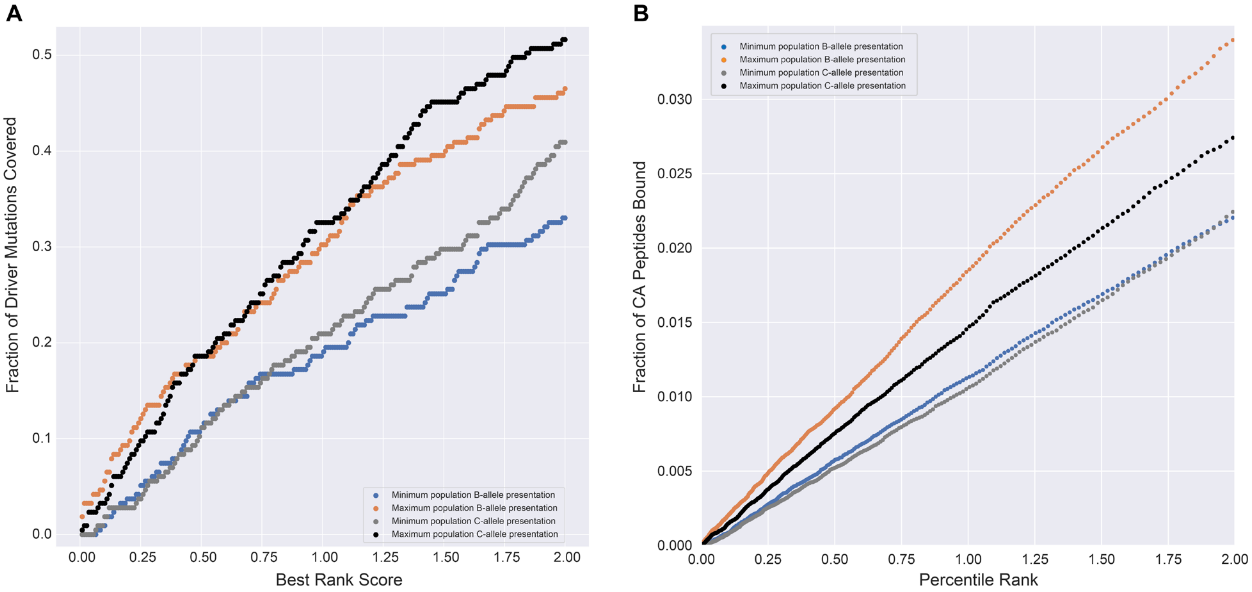

Figure S13: A) Maximum and minimum HLA-B population allele representations (Methods: *HLA Population Allele Representations*) exhibit greater neopeptide coverage than HLA-C maximum and minimum population allele representations at lower rank scores. As rank scores exceed the classical strong binding threshold (0.5), HLA-C population allele representations generally exhibit greater neopeptide coverage. B) For conserved antigens (CA), the maximum and minimum population allele representations for HLA-B presents a greater fraction of peptides than the maximum and minimum population allele representations for HLA-C. This is most pronounced in the maximum representations, where HLA-B presents a greater fraction of CA peptides compared to HLA-C as percentile rank increases.

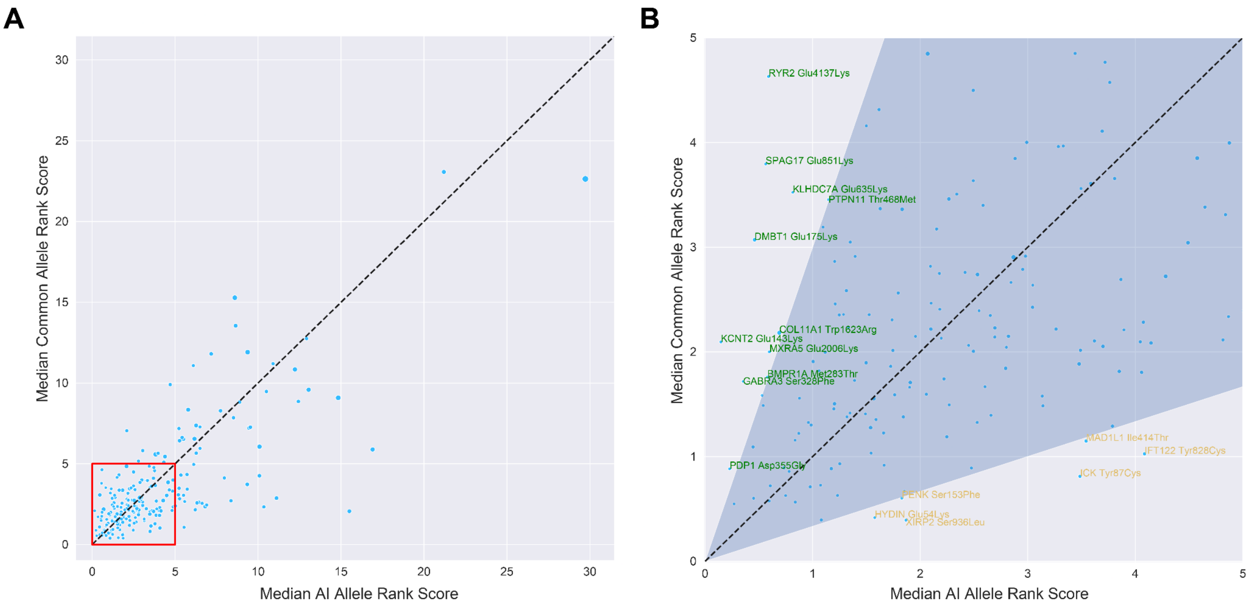

Figure S14: Predicted median driver mutation rank scores for both common and autoimmune MHC-I alleles. The varying point size reflects a mutation's standard deviation across all autoimmune alleles, with larger points having larger standard deviations. A) Predicted median rank scores for all driver mutations (n = 215) for both common and AI alleles. B) Predicted median rank scores for driver mutations in a presentable range (outlined in red in panel A). Mutations with a 3-fold median rank score discrepancy between AI and common alleles in either direction are labeled, and fall outside the shaded region. Green mutations correspond to mutations that are better presented by the median autoimmune allele, while yellow mutations correspond to mutations that are better presented by the median common allele.

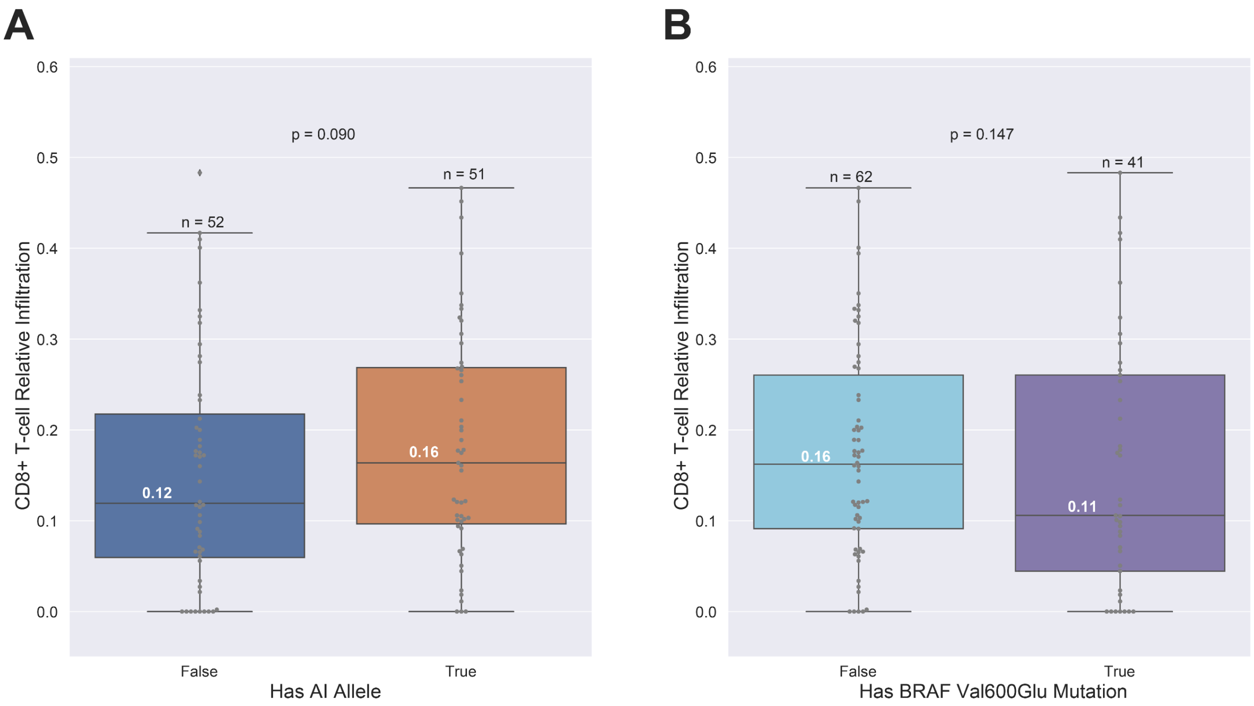

Figure S15: *TCGA*: A) CD8+ T-Cell infiltration in carriers of at least one MHC-I autoimmune allele against non-MHC-I autoimmune allele carriers in primary melanoma tumors. B) CD8+ T-Cell infiltration in individuals with a BRAF p.Val600Glu mutation against individuals without a BRAF p.Val600Glu mutation in primary melanoma tumors.

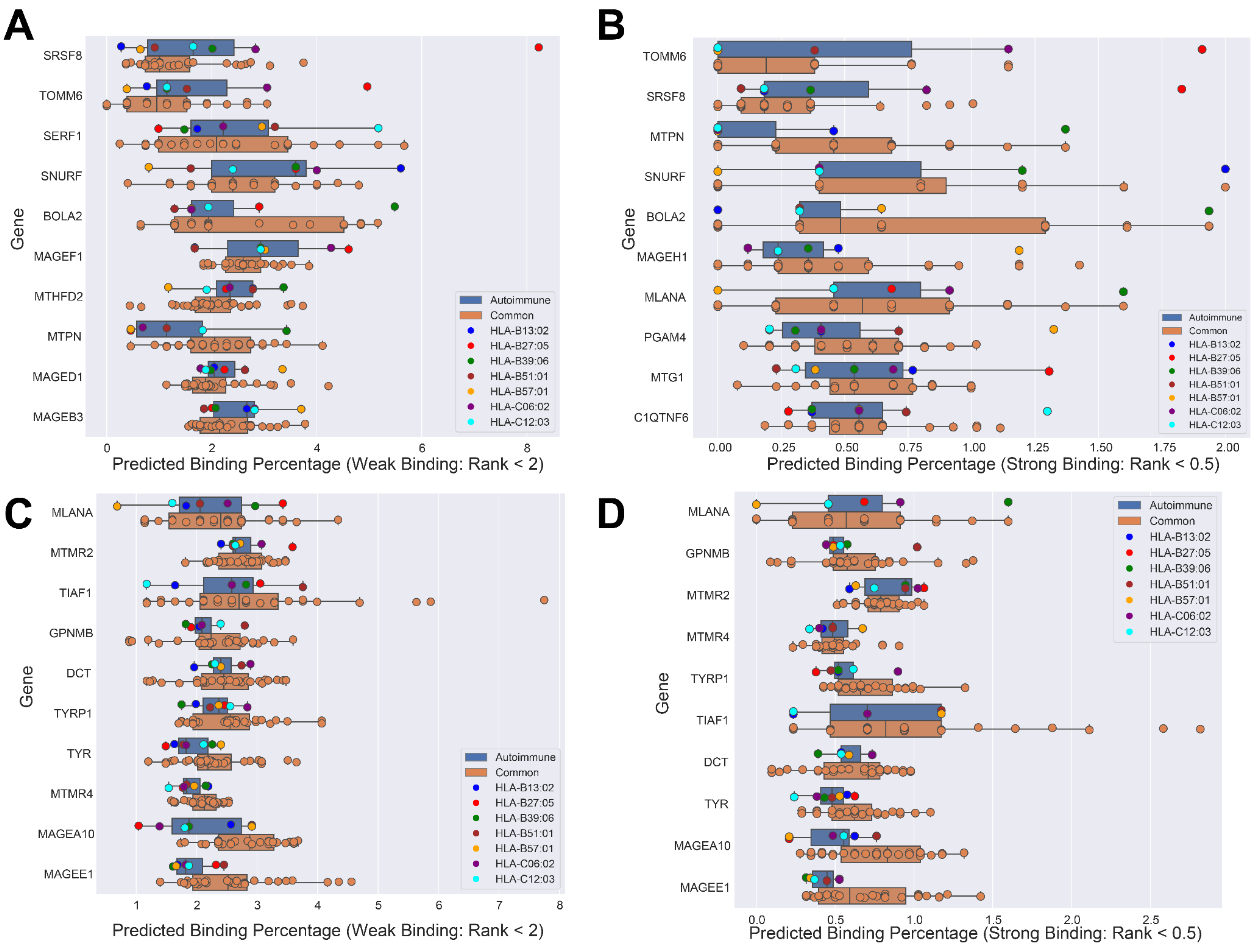

Figure S16: Distributions of the predicted binding repertoire of conserved antigens between MHC-I autoimmune and common alleles. A-B) Top 10 conserved antigens by difference in mean percentage binding (autoimmune - common). Percent binding at the A) 2% rank threshold and B) 0.5% rank threshold. C-D) Differentially expressed conserved antigens sorted as in panels A-B showing percentage binding at the C) 2% rank threshold and D) 0.5% rank threshold.

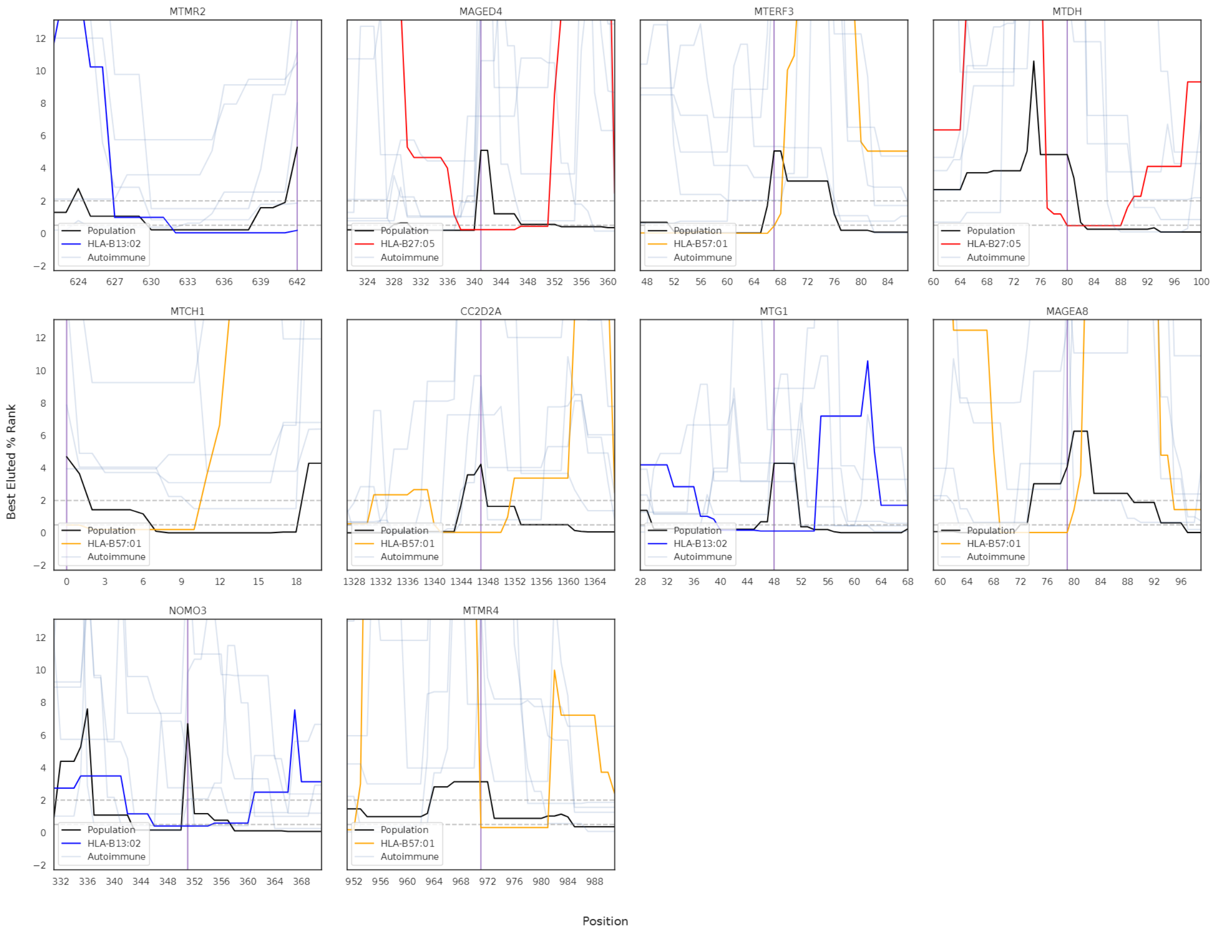

Figure S17: Greatest position-wise differences in predicted binding to conserved antigens between HLA-B autoimmune and common alleles amongst the top 8 differences (*MAGED4, MTERF3, MTDH, MTCH1, CC2D2A, MTG1, MAGEA8, NOMO3*) and DE conserved antigens (*MTMR2, MTMR4*) not shown in Figure S2B. Common alleles are shown in black and autoimmune alleles are shown in transparent blue unless they are predicted to elute at better percentile ranks than common alleles. Vertical purple lines demarcate positions where autoimmune alleles are predicted to elute at better percentile ranks than common alleles. Plots show up to +/- 20 amino acids of the demarcated positions.

	Number of Individuals	Average Age (± σ)	Sex (M/F)
TCGA	451	58.66 ± 15.28	280/171 (37.9%)
Van Allen et al. [1]	108	59 ± 15.96	76/32 (29.6%)
Hugo et al. [2]	35	61.46 ± 12.93	24/11 (31.4%)
The Genetic and Transcriptomic Evolution of Melanoma [3]	39	55.49 ± 16.55	22/17 (43.6%)
Melanoma Exome Sequencing [4,5]	138	66.37 ± 14.01	84/54 (39.1%)
UKBB	239	56.53 ± 10.47	79/160 (66.9%)

Table S1: Study specific statistics across both the discovery (TCGA) set and validation subsets. Study statistics are reported for individuals ≥ 20 years old, as individuals younger than this were excluded from analyses (See Methods*: Datasets*). Values reported in parentheses correspond to the percentage of females in the particular study.

HLA-Allele	Condition(s)	Reported Odds Ratio	Reference PMID/Citation
HLA-A*02:01	Vitiligo	2.07 (1.67-2.58) [6]	17243956
		1.58 [7]	20410501
		1.52 (1.21-1.90) [8]	27689083
HLA-B*13:02	Vitiligo	1.87 (1.23-2.84) [9]	15265531
		1.88 [10]	7351272
	Psoriasis	2.77 [11]	22577363
HLA-B*27:05	Ankylosing Spondylitis	> 50 [12]	Brown, M.A. & Xu H. "Genetics of axial spondyloarthritis" in *Rheumatology*, Sixth Edition. Ed. Hochberg M.C. (2015). Available at: https://doi.org/10.1016/B978-0-323-09138-1.00116-9
	Psoriasis	3.03 (2.14, 4.28) [13]	26289052
		3.39 (2.35, 4.89) [14]	23916976
	Vitiligo	2.29 (1.30-4.06) [9]	15265531
HLA-B*39:06	Type 1 Diabetes	10.31 [15]	20798335
	Psoriasis	1.46 (1.31–1.62) [16]	25087609
HLA-B*51:01	Behcet's disease	6.04 (4.96–7.35) [17]	Koné-Paut, I. "Chapter 19 - Behçet's Disease" in *Handbook of Systemic Autoimmune Diseases*. Ed. Cimaz, R. & Lehman, T. (2016) Available at: https://www.sciencedirect.com/science/article/pii/B9780444635969000190

		5-10 [18]	34690278
	Psoriasis	N/A [19]	16335880
HLA-B*57:01	Psoriasis	3.61 [11]	22577363
HLA-C*06:02	Vitiligo	3.04 (1.79-5.18) [9]	15265531
	Psoriasis	3.57 [11]	22577363
		3.26 (3.02-3.52) [16]	25087609
		3-10 [20]	29760713
		1.54-∞ [21]	10504461
HLA-C*12:03	Psoriasis	1.38 (1.26-1.52) [16]	25087609

Table S2: Summary of MHC-I linked autoimmune alleles including reported odds ratios for the autoimmune conditions with which they are associated.

	Discovery Set (TCGA) Allele Frequencies	Validation Set Allele Frequencies	Population Allele Frequency	Autoimmune Disease Association
HLA-A*02:01	26.27% (201)	25.31% (240)	27.6%	Vitiligo [22–25]
HLA-B*13:02	2.66% (23)	2.15% (23)	2.4%	Vitiligo [9,26]
HLA-B*27:05	3.66% (33)	4.20% (42)	3.7%	Psoriasis, Ankylosing Spondylitis [11,16,27–30]
HLA-B*39:06	1.00% (9)	0.63% (7)	0.64%	Psoriasis, Type 1 Diabetes[15,16,31,32]
HLA-B*51:01	4.21% (36)	3.49% (38)	4.7%	Psoriasis, Behcet's Disease[19,33]
HLA-B*57:01	4.21% (38)	3.22% (35)	3.6%	Psoriasis [11]
HLA-C*06:02	9.42% (82)	7.60% (80)	9.3%	Vitiligo, Psoriasis [9,26,34,35]
HLA-C*12:03	5.76% (51)	4.20% (44)	4.9%	Psoriasis [16,36]

Table S3: Autoimmune HLA allele frequency in both discovery (TCGA) and validation sets compared against the population distribution as reported by the European Caucasian subset of U.S. National Marrow Donor Program (N = 1,242,890). Values reported in parentheses correspond to the number of individuals in the dataset with the respective allele.

Supplementary References:

1. Van Allen, E.M., Miao, D., Schilling, B., Shukla, S.A., Blank, C., Zimmer, L., Sucker, A., Hillen, U., Foppen, M.H.G., Goldinger, S.M., et al. (2015). Genomic correlates of response to CTLA-4 blockade in metastatic melanoma. Science *350*, 207–211.

2. Hugo, W., Zaretsky, J.M., Sun, L., Song, C., Moreno, B.H., Hu-Lieskovan, S., Berent-Maoz, B., Pang, J., Chmielowski, B., Cherry, G., et al. (2016). Genomic and Transcriptomic Features of Response to Anti-PD-1 Therapy in Metastatic Melanoma. Cell *165*, 35–44.

3. Shain, A.H., Yeh, I., Kovalyshyn, I., Sriharan, A., Talevich, E., Gagnon, A., Dummer, R., North, J., Pincus, L., Ruben, B., et al. (2015). The Genetic Evolution of Melanoma from Precursor Lesions. N. Engl. J. Med. *373*, 1926–1936.

4. Krauthammer, M., Kong, Y., Bacchiocchi, A., Evans, P., Pornputtapong, N., Wu, C., McCusker, J.P., Ma, S., Cheng, E., Straub, R., et al. (2015). Exome sequencing identifies recurrent mutations in NF1 and RASopathy genes in sun-exposed melanomas. Nat. Genet. *47*, 996–1002.

5. Lazova, R., Pornputtapong, N., Halaban, R., Bosenberg, M., Bai, Y., Chai, H., and Krauthammer, M. (2017). Spitz nevi and Spitzoid melanomas: exome sequencing and comparison with conventional melanocytic nevi and melanomas. Mod. Pathol. *30*, 640–649.

6. Liu, J.-B., Li, M., Chen, H., Zhong, S.-Q., Yang, S., Du, W.-D., Hao, J.-H., Zhang, T.-S., Zhang, X.-J., and Zeegers, M.P. (2007). Association of vitiligo with HLA-A2: a meta-analysis. J. Eur. Acad. Dermatol. Venereol. *21*, 205–213.

7. Jin, Y., Birlea, S.A., Fain, P.R., Gowan, K., Riccardi, S.L., Holland, P.J., Mailloux, C.M., Sufit, A.J.D., Hutton, S.M., Amadi-Myers, A., et al. (2010). Variant of TYR and autoimmunity susceptibility loci in generalized vitiligo. N. Engl. J. Med. *362*, 1686–1697.

8. Li, Z., Ren, J., Niu, X., Xu, Q., Wang, X., Liu, Y., and Xiao, S. (2016). Meta-Analysis of the Association between Vitiligo and Human Leukocyte Antigen-A. Biomed Res. Int. *2016*, 5412806.

9. Zhang, X.-J., Liu, H.-S., Liang, Y.-H., Sun, L.-D., Wang, J.-Y., Yang, S., Liu, J.-B., Gao, M., He, P.-P., Cui, Y., et al. (2004). Association of HLA class I alleles with vitiligo in Chinese Hans. J. Dermatol. Sci. *35*, 165–168.

10. Metzker, A., Zamir, R., Gazit, E., David, M., and Feuerman, E.J. (1980). Vitiligo and the HLA system. Dermatologica *160*, 100–105.

11. Chen, H., Hayashi, G., Lai, O.Y., Dilthey, A., Kuebler, P.J., Wong, T.V., Martin, M.P., Fernandez Vina, M.A., McVean, G., Wabl, M., et al. (2012). Psoriasis patients are enriched for genetic variants that protect against HIV-1 disease. PLoS Genet. *8*, e1002514.

12. Brown, M.A., and Xu, H. (2015). Genetics of axial spondyloarthritis. In Rheumatology, M.C. Hochberg, ed. pp. 956–959.

13. Queiro, R., Morante, I., Cabezas, I., and Acasuso, B. (2016). HLA-B27 and psoriatic disease: a modern view of an old relationship. Rheumatology *55*, 221–229.

14. Chandran, V., Bull, S.B., Pellett, F.J., Ayearst, R., Rahman, P., and Gladman, D.D. (2013). Human leukocyte antigen alleles and susceptibility to psoriatic arthritis. Hum. Immunol. *74*, 1333–1338.

15. Noble, J.A., Valdes, A.M., Varney, M.D., Carlson, J.A., Moonsamy, P., Fear, A.L., Lane, J.A., Lavant, E., Rappner, R., Louey, A., et al. (2010). HLA class I and genetic susceptibility to type 1 diabetes: results from the Type 1 Diabetes Genetics Consortium. Diabetes *59*, 2972–2979.

16. Okada, Y., Han, B., Tsoi, L.C., Stuart, P.E., Ellinghaus, E., Tejasvi, T., Chandran, V., Pellett, F., Pollock, R., Bowcock, A.M., et al. (2014). Fine mapping major histocompatibility complex associations in psoriasis and its clinical subtypes. Am. J. Hum. Genet. *95*, 162–172.

17. Koné-Paut, I. (2016). Chapter 19 - Behçet's Disease. Handbook of Systemic Autoimmune Diseases *11*, 409–426.

18. Takeno, M. (2022). The association of Behçet's syndrome with HLA-B51 as understood in 2021. Curr. Opin. Rheumatol. *34*, 4–9.

19. Yamamoto, T., Yokozeki, H., and Nishioka, K. (2005). Psoriasis arthropathy and HLA-B51: report of 5 cases. J. Dermatol. *32*, 606–610.

20. Prinz, J.C. (2018). Human Leukocyte Antigen-Class I Alleles and the Autoreactive T Cell Response in Psoriasis Pathogenesis. Front. Immunol. *9*, 954.

21. Mallon, E., Newson, R., and Bunker, C.B. (1999). HLA-Cw6 and the genetic predisposition to psoriasis: a meta-analysis of published serologic studies. J. Invest. Dermatol. *113*, 693–695.

22. Ogg, G.S., Rod Dunbar, P., Romero, P., Chen, J.L., and Cerundolo, V. (1998). High frequency of skin-homing melanocyte-specific cytotoxic T lymphocytes in autoimmune vitiligo. J. Exp. Med. *188*, 1203–1208.

23. Lang, K.S., Caroli, C.C., Muhm, A., Wernet, D., Moris, A., Schittek, B., Knauss-Scherwitz, E., Stevanovic, S., Rammensee, H.G., and Garbe, C. (2001). HLA-A2 restricted, melanocyte-specific CD8(+) T lymphocytes detected in vitiligo patients are related to disease activity and are predominantly directed against MelanA/MART1. J. Invest. Dermatol. *116*, 891–897.

24. Palermo, B., Campanelli, R., Garbelli, S., Mantovani, S., Lantelme, E., Brazzelli, V., Ardigó, M., Borroni, G., Martinetti, M., Badulli, C., et al. (2001). Specific cytotoxic T lymphocyte responses against Melan-A/MART1, tyrosinase and gp100 in vitiligo by the use of major histocompatibility complex/peptide tetramers: the role of cellular immunity in the etiopathogenesis of vitiligo. J. Invest. Dermatol. *117*, 326–332.

25. Jin, Y., Ferrara, T., Gowan, K., Holcomb, C., Rastrou, M., Erlich, H.A., Fain, P.R., and Spritz, R.A. (2012). Next-generation DNA re-sequencing identifies common variants of TYR and HLA-A that modulate the risk of generalized vitiligo via antigen presentation. J. Invest. Dermatol. *132*, 1730–1733.

26. Quan, C., Ren, Y.-Q., Xiang, L.-H., Sun, L.-D., Xu, A.-E., Gao, X.-H., Chen, H.-D., Pu, X.-M., Wu, R.-N., Liang, C.-Z., et al. (2010). Genome-wide association study for vitiligo identifies susceptibility loci at 6q27 and the MHC. Nat. Genet. *42*, 614–618.

27. Winchester, R., Minevich, G., Steshenko, V., Kirby, B., Kane, D., Greenberg, D.A., and FitzGerald, O. (2012). HLA associations reveal genetic heterogeneity in psoriatic arthritis and in the psoriasis phenotype. Arthritis Rheum. *64*, 1134–1144.

28. FitzGerald, O., Haroon, M., Giles, J.T., and Winchester, R. (2015). Concepts of pathogenesis in psoriatic arthritis: genotype determines clinical phenotype. Arthritis Res. Ther. *17*, 115.

29. Brewerton, D.A., Hart, F.D., Nicholls, A., Caffrey, M., James, D.C., and Sturrock, R.D. (1973). Ankylosing spondylitis and HL-A 27. Lancet *1*, 904–907.

30. Brewerton, D.A., Caffrey, M., Nicholls, A., Walters, D., and James, D.C. (1974). HL-A 27 and arthropathies associated with ulcerative colitis and psoriasis. Lancet *1*, 956–958.

31. Nejentsev, S., Howson, J.M.M., Walker, N.M., Szeszko, J., Field, S.F., Stevens, H.E., Reynolds, P., Hardy, M., King, E., Masters, J., et al. (2007). Localization of type 1 diabetes susceptibility to the MHC class I genes HLA-B and HLA-A. Nature *450*, 887–892.

32. Howson, J.M.M., Walker, N.M., Clayton, D., Todd, J.A., and Type 1 Diabetes Genetics Consortium (2009). Confirmation of HLA class II independent type 1 diabetes associations in the major histocompatibility complex including HLA-B and HLA-A. Diabetes Obes. Metab. *11 Suppl 1*, 31–45.

33. Ohno, S., Ohguchi, M., Hirose, S., Matsuda, H., Wakisaka, A., and Aizawa, M. (1982). Close Association of HLA-Bw51 With Behcet's Disease. Archives of Ophthalmology *100*, 1455–1458.

34. Nair, R.P., Stuart, P.E., Nistor, I., Hiremagalore, R., Chia, N.V.C., Jenisch, S., Weichenthal, M., Abecasis, G.R., Lim, H.W., Christophers, E., et al. (2006). Sequence and haplotype analysis supports HLA-C as the psoriasis susceptibility 1 gene. Am. J. Hum. Genet. *78*, 827–851.

35. Gudjonsson, J.E., Karason, A., Antonsdottir, A., Runarsdottir, E.H., Hauksson, V.B., Upmanyu, R., Gulcher, J., Stefansson, K., and Valdimarsson, H. (2003). Psoriasis patients who are homozygous for the HLA-Cw*0602 allele have a 2.5-fold increased risk of developing psoriasis compared with Cw6 heterozygotes. Br. J. Dermatol. *148*, 233–235.

36. Helms, C., Saccone, N.L., Cao, L., Daw, J.A.W., Cao, K., Hsu, T.M., Taillon-Miller, P., Duan, S., Gordon, D., Pierce, B., et al. (2005). Localization of PSORS1 to a haplotype block harboring HLA-C and distinct from corneodesmosin and HCR. Hum. Genet. *118*, 466–476.
