# Supplementary material for: Autoimmune alleles at the major histocompatibility locus modify melanoma susceptibility
Source: Am J Hum Genet. 2023 Jun 19;110(7):1138–61. doi: 10.1016/j.ajhg.2023.05.013 (PMC10357503; doi:10.1016/j.ajhg.2023.05.013)
Supplement: Document S2. Article plus supplemental information [file mmc3.pdf]

Autoimmune alleles at the major histocompatibility locus modify melanoma susceptibility

Graphical abstract

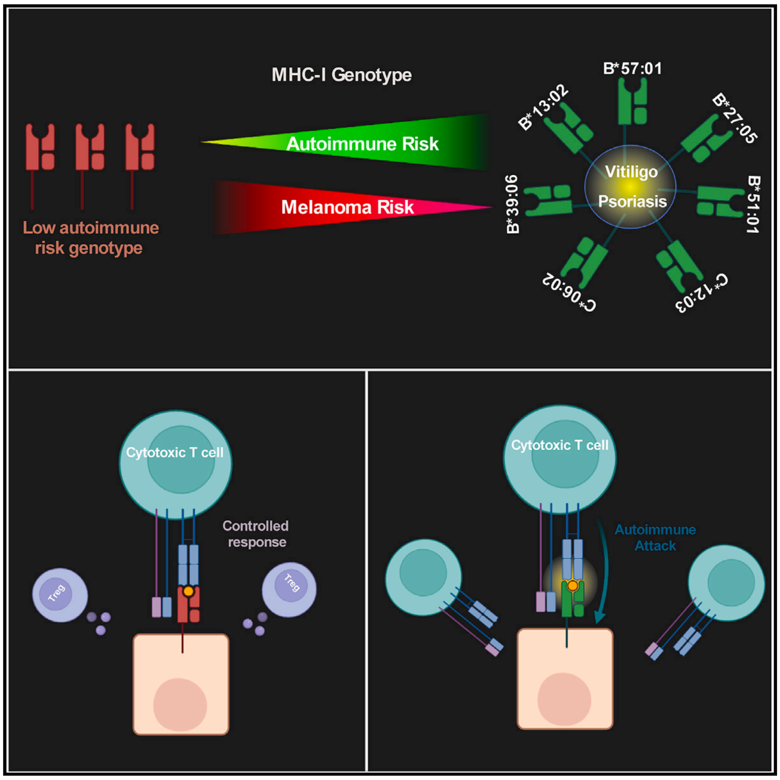

Authors

James V. Talwar, David Laub, Meghana S. Pagadala, ..., Kit Curtius, Maurizio Zanetti, Hannah Carter

Correspondence

hkcarter@health.ucsd.edu

Talwar et al. demonstrate that carrying vitiligo- and psoriasis-predisposing MHC-I alleles provides a protective effect against melanoma unaccounted for by current PRSs. This finding, coupled with an investigation into its mechanisms, suggests the potential for a broader MHC-mediated autoimmune-cancer risk interplay.

Talwar et al., 2023, The American Journal of Human Genetics *110*, 1138–1161
July 6, 2023 © 2023 The Authors.
https://doi.org/10.1016/j.ajhg.2023.05.013

ARTICLE

Autoimmune alleles at the major histocompatibility locus modify melanoma susceptibility

James V. Talwar,[1,2] David Laub,[1,2] Meghana S. Pagadala,[3] Andrea Castro,[1,2] McKenna Lewis,[4] Georg E. Luebeck,[5] Bryan R. Gorman,[6,7] Cuiping Pan,[8] Frederick N. Dong,[6,7] Kyriacos Markianos,[6,9,10,11] Craig C. Teerlink,[12,13] Julie Lynch,[12,13] Richard Hauger,[14,15,16] Saiju Pyarajan,[6,17,18] Philip S. Tsao,[8,19] Gerald P. Morris,[20] Rany M. Salem,[21] Wesley K. Thompson,[22] Kit Curtius,[2,23,24] Maurizio Zanetti,[23,25,26] and Hannah Carter[1,2,23,*]

Summary

Autoimmunity and cancer represent two different aspects of immune dysfunction. Autoimmunity is characterized by breakdowns in immune self-tolerance, while impaired immune surveillance can allow for tumorigenesis. The class I major histocompatibility complex (MHC-I), which displays derivatives of the cellular peptidome for immune surveillance by $CD8^+$ T cells, serves as a common genetic link between these conditions. As melanoma-specific $CD8^+$ T cells have been shown to target melanocyte-specific peptide antigens more often than melanoma-specific antigens, we investigated whether vitiligo- and psoriasis-predisposing MHC-I alleles conferred a melanoma-protective effect. In individuals with cutaneous melanoma from both The Cancer Genome Atlas ($n = 451$) and an independent validation set ($n = 586$), MHC-I autoimmune-allele carrier status was significantly associated with a later age of melanoma diagnosis. Furthermore, MHC-I autoimmune-allele carriers were significantly associated with decreased risk of developing melanoma in the Million Veteran Program ($OR = 0.962$, $p = 0.024$). Existing melanoma polygenic risk scores (PRSs) did not predict autoimmune-allele carrier status, suggesting these alleles provide orthogonal risk-relevant information. Mechanisms of autoimmune protection were neither associated with improved melanoma-driver mutation association nor improved gene-level conserved antigen presentation relative to common alleles. However, autoimmune alleles showed higher affinity relative to common alleles for particular windows of melanocyte-conserved antigens and loss of heterozygosity of autoimmune alleles caused the greatest reduction in presentation for several conserved antigens across individuals with loss of HLA alleles. Overall, this study presents evidence that MHC-I autoimmune-risk alleles modulate melanoma risk unaccounted for by current PRSs.

Introduction

The incidence of cutaneous melanoma (MIM: 155600), the most common form, has seen an increase globally, particularly in Western countries.[1,2] Early detection is a major determinant of overall disease prognosis with the 5-year survival rate dropping precipitously from 99% to 27.3% for local versus distant disease, respectively.[3] Models developed for early disease detection are often built around well-known environmental and host risk factors including ultraviolet radiation exposure,[4–6] pigmentary phenotypes,[7–9] melanocytic nevi count,[10,11] sex,[12,13] age,[12,13] telomere length,[14,15] immunosuppression,[16,17] and family history.[18,19] However, though cutaneous melanoma ranks among the most heritable forms of cancer with an estimated heritability of 58%,[20] the majority of genetic susceptibility remains unaccounted for.

Cutaneous melanoma is also considered among the most immunogenic forms of cancer. Melanoma exhibits one of the highest mutation burdens across cancers, which is driven primarily by the mutagenic influence of ultraviolet radiation exposure.[21,22] This increases the number of neoepitopes presented to the immune system and plays an essential role in immune surveillance. Immunosuppression,

[1]Department of Medicine, Division of Medical Genetics, University of California San Diego, La Jolla, CA 92093, USA; [2]Bioinformatics and Systems Biology Program, University of California San Diego, La Jolla, CA 92093, USA; [3]Biomedical Science Program, University of California San Diego, La Jolla, CA 92093, USA; [4]Department of Computer Science and Engineering, University of California San Diego, La Jolla, CA 92093, USA; [5]Public Health Sciences Division, Herbold Computational Biology Program, Fred Hutchinson Cancer Research Center, Seattle, WA 98109, USA; [6]Center for Data and Computational Sciences (C-DACS), VA Boston Healthcare System, Boston, MA 02130, USA; [7]Booz Allen Hamilton, Inc., McLean, VA 22102, USA; [8]Palo Alto Epidemiology Research and Information Center for Genomics, VA Palo Alto, CA, USA; [9]Division of Genetics and Genomics, Department of Pediatrics, Boston Children's Hospital, Boston, MA 02115, USA; [10]Department of Pediatrics, Harvard Medical School, Boston, MA 02115, USA; [11]Broad Institute of Harvard and MIT, Cambridge, MA 02115, USA; [12]Department of Veterans Affairs Informatics and Computing Infrastructure (VINCI), VA Salt Lake City Healthcare System, Salt Lake City, UT, USA; [13]Department of Internal Medicine, Division of Epidemiology, University of Utah School of Medicine, Salt Lake City, UT, USA; [14]VA San Diego Healthcare System, La Jolla, CA, USA; [15]Center for Behavioral Genetics of Aging, University of California San Diego, La Jolla, CA, USA; [16]Center of Excellence for Stress and Mental Health (CESAMH), VA San Diego Healthcare System, San Diego, CA, USA; [17]Department of Medicine, Brigham Women's Hospital, Boston, MA, USA; [18]Department of Medicine, Harvard Medical School, Boston, MA, USA; [19]Department of Medicine, Stanford University School of Medicine, Stanford, CA, USA; [20]Department of Pathology, University of California San Diego, La Jolla, CA 92093, USA; [21]Division of Epidemiology, Herbert Wertheim School of Public Health and Human Longevity Science, University of California San Diego, La Jolla, CA 92093, USA; [22]Center for Population Neuroscience and Genetics, Laureate Institute for Brain Research, Tulsa, OK 74136, USA; [23]Moores Cancer Center, University of California San Diego, La Jolla, CA 92093, USA; [24]Division of Biomedical Informatics, Department of Medicine, University of California San Diego, La Jolla, CA 92093, USA; [25]The Laboratory of Immunology, University of California San Diego, La Jolla, CA 92093, USA; [26]Department of Medicine, Division of Hematology and Oncology, University of California San Diego, La Jolla, CA 92093, USA
*Correspondence: hkcarter@health.ucsd.edu
https://doi.org/10.1016/j.ajhg.2023.05.013.
© 2023 The Authors. This is an open access article under the CC BY license (http://creativecommons.org/licenses/by/4.0/).

though, impairs the immune system's cytotoxic potential and is a documented risk factor for increased melanoma incidence.[16,17] Lymphocyte infiltration and melanoma-specific antibodies have been shown to be powerful prognostic factors as well.[23,24] Immune traits themselves show considerable heritability,[25–27] and early investigations suggest that heritable immune alleles also contribute to melanoma risk.[28–30] In contrast to cancer, where poor immune function is a risk factor,[31,32] increased sensitivity of the immune system can lead to autoimmune disorders.[33] This dichotomy has led to speculation that induction of autoimmunity in individuals being treated for cancer could lead to tumor regression and better immunotherapy efficacy,[34–39] though studies investigating the relationship between autoimmunity and cancer risk have returned mixed findings.[40–42] If autoimmune alleles can enhance host anti-tumor immune responses when immunotherapy is administered, it is possible that they could also enhance anti-tumor immunity more generally. Thus, we speculated that autoimmune alleles, particularly those related to T cell responses directed against melanocyte or melanoma-specific antigens, might modify polygenic risk for melanoma in ways that might not be captured by current polygenic risk scores (PRSs).

The class I major histocompatibility complex (MHC-I [MIM: 142800]) represents a fundamental component of the antigen-directed immune response common to cancer and autoimmunity. MHC-I binds and displays peptide antigens derived primarily from intracellular proteins on the cell surface for immune surveillance by $CD8^+$ T cells.[43] In cancer, neopeptides containing somatic mutations unique to the tumor genome, when displayed by MHC-I, can be recognized as foreign by $CD8^+$ T cells, triggering the release of cytotoxic granules.[44,45] The peptide-binding specificity of MHC-I is determined by three highly polymorphic genes, *HLA-A* (MIM: 142800), *HLA-B* (MIM: 142830), and *HLA-C* (MIM: 142840), encoded at the human leukocyte antigen (HLA) locus on chromosome 6. The specific set of HLA alleles carried by an individual has been found to impose selective constraints on the developing tumor genome[46–48] and modify response to immunotherapy.[49–51] MHC-I loss, often due to deletion or mutation of HLA genes, is one mechanism of immune evasion during tumor development.[52,53]

MHC-I also plays a role in several skin-specific autoimmune disorders. In vitiligo (MIM: 606579), destruction of melanocytes and consequent loss of skin pigmentation is mediated by $CD8^+$ T cell responses to self-antigens displayed by MHC-I.[54,55] Another skin autoimmune disorder involving $CD8^+$ T cell responses is psoriasis (MIM: 177900),[56,57] which is characterized by dermal leukocyte infiltration and hyperproliferation of keratinocytes.[57] $CD8^+$ T cells in psoriasis have been shown to target melanocytes in affected individuals carrying particular MHC-I alleles.[58,59] Both of these conditions also share a risk variant, rs9468925, in *HLA-B*/*HLA-C*,[60] supporting a shared MHC-I-driven etiology, which is consistent with disease co-occurrence findings in clinical investigations.[61,62]

Intriguingly, multiple vitiligo risk alleles implicated by genome-wide association studies have been shown to exhibit protection from cutaneous melanoma,[28,29] and emergence of vitiligo during immunotherapy treatment of melanoma-affected individuals has been associated with better responses.[63–66] Though psoriasis has also been documented as an immune-related adverse effect of immunotherapy, the association with melanoma prognosis is far less characterized.[67,68]

Altogether, these findings support the potential for class I autoimmune HLA alleles to modify melanoma risk and inform risk prediction. To further investigate this possibility, we evaluated autoimmune HLA carrier status in cutaneous melanoma samples from The Cancer Genome Atlas (TCGA). Skin autoimmune alleles were associated with a significantly later age at diagnosis among melanoma cases, which was recapitulated in an independent validation set of 586 individuals assembled from the UK Biobank (UKBB) and four other published melanoma genome sequencing studies. Moreover, these findings generalized to melanoma incidence with autoimmune HLA carriers significantly associating with a decreased risk of developing melanoma in the Million Veteran Program cohort. Finally, we investigated the peptide specificity of autoimmune alleles for a set of 215 melanoma-specific driver mutations spanning 172 genes and for conserved and cancer antigens previously implicated in melanoma-directed immunity. These analyses highlight a protective role for MHC-I in the context of melanoma development.

Material and methods

Datasets

TCGA skin cutaneous melanoma tumors (SKCMs) were used as the discovery set (n = 470). Cases were retained if they had appropriate clinical information for downstream analysis (i.e., age of diagnosis; 11 did not have this information), were microsatellite stable (three MSI tumors were removed), and were at least 20 years of age at time of diagnosis (five were <20). Individuals below 20 years of age were excluded because of their increased likelihood of harboring rare predisposing risk variants. After filtering, there were 451 tumor samples, the majority of which were from individuals of European ancestry (n = 433/451). Other ancestries represented were Asian (n = 12) and African American (n = 1), and the remaining few were unknown. MHC-I genotypes were called with the exome-based methods POLYSOLVER and HLA-HD.[69,70]

An independent validation set of melanoma cases with germline whole-exome sequencing/whole-genome sequencing (WXS/WGS) data was built from five separate melanoma studies. Two of these studies (Hugo et al.[71]: SRA: SRP067938, SRP090294; Van Allen et al.[72]: dbGaP: phs000452.v2.p1.c2) focused on melanoma response to immune-checkpoint inhibitors (ICPIs). Two of these studies (melanoma exome sequencing: dbGaP: phs000933.v2.p1.c1;[73,74] the genetic and transcriptomic evolution of melanoma: dbGaP: phs001550.v2.p1.c1[75]) were broader melanoma studies focusing on the genetic basis of sun-exposed melanoma and melanoma evolution. The final study consisted of individuals from the UKBB with WXS/WGS with ICD10 codes: C433, C434,

C436, and C437. We did not have ancestry information for the first four studies, and for the UKBB individuals, the majority were of European ancestry (n = 236/239). Validation set individuals were also filtered by age to exclude individuals under 20 years old. MHC-I genotypes for the validation set were called with HLA-HD.[70] In total, there were 586 individuals in our validation set for which we were able to infer MHC-I autoimmune (AI) allele carrier status. Out of these 586, we were able to obtain fully resolved HLA types (i.e., *HLA-A*, *HLA-B*, and *HLA-C* genotypes) for 559 individuals. Discovery and validation sets are described in Table S1.

We used cases from the Melanostrum Consortium (n = 3,001) to evaluate PRS generalizability from absolute risk (i.e., cases vs. controls) to relative risk (i.e., age-specific effects). These cases were collected from countries of European ancestry including Italy (n = 1,239), Spain (n = 1,024), Greece (n = 716), and Cyprus (n = 22). Genotypes were available at 204 risk SNPs used in the development of the PRS by Gu et al.[76] Cases from this dataset were filtered to only include individuals $\geq$ 20 years in age.

We used the Million Veteran Program (MVP; n = 187,292) to assess the generalizability of our findings to melanoma incidence. Both controls (n = 171,878) and cases (n = 15,414) were constructed from individuals both $\geq$20 years of age at the time of last follow-up and of European descent as determined by HARE (harmonized ancestry and race/ethnicity).[77] We did this to match the predominantly European composition of the discovery and validation datasets and to maximize the comparability of effect sizes relative to PRS SNPs measured in the Melanostrum study (PRS Implementation). Controls excluded individuals with non-melanoma cancer diagnoses as determined by PheCode.[78] MHC-I alleles for the MVP were called with HIBAG[79] with the multi-ethnic IKMB and Axiom UK Biobank array models.[80] MVP 1.0 Axiom array design and genotype quality control details are described elsewhere.[81]

To identify putative conserved antigens, we leveraged RNA sequencing (RNA-seq) from a set of healthy melanocytes (dbGaP: phs001500.v1.p1) derived from newborn foreskins (n = 106) and version 7 of the Genotype-Tissue Expression (GTEx) project (dbGaP: phs000424.v7.p2).

Quantifying immune infiltration

To quantify immune infiltrates, we ran CIBERSORTx[82] with the LM22 signature matrix[83] on the bulk RNA-seq available for TCGA melanomas, which we downloaded from the TCGA GDC portal. RNA-seq reads were realigned and quantified by means of Sailfish[84] (with default parameters). Infiltration analyses were confined to primary tumors (n = 103) where the tumor immune microenvironment would be most relevant to the risk of developing melanoma. The effect of AI alleles on $CD8^+$ T cell infiltrates was modeled by multivariable regression, first including raw tumor mutation burden (TMB) as a covariate, and later including neoantigen burden at weak and strong cutoffs and *BRAF* (MIM: 164757) c.1799T>A (p.Val600Glu) mutation status.

Identifying driver mutations

We downloaded whole-exome sequencing (WXS)-based mutation calls from the TCGA GDC portal from four different mutation callers: VarScan,[85] MuSE,[86] MuTect,[87] and SomaticSniper.[88] RNA variant allelic fraction (VAF) was obtained with bam-readcount. From these mutation calls we focused on single-nucleotide variants, which dominate the landscape of melanoma and account for the majority of driver events.[89–91] A mutation was considered to be a potential driver if it (1) altered protein sequence, (2) was found in both the DNA and RNA in at least one individual, and (3) had a median DNA and RNA VAF percentile less than or equal to 40%. DNA mutations were only considered at an affected-individual-specific level if they were called by at least two of the mutation callers mentioned above. In total 51,062 mutations satisfied these criteria.

We further filtered the list of putative drivers on the basis of recurrence. Specifically, if a specific mutation was detected in four or more different tumors, we categorized it as a likely driver. In total 109 mutations satisfied this criterion (0.215% of the 51,062 candidate mutations). For those mutations that failed to reach this recurrence, we calculated mutation-specific contributions to melanoma pathogenicity by using scores from a melanoma-specific CHASM classifier.[92,93] Mutations with a CHASM score greater than or equal to 0.9 were deemed to be likely melanoma drivers; 106 mutations satisfied this criterion (0.206% of the 51,062 candidate mutations). Combining these recurrent and predicted driver singleton mutations yielded a final set of 215 melanoma drivers.

Identification and differential expression of conserved antigens

GTEx V7 contains 11,688 RNA-seq samples from 714 donors across 53 tissue types and was aligned with STAR v2.4.2a to GENCODE v19 and quantified with RNA-SeQC v1.1.8. RNA-seq reads from healthy melanocytes were aligned with STAR v2.5.0b to GENCODE v19 and quantified with RSEM (RNA-seq by expectation maximization) v1.2.31. After quantification, both melanocyte and GTEx datasets were filtered such that genes with $\leq$0.5 RSEM or with counts <6 in >93% of samples were removed. Additionally, ribosomal RNA, Y chromosomal, and histone genes were removed. Ribosomal and histone mRNA are not polyadenylated. Notably, the melanocyte dataset is exclusively male, while GTEx is not, which would potentially lead to false identification of Y chromosomal genes as stably expressed downstream.

Across healthy melanocytes and each tissue type in GTEx, genes were scored as stably expressed genes (SEGs) with the output from the scoring method described in scMerge.[94] Briefly, the method first fits the expression of each gene from each tissue sample to a gamma-Gaussian mixture. For the expression of a gene x_i, the gamma component corresponds to samples with low expression and the Gaussian component corresponds to samples with high expression. This mixture has the joint density function

$$f(x_i;\alpha_i,\beta_i,\mu_i,\sigma_i,\lambda_i) = \lambda_i \times Gamma(\alpha_i,\beta_i) + (1-\lambda_i) \times N(\mu_i,\sigma_i)$$

where α_i and β_i are the shape and rate parameters of the gamma component, μ_i and σ_i are the mean and standard deviation of the Gaussian component, and the mixing proportion λ_i is bounded by [0,1]. The SEG scoring method also takes into account the proportion of zeros in each gene ω_i for each tissue. Each gene is then scored by the percentile ranks of its mixing proportion λ_i, coefficient of variation (CV) σ_i/μ_i, and proportion of zeros ω_i such that the average percentile rank across all three metrics is minimal. The highest scoring genes have lower mixing proportions, CVs, and proportion of zeros.

Using the aforementioned method, we fit each gene from every tissue to a gamma-Gaussian distribution by using scMerge v1.6.0 and gave it a score from 0 to 1 for stable expression. Then, we removed the set of genes across all GTEx tissues that had scores $>$ 0.69 from the set of genes in melanocytes that had

scores > 0.69. The threshold of 0.69 was chosen on the basis of the observation that the scores of canonical melanocyte genes were, with the exception of *DCT/TYRP2* (MIM: 191275), all ~0.7 and by visual inspection of the distributions of scores across tissues (Figure S1). This process yielded four canonical melanocyte genes (*PMEL* [MIM: 155550], *MLANA* [MIM: 605513], *TYRP1* [MIM: 115501], and *TYR* [MIM: 606933]) and 48 additional protein-coding genes for downstream analysis. The 48 non-canonical melanocyte genes were also passed to PANTHER[95] for Reactome pathway[96] overrepresentation analysis with Fisher's exact test, which identified several genes as members of the folate metabolism pathway (false discovery rate [FDR] 0.0288).

To determine differential expression of conserved antigens in melanoma relative to melanocytes, we applied the same expression quantification pipeline and gene-filtering steps to both healthy melanocytes and TCGA SKCM samples. Specifically, we quantified HLA-allele specific expression by using HLApers v1.0[97] and the Kallisto v0.44.0[98] pipeline for HLApers. Reads were aligned to GENCODE v30[99] and IMGT HLA v3.41.0.[100] After quantification, both datasets were filtered in the same way as the conserved antigen identification pipeline described above. Additionally, 11 samples with missing age of diagnosis were removed from the TCGA SKCM set. We then performed a differential expression analysis with DESeq2 v1.30.1[101] conditioned on ancestry. However, several potentially relevant covariates were also incompatible across these two datasets. Namely, age, sex, tumor type (primary or metastatic), tumor purity,[102] melanocytic plasticity score,[103] and TIDE score.[104] As a result of these discrepant covariates, we also checked that no covariates were associated with significant differential expression for any conserved antigens. The only gene subject to differential expression was *MAGEA10* (MIM: 300343) with a -1.3 ± 0.4 log fold change (LFC) in primary vs. metastatic melanoma. This is substantially less than the +8 LFC of *MAGEA10* observed in melanocytes vs. melanoma (investigating mechanisms of MHC-I autoimmune allele protection).

Predicting binding affinities

MHC-I allele-binding affinities were computed across the available 2,915 unique MHC-I alleles for both driver mutations and conserved antigens. Since driver mutations altered protein sequence, we evaluated MHC-I alleles' ability to present neoepitopes by generating all unique 8- to 11-mers found in a mutation relative to the wild-type (corresponding to the set of novel peptides an MHC-I allele can present to the immune system). To circumvent cross-allele and cross-peptide variabilities that are inherent in predicted IC50 comparisons, we used percentile ranks relative to a random set of peptides provided by NetMHCpan-4.1[105] to approximate binding affinity for every MHC-I allele peptide pair. These percentile rank scores correspond to how strongly an allele binds a particular peptide relative to a set of random natural peptides. From peptide-level rank scores, MHC-I mutation-specific binding affinities were assigned according to the best rank score, the minimum allele-specific rank score across all unique 8- to 11-mers for a mutation.

For conserved antigens, we partitioned proteins into their entire set of 8- to 11-mers across the full length of the protein. MHC-I allele peptide pair percentile rank scores were again generated with NetMHCpan-4.1. Several metrics were derived from the percentile rank scores for downstream analyses. For broad and gene-level comparisons, we defined an allele-specific conserved antigen repertoire as the set of 8- to 11-mers presented at or below a given percentile rank. For position-wise presentability (Figure S2), we used the best percentile rank of all overlapping peptides at each position along a protein. We also defined the fraction of a gene presentable (FGP) by a given HLA allele as the fraction of 8- to 11-mers from the encoded protein predicted to bind at a percentile rank less than 0.5.

HLA population allele representations

To compare *HLA-B* and *HLA-C* autoimmune (AI) alleles to common non-AI alleles, defined as those alleles with a population frequency ≥ 1% as given by the National Marrow Donor Program (NMDP; 19 common *HLA-B* alleles, 13 common *HLA-C* alleles), we established maximum and minimum population allele representations. For driver neoantigens, we assigned a maximum population allele representation to have coverage at each rank equating to the coverage of the best presenting common allele at that rank. Similarly, we assigned a minimum population allele representation to have coverage at each rank equating to the coverage of the worst presenting common allele at the rank. For maximum and minimum population allele representations in conserved antigens (CAs), the metric of assignment was the fraction of CA peptides bound as opposed to coverage, as was consistent with CA analyses.

PRS implementation

We implemented the melanoma PRS developed by Gu et al.,[76] comprising 204 SNPs detected in subjects from Northern Europe, Australia, and the United States and validated in subjects from Southern Europe. For the discovery set, we were able to extract 190 of the 204 risk SNP genotypes by using PLINK.[106] For the validation set, datasets lacking sufficient SNP data for PRS construction were excluded, leaving 239 individuals for which 201 of the 204 risk variants were extracted with PLINK2.[107] For the Melanostrum Consortium ($n = 3{,}001$), all 204 risk SNPs were extracted. We compared risk SNP minor allele frequencies (MAFs) across datasets to ensure no significant differences (Figure S3). For the MVP, we were able to extract 202 of the 204 risk variants (rs2025016 and rs6833655 were missing) by using PLINK2.[107] The final PRS for each dataset was generated as a weighted sum across extracted risk SNPs, ensuring SNPs were oriented to the correct allele, in each dataset in accordance with the optimal melanoma risk model by Gu et al.[76]

Multistage carcinogenesis model for melanoma

Dating back to the 1950's Armitage-Doll model[108] of cancer incidence and those created soon after by Knudsen and Moolgavkar[109] and others, multistage models of cancer are among the most developed mathematical methods for defining carcinogenesis and determining timescales of tumor formation in human populations.[110–114] These models assume evolutionary stages from normal cells to development of clinically detected symptomatic cancers. These stages typically include intermediate premalignant and preclinical malignant stages that represent field cancerization dynamics of stochastically growing and shrinking clonal populations in a tissue. These models can be described mathematically as stochastic multi-type branching processes with probabilities of events occurring with certain rates (Figure S4A). By calculating an age-dependent hazard function for cancer incidence via solutions to equations from the probability generating functions starting from birth, we can calibrate these models to fit hazard rates derived from cancer incidence registry data such as Surveillance, Epidemiology, and End Results (SEER) in the US.[115] Importantly,

this modeling framework provides a link between cell-level dynamics and population-level incidence data so that we can estimate parameters governing clonal growth, dwell times, and mutational "hits" in at-risk individuals.

In previous work, we found that the "two-stage" model (two "hits" for development of a first malignant cell) shown in Figure S4 is closely approximated by a model that includes an effective malignant transformation rate and a characteristic lag-time or "sojourn" time between malignant transformation and clinical detection (see Luebeck et al. 2013 for mathematical details[116]). Here, we created a two-stage model for melanoma incidence that adjusts for birth trends, similar to methods used previously in esophageal squamous cell carcinoma (ESCC [MIM: 133239]).[117] In this way, our models capture trends for both age and birth (and thus calendar period) to enable robust estimation via Markov chain Monte Carlo (MCMC) simulation of cell-level parameters for tumor evolution by sex and race/ethnicity (in the absence of genetic ancestry; see Figure S4B for examples of model fits). We obtained estimates and 95% confidence intervals for tumor sojourn times in males and females via MCMC posterior estimates for the lag-time parameter. Chains were run for 100,000 cycles with a 4,000 cycle burn-in and checked for convergence. All code for hazard function calculation and parameter estimation was written in Fortran. The ICD-O-3 codes used for extraction of SEER data melanoma, all races combined, from SEER*Stat include 8720/3, 8721/3, 8722/3, 8723/3, 8726/3, 8727/3, 8728/3, 8730/3, 8740/3, 8741/3, 8742/3, 8743/3, 8744/3, 8745/3, 8746/3, 8761/3, 8770/3, 8771/3, 8772/3, 8773/3, 8774/3, 8780/3, 8790/3.

Personalizing sojourn time estimates from clock-like mutational signatures

To personalize sojourn time estimates, we combined the sex-specific estimates returned by our two-stage model for melanoma incidence (multistage carcinogenesis model for melanoma) with tumor-specific genetic marks in TCGA. Specifically, we quantified the per-affected-individual UV-corrected SBS1 signature by counting CpG>TpG mutations after excluding CpCpG and TpCpG mutations. This adjusted SBS1 has previously been used as a molecular clock for melanoma.[118,119] After extracting UV-corrected SBS1 CpG>TpG mutations in TCGA ($n = 397/451$), we fit a linear model predicting age of diagnosis from these clock-like mutational signatures. Personalized predicted age of onset was then estimated by

$$\begin{aligned}\text{Age of Onset} &= \text{Age of Diagnosis}\\ &- (\text{Sex Specific Tumor Sojourn Time Estimate}\\ &+ (\text{C} > \text{T Burden} - \mathit{median}(\text{C} > \text{T Burden}))\cdot\beta_{\text{C}>\text{T mutation}})\end{aligned}$$

where sex-specific tumor sojourn time estimate equates to the outputs of our two-stage melanoma incidence model, C>T burden equates to the number of UV-corrected SBS1 CpG>TpG mutations one carries, and $\beta_{\text{C>T mutation}}$ equates to the coefficient of the linear model predicting age of diagnosis from UV-corrected SBS1 CpG>TpG mutations. For those individuals for which UV-corrected SBS1 CpG>TpG mutations could not be inferred, we substituted the median UV-mutation-spectrum-corrected CpG>TpG mutation burden as their C>T burden, leading to a shift equivalent to the aforementioned sex-specific sojourn time estimates. Note here that personalized sojourn time estimates equate to the shift (i.e., subtraction) from age of diagnosis.

To assess the validity of these personalized sojourn time estimates, we ensured these estimates fell within expected ranges. Specifically, we compared our personalized sojourn time estimates to the expected time of whole-genome duplication (WGD) to melanoma diagnosis as reported by Gerstung et al.[118] We calculated WGD status for TCGA individuals from ploidy, which was computed as the length weighted sum of genomic segments with constant total copy number. For more details, we direct the reader to the work by Steele et al.[120] from which these values were obtained.

Calculating HLA loss of heterozygosity

Inference of HLA loss of heterozygosity (LOH) was determined by means of the computational tool LOHHLA.[52] As input, we provided normal and tumor DNA BAM files, consensus HLA calls (obtained from POLYSOLVER and HLA-HD),[69,70] and purity and ploidy estimates as returned by Sequenza.[121] Upon completion of LOHHLA, HLA alleles were classified as lost if they exhibited a copy number < 0.5, in accordance with LOHHLA documentation. We also employed an additional quality control step ensuring all alleles classified as lost had a reported p value < 0.05. Across SKCMs in the TCGA, we observed 90 incidences of HLA LOH across 56 individuals. For the seven AI alleles investigated in this study, we observed 16 incidences of AI HLA LOH across 12 AI-allele carriers.

Statistical analyses

All boxplot statistical tests comparing age of diagnosis effects between groups were assessed with the default Mann-Whitney U statistical test. We conducted leave-one-out analysis by narrowing the AI allele set into all seven unique sets of six AI alleles and stratifying individuals accordingly. Performing leave-one-out analysis by dropping all carriers of each allele yielded similar results, and AI status was significantly associated with a later age of diagnosis in each holdout set. t tests were used to compare PRS distributions across AI-allele status in both discovery and validation sets. We used Fisher's exact tests to evaluate associations between HLA-proximal PRS SNPs and MHC-I AI-allele carrier status. These statistical tests were all implemented via the default scipy.stats Python package. Regression analyses were modeled with ordinary least squares linear models through the statsmodels.formula.api Python package.[122] Effect sizes for AI alleles and PRS SNPs in the TCGA were calculated with Cliff's D. Effect sizes for AI alleles and PRS SNPs in the case-control MVP were reported as odds ratios calculated from logistic regression through the statsmodels.api Python package. Wherever multiple hypotheses were tested, p values were corrected by the Benjamini-Hochberg procedure, implemented through the statsmodels.stats.multitest package in Python.

Results

MHC-I autoimmune alleles associate with a later age of diagnosis in melanoma

To investigate the relationship between autoimmunity and melanoma development, we sought evidence that MHC-I AI alleles could provide protection from melanoma. We defined a set of eight MHC-I alleles on the basis of documented links to skin AI conditions. This set included three alleles linked to psoriasis (*HLA-B*27:05*, *HLA-B*57:01*, *HLA-C*12:03*),[123–127] two alleles linked to vitiligo (*HLA-A*02:01*, *HLA-B*13:02*),[128–133] and one allele linked to both conditions (*HLA-C*06:02*).[132–135] We also included two alleles (*HLA-B*39:06*, *HLA-B*51:01*) with postulated

psoriasis associations[123,136] but strong associations with other AI conditions, specifically type 1 diabetes (MIM: 222100)[137–139] and Behcet's disease (MIM: 109650),[140] respectively (Table S2). Among these, all but HLA-A*02:01 are associated with a narrow specificity for antigen[141,142] and have a population allele frequency < 10% (Table S3).

Using individuals with SKCMs from the TCGA as our discovery set (Table S1), we called MHC-I genotypes by using two exome-based methods: POLYSOLVER and HLA-HD (Material and Methods: Datasets).[69,70] AI-allele frequencies in the discovery set were present at the population distributions as reported by the NMDP[143] (Table S3). Individuals under 20 years of age were also excluded from further analysis given their increased likelihood of harboring rare germline predisposing risk variants.

To evaluate the effect of carrying an AI allele, we partitioned the discovery set into two groups: those with at least one AI allele and those lacking any of these alleles. We first assessed the potential for AI-allele carrier status to be confounded by sex or UV exposure, two factors that influence melanoma incidence. Males have a well-documented higher risk of developing melanoma,[144,145] however, we did not observe any sex-specific age differences (Figure S5A, $p = 0.247$). UV-associated mutational signatures correlated strongly with overall tumor mutation burden in the discovery set (Pearson $R = 0.984$; Figure S5B). There were also no significant differences in mutation burden or UV-associated mutational signatures between AI-allele carriers and non-carriers (Figures S5B–S5D; A*02:01 included: $p_{mutation} = 0.365$, $p_{UV} = 0.186$; A*02:01 excluded: $p_{mutation} = 0.352$, $p_{UV} = 0.415$).

We hypothesized that among individuals diagnosed with melanoma, a protective effect would manifest as delayed disease onset relative to individuals without these alleles. As HLA-A*02:01 differs from the other seven AI alleles in antigen presentation properties[141,142] and population frequency and may promote higher expression of the immune suppressive checkpoint TIM-3 (MIM: 606652) by T cells relative to the other AI alleles,[146] we considered AI-carrier status both excluding and including HLA-A*02:01. In the discovery set, AI-allele carrier status excluding *HLA-A*02:01* was significantly associated with a median later age of melanoma diagnosis of 5 years (Figure 1A, $p = 0.002$). Upon including *HLA-A*02:01*, this association was lost (Figure S5E, $p = 0.316$). Evaluation of T cell infiltration levels estimated by CIBERSORTx[82] (quantifying immune infiltration) indicated that primary tumors carrying AI alleles had significantly lower levels of regulatory T cells (Figure 1B, $p = 0.048$). However, tumors in *HLA-A*02:01* carriers showed no reduction in regulatory T cells (Figures S5F–S5G). For subsequent analyses, we therefore defined AI-allele carrier status on the basis of the seven non-*HLA-A*02:01* AI alleles; individuals carrying only *HLA-A*02:01* were considered non-carriers.

To further probe allele-specific contributions, we performed a leave-one-out analysis, evaluating AI age effects across all seven single allele exceptions. Regardless of which allele was held out, we consistently observed a significant relationship between AI status and age of diagnosis (Figure S6), with a 5-year-later median age of melanoma diagnosis (median age without AI = 57; median age with AI = 62). We did not observe significant differences at the level of HLA supertype, suggesting that the effects are specific to individual AI alleles (Figure S7); the largest observed effect was for the B44 supertype, to which none of the AI alleles belong, however it was not significant after multiple testing correction ($p_{adj} = 0.199$, median earlier age of diagnosis difference = 4 years).

For the seven-allele definition of carrier status, later age of diagnosis was conserved across sex with a median age difference of 6 years in males and 4 years in females (Figure 1C, $p_{male} = 0.011$, $p_{female} = 0.033$). We used an ordinary least squares (OLS) regression to model the effect of carrying an AI allele on age at diagnosis with tumor thickness, sex, and mutation burden as covariates and these findings remained significant with a predicted 4.76 delayed years to melanoma diagnosis (Figure 1D, $p = 0.003$). As tumor thickness was only available for a subset of tumors ($n = 347$), we also evaluated the effect of AI-allele carrier status with primary vs. metastatic disease, sex, and mutation burden as covariates ($n = 416$) and sex and mutation burden only as covariates ($n = 451$). Both analyses yielded similar results, with a predicted 3.62 (Figure S8A, $p = 0.015$) and 4.07 (Figure S8B, $p = 0.005$) delayed years to melanoma diagnosis, respectively. We further evaluated whether carrying multiple AI alleles had an additive effect. When we used a linear model to predict age of diagnosis as a function of an individual's total number of AI alleles, the discovery set showed a significant 2.6 delayed years to melanoma diagnosis per AI allele (Figure 1E, $p = 0.002$; confidence interval [CI] = [0.935, 4.262]).

We also observed that the number of AI alleles significantly associated with increased $CD8^+$ T cell infiltration in primary tumors (Figure 1F). In a linear model predicting $CD8^+$ T cell infiltration as a function of an individual's total number of AI alleles, the per MHC-I AI allele $CD8^+$ T cell infiltrate increase was 2.7% ($p = 0.040$; CI = [0.127%, 5.282%]). When we included tumor mutation burden as a covariate, the effect of AI alleles on increased $CD8^+$ T cell infiltration remained significant, yielding a 2.65% increase per MHC-I AI allele carried ($p = 0.045$; CI = [0.056%, 5.253%]). Together, these analyses suggest that the delayed diagnosis associated with AI alleles could be driven by a more robust $CD8^+$ T cell response less impeded by regulatory T cell activity.

To confirm these findings, we investigated the age of melanoma diagnosis and AI allele presence in an independent validation set of 586 individuals diagnosed with cutaneous melanoma, compiled from four published dbGaP studies[71–75] and the UK Biobank. Although AI-allele frequencies once again matched closely with the expected distribution (Table S3), we observed notable differences in the distribution of sex and age (Table S1), especially for the UK Biobank. Whereas in TCGA the sex and age

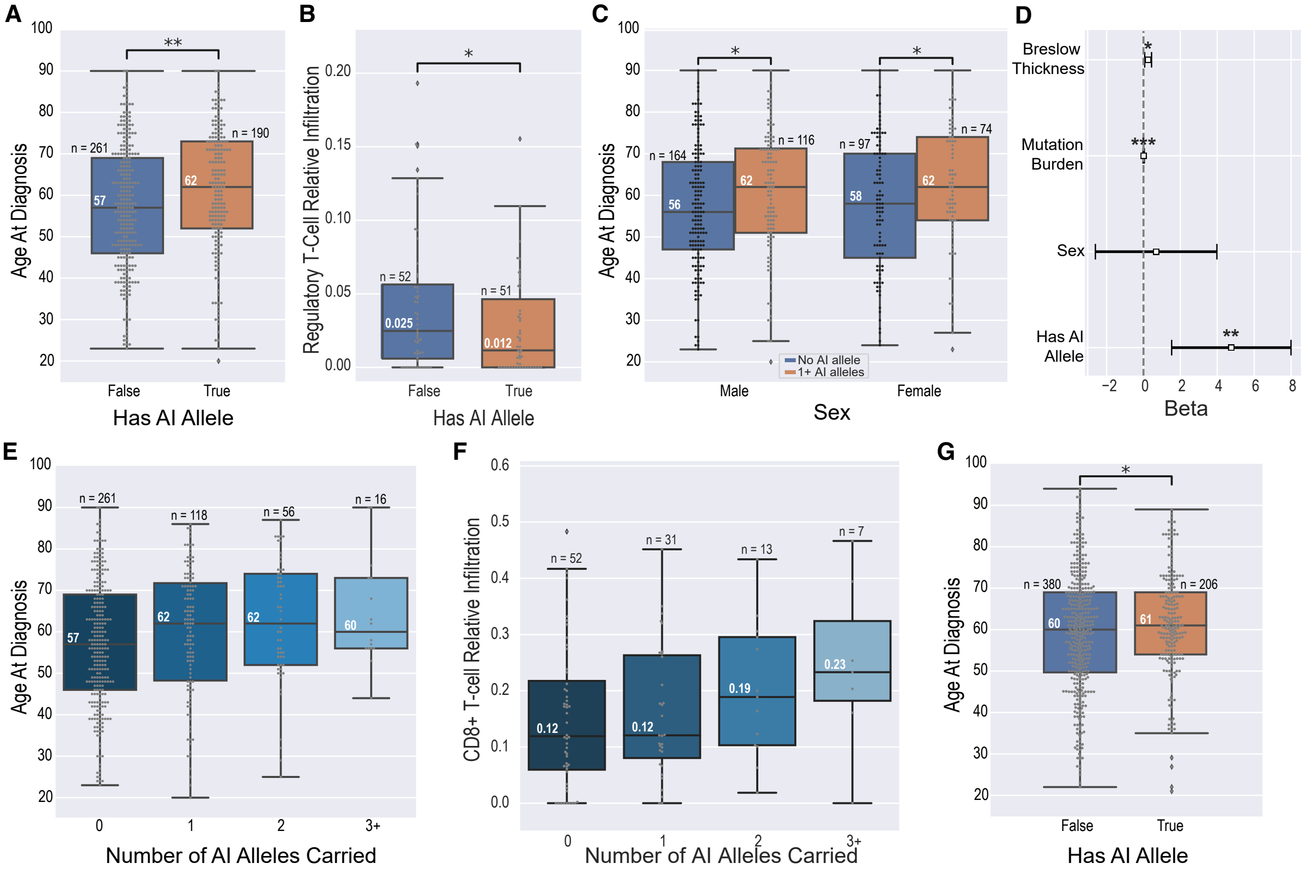

Figure 1. Effect of MHC-I AI alleles on melanoma age at diagnosis and immune infiltration
(A) TCGA: having at least one MHC-I linked AI allele is associated with a significant median later age of melanoma diagnosis of 5 years ($p = 0.002$).
(B) TCGA: carriers of at least one MHC-I AI allele exhibit a significant reduction in regulatory T cells in primary melanoma tumors ($p = 0.048$).
(C) TCGA: MHC-I-linked AI allele age of melanoma diagnosis effect is conserved across sex ($p_{male} = 0.011$, $p_{female} = 0.033$).
(D) TCGA: effect sizes and significance of coefficients in a multivariable model fitted to age at diagnosis. Having at least one MHC-I-linked AI allele associated significantly with a 4.76-year delay to melanoma diagnosis after controlling for tumor thickness, sex, and mutation burden ($p_{autoimmune} = 0.003$, CI = [1.581, 7.943]).
(E) TCGA: age of diagnosis significantly increases with an individual's total number of MHC-I autoimmune alleles ($p = 0.002$).
(F) TCGA: infiltrating CD8$^+$ T cell levels increase with the number of AI alleles carried ($p = 0.040$).
(G) Validation: having at least one MHC-I linked AI allele is associated with a significant median later age of melanoma diagnosis of 1.0 year ($p = 0.044$).

distributions of melanoma diagnosis closely matched the rates reported by SEER (an M/F ratio of 1.637 in TCGA versus an M/F ratio of 1.624 in SEER), females were overrepresented in the UK Biobank (an M/F ratio of 0.498) and female sex across the entire validation set was significantly associated with an earlier age of melanoma diagnosis independent of AI status (Figure S9A; $p = 0.003$, median earlier age of diagnosis difference = 3 years). This suggests an intrinsic selection bias within the studies from which our validation set was compiled. If these biases relate to unmeasured environmental risk factors, they could mask the contribution of genetic risk factors. Despite this, we again observed that having at least one AI-linked MHC-I allele was significantly associated with a later age of diagnosis (Figure 1G, $p = 0.044$; median age separation = 1 year). While the direction of this effect was conserved across males with a median age difference of 1 year, we did not observe any significant differences in females (Figure S9B; $p_{male} = 0.055$, $p_{female} = 0.236$).

As most validation samples lacked tumor sequencing data, it was not possible to estimate UV exposure. Thus, our regression analysis was limited to the presence of an AI allele and sex. Validation individuals with at least one AI allele had a predicted 2.10 delayed years to melanoma diagnosis relative to those without any of these alleles ($p = 0.082$; CI = [−0.265, 4.457]). By comparison, the discovery set showed a predicted 4.0 delayed years to melanoma diagnosis when only AI status and sex are considered ($p = 0.006$; CI = [1.154, 6.850]). In the validation group individuals with fully resolved HLA types ($n = 559/586$), we observed a predicted 0.727-year delay to melanoma diagnosis per AI allele, although these results did

not reach statistical significance ($p = 0.328$; CI = [−0.731, 2.184]).

Finally, we attempted to identify other HLA alleles associated with age at diagnosis by comparing individuals with a particular allele to all individuals without that allele. While none of the associations were significant after multiple hypothesis testing correction, we did note that *HLA-B*27:02* showed an earlier median age of diagnosis in both datasets (Figure S10; SKCM-TCGA: 10 years earlier, $n_{\text{Has } HLA\text{-}B^*27:02} = 15$, $p = 0.050$, $p_{adj} = 0.483$; validation set: 11 years earlier, $n_{\text{Has } HLA\text{-}B^*27:02} = 3$, $p = 0.033$, $p_{adj} = 0.388$). However, given the limited dataset sizes with sequencing data, coupled with the highly polymorphic nature of the HLA, this analysis is underpowered and may warrant further investigation in larger datasets.

MHC-I autoimmune alleles modify melanoma risk

PRSs use information about genetic risk factors to predict individual disease risk. We evaluated the utility of incorporating MHC-I AI-allele carrier status into a risk-scoring framework in the context of the PRS developed by Gu et al.[76] (PRS implementation). This PRS comprises 204 SNPs, of which 16 are found on chromosome 6, although none fall within the HLA class I region (Figure 2A). Although the HLA class I genes are not among the genes associated with each SNP as reported by Gu et al.,[76] we assessed whether HLA-proximal PRS SNPs (i.e., those SNPs within 3 Mb of the HLA-coding region) associated with MHC-I AI-allele carrier status across our discovery set but observed no significant relationship ($p_{adj} = 0.235$).

As the PRS was developed to stratify cases from controls, we ensured that it could be generalized to capture age-specific effects in a case-only setting through regression analysis. We observed that higher PRS associated significantly with earlier age of diagnosis in the TCGA (discovery: $p = 0.002$, beta = −7.499, CI = [−12.141, −2.857]) but not in the validation set (validation: $p = 0.710$, beta = 0.7551, CI = [−3.237, 4.747]), consistent with unmeasured confounders masking genetic risk factors. Given the disparity in PRS generalization, we evaluated the PRS age stratification in melanoma cases from the Melanostrum Consortium (n = 3,001), the original PRS validation set.[76] Here, we observed that higher risk scores were again biased toward an earlier age of diagnosis ($p = 0.055$, beta = −1.780, CI = [−3.598, 0.038]). While the MAF of the PRS SNPs generally correlated well across TCGA, Melanostrum, and the validation set, we did observe certain SNPs with an MAF difference ≥ 0.1 across datasets. Four SNPs exhibited this difference between our discovery set and Melanostrum (rs1464510, rs187989493, rs7041168, and rs7164220). Between our validation set and Melanostrum, seven SNPs exhibited this MAF gap (rs187989493, rs1393350, rs7164220, rs13338146, rs12919293, rs75570604, and rs2092180), including the maximum PRS effect SNP (rs75570604). Finally, between our discovery and validation sets, one SNP exhibited an MAF difference ≥ 0.1 (rs1464510) (Figure S3). These discrepancies may explain the varying performance of PRSs in age stratification across datasets.

We next evaluated the relationship between PRSs and age at diagnosis in AI-allele carriers versus non-carriers. As expected, PRS distributions did not differ between those with and without MHC-I AI alleles in either dataset (Figure S11; $p_{disc} = 0.999$, $p_{val} = 0.901$). Increases in PRS showed a greater negative effect on age at diagnosis in those with an AI allele relative to those without one in the discovery set (Figure 2B). However, interaction effects from a linear model between PRS and AI MHC-I allele genotype in the discovery set, while large in magnitude, did not reach significance ($p = 0.055$, Beta = −9.124, CI = [−18.432, 0.183]) and did not reproduce in the validation set (Figure 2C).

We also sought to assess whether AI carrier status was associated with melanoma incidence. In a large case-control dataset composed of individuals from the MVP[148] (n = 187,292; Datasets), AI MHC-I carriers were less likely to have a melanoma diagnosis (odds ratio [OR] = 0.962, $p = 0.024$), as would be expected if MHC-I AI alleles conferred a protective effect against melanomagenesis. Each additional AI allele carried further decreased the likelihood of melanoma (beta = −0.031, $p = 0.003$). To quantify the magnitude of AI MHC-I allele carrier status relative to individual PRS SNPs, we compared effect sizes in both the discovery set and the MVP. PRS stratification in the MVP yielded an area under the receiver operating characteristic curve (AUC) of 0.61, in line with, but less than, the reported 0.644 AUC in the Melanostrum Consortium[76] (PRS implementation). Compared to the five largest PRS weight SNPs in both directions (i.e., melanoma predisposing and protective), MHC-I AI-allele status exhibited the fourth largest effect size by magnitude and second largest positive effect size in the discovery set (Figure 2D, Cliff's D = 0.165). In the MVP, the effect of having an MHC-I linked AI allele was more modest, with the smallest OR by magnitude relative to the ten largest weight PRS SNPs (Figure 2E, OR = 0.962, CI = [0.930, 0.995]).

We next evaluated whether these trends extended to non-MHC AI-risk SNPs. In total we examined 30 AI SNPs, including four with established vitiligo-melanoma associations either as the joint lead risk SNP for both conditions (rs1126809 and rs6059655) or in strong linkage disequilibrium with known cutaneous melanoma risk SNPs (rs72928038 and rs251464)[30] and one (rs9468925) that is associated with both psoriasis and vitiligo and falls in between *HLA-C/HLA-B*.[60] The remaining 25 AI SNPs are broadly associated with autoimmunity according to the criteria outlined by Chat et al.[38] (i.e., associated with at least three AI conditions and at least one of which surpassed a significance threshold of $p = 10^{-7}$) and were previously investigated in the context of improved immune-checkpoint inhibitor efficacy. While coefficients for the relationship between AI SNP genotype and age of melanoma diagnosis ranged from strongly protective (e.g., rs6679677; $beta_{Disc} = 2.775$; $beta_{Val} = 2.036$) to strongly predisposing (e.g.,

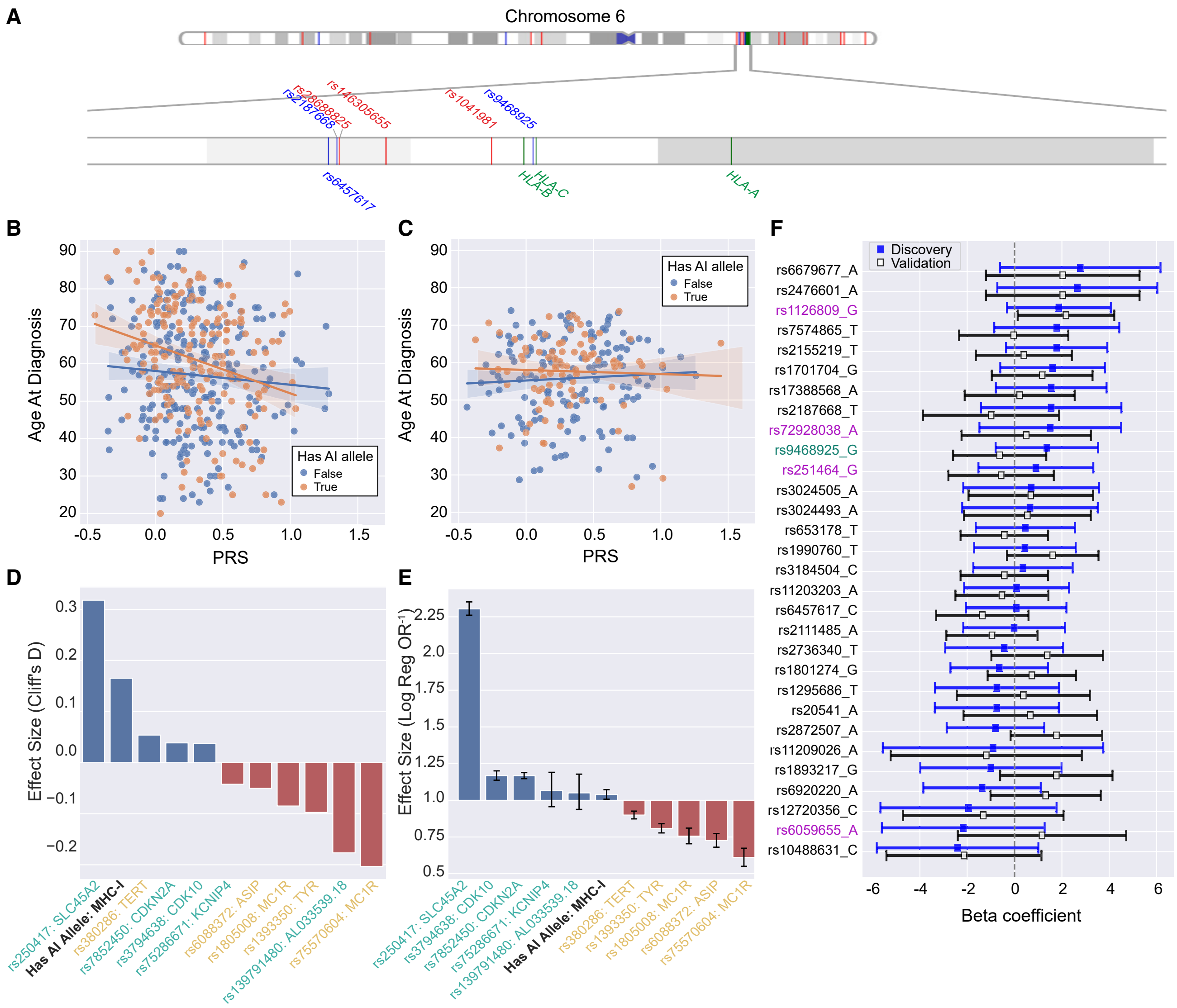

Figure 2. Contextualization and quantification of MHC-I AI alleles' effect on melanoma risk
(A) A PhenoGram[147] plot of chromosome 6 shows melanoma PRS SNPs (red) and AI-associated SNPs (blue) fall outside of the HLA-coding region (green). The distance from the closest PRS SNP (rs1041981) to the class I-coding region (*HLA-B*) is 215,819 bp. The closest AI SNP (rs9468925) falls in between *HLA-C/HLA-B*.
(B) TCGA: age of diagnosis as a function of PRS and AI allele presence. Individuals with AI MHC-I alleles show a steeper decrease in age of diagnosis as PRS increases ($p = 0.055$, beta = −9.124, CI = [−18.432, 0.183]).
(C) Validation: age of diagnosis as a function of PRS and AI allele presence. Individuals with AI MHC-I alleles again show a steeper decrease in age of diagnosis as PRS increases, but interaction effects were insignificant ($p = 0.499$, beta = −2.878, CI = [−11.254, 5.498]).
(D) TCGA: Cliff's D for having at least one MHC-I-linked AI allele, the five strongest PRS melanoma-predisposing SNPs mapped to their nearest gene (yellow), and the five strongest PRS melanoma-protective SNPs mapped to their nearest gene (turquoise).
(E) MVP: inverse odds ratios (1/OR) for MHC-I AI allele presence, the five strongest PRS melanoma-predisposing SNPs mapped to their nearest gene (yellow), and the five strongest PRS melanoma-protective SNPs mapped to their nearest gene (turquoise).
(F) AI SNP effect on age of diagnosis: AI SNPs showed varying associations with age of diagnosis ranging from strongly protective (high beta) to strongly predisposing (low beta). Overall these effects were highly variable and did not reach statistical significance after multiple-hypothesis correction. Joint vitiligo-melanoma-associated SNPs are marked in red, the lone *HLA-C/HLA-B* psoriasis and vitiligo SNP is marked in green, and the remaining 25 broad AI SNPs are marked in black. Error bars correspond to ± 2 standard deviations.

rs10488631; $beta_{Disc} = -2.407$; $beta_{Val} = -2.137$) across datasets, overall these effects exhibited large variability and were not significant after multiple-hypothesis testing (Figure 2F). In contrast to MHC-I AI alleles, including non-MHC-I AI SNPs as covariates with PRS did not improve prediction of age at diagnosis.

MHC-I autoimmune alleles associate with delayed onset of melanoma

To further evaluate whether delayed diagnosis observed for AI alleles corresponds to a delay in onset of melanomagenesis, we sought to impute individual-specific sojourn time, the time period from tumor initiation to diagnosis. Age at

onset can then be estimated by subtracting sojourn time from age at diagnosis. To obtain an individualized estimate of sojourn time, we combined an average sojourn time estimated from population statistics with a tumor-specific estimate based on the clock-like mutational signature, SBS1.[119]

To estimate the expected time between the initial transformed malignant cell (in a surviving malignant clone that escapes extinction) and clinical detection, we developed a cell-based stochastic branching process model for the development of independent premalignant clones (such as nevi) that can arise and clonally expand in normal skin epithelium (multistage carcinogenesis model for melanoma). Each cell in these clones has the propensity to transform to a malignant cell with a certain probability, the malignant clone population can expand in size or go extinct through a stochastic birth-death process, and clinical detection may occur with a size-based detection probability. Mathematically, the expectation of the lag-time variable, or the time between the founder cell of a persistent malignant clone and clinical detection, can be interpreted as the average "age" or sojourn time of the detected tumor.[116]

To obtain the estimated average, we analyzed SEER9 melanoma age- and group-specific incidence data from 1975 to 2018.[115] The hazard function from a "two-stage" model corresponded to the best fit to SEER incidence for both males and females (Figure S4). Adding additional stages to the model (i.e., more than two rate-limiting events or "hits" such as driver mutations required before malignant transformation) did not improve the fits. With estimated model parameters, we found that the expected tumor sojourn time in males was 8.35 years (MCMC 95% CI = [6.61, 9.73]) and similarly in females was 9.64 years (95% CI = [8.48, 10.66]). Previous studies have estimated melanoma doubling times that can be used to then calculate the corresponding tumor sojourn time. With a mean doubling time of 144 days,[149] the mean growth rate in an exponentially growing tumor is approximately 1.76 per year. Assuming malignant tumors are detected on average at 10^8 or 10^9 cells in size, this implies a melanoma sojourn time of 10.5 years and 11.8 years, respectively. This is in line with our above estimates, along with those found in a previous modeling study of melanoma doubling times (mean = 3.78 months).[150] Although estimates may vary on the basis of affected-individual-specific factors, our findings suggest that it takes approximately a decade on average for a melanoma to be detected after it is first initiated in an individual.

We next approximated an individual sojourn time on the basis of SBS1-associated mutation burden, such that the median sojourn time would correspond to the estimated baseline. Mutational signature SBS1 has been used previously as a molecular clock in melanoma, after first excluding the subset of mutations that overlap with the SBS7 signature of UV-driven mutagenesis, specifically mutations at CpCpG and TpCpG DNA contexts.[118] We compared the UV-corrected SBS1 CpG>TpG mutations to the uncorrected SBS1 in TCGA, confirming that it showed increased correlation with age of diagnosis (Figures S12A and S12B; $r_{C>T} = 0.230$, $r_{SBS1} = 0.112$; $\rho_{C>T} = 0.238$, $\rho_{SBS1} = 0.211$) and greatly reduced correlation with UV mutagenesis as quantified by SBS7 (Figures S12C and S12D; $r_{C>T} = 0.284$, $r_{SBS1} = 0.816$; $\rho_{C>T} = 0.353$, $\rho_{SBS1} = 0.434$). Fitting a linear model to predict age at diagnosis from UV-corrected SBS1 mutation burden, each mutation equated to approximately 0.85 years. We then estimated individualized sojourn times by matching the median UV-corrected SBS1 burden to the estimated average sex-specific time and adding or subtracting time according to how much larger or smaller the observed UV-corrected SBS1 burden was relative to the median (personalizing sojourn time estimates from clock-like mutational signatures).

To assess whether the estimates obtained are reasonable, we evaluated the sojourn time for individuals with ploidy profiles indicating WGD events.[118] Gerstung et al.[118] estimated that WGD occurs approximately 5.7 years prior to melanoma diagnosis with a range of 3.2 to 16 years. We reasoned that if our estimate of sojourn time based on UV-corrected SBS1 provided a good approximation, then the individualized sojourn time should exceed the timing of WGD in those tumors where it occurred. Estimating WGD events from ploidy information in TCGA, we found that sojourn estimates were significantly longer, and generally exceeded 5.7 years, for the subset of tumors with WGD (Figure S12E).

Subtracting the individual sojourn time estimates from age of diagnosis, we further partitioned our discovery set into PRS quintiles and stratified by AI carrier status. AI carrier status exhibited significant later predicted ages of onset for the lowest ($p = 0.011$) and second-highest risk quintiles ($p = 0.019$) (Figure S12F). Across quintiles, we observed the median predicted onset age ranged from 42 to 56, within the current recommended melanoma screening range. We did not observe longer sojourn times in AI carriers in TCGA, suggesting that AI alleles do not change the time from onset to diagnosis (Figure S12G). In the future, further customizing melanoma onset estimates with epigenetic marks has the potential to improve estimates of age at onset and inform optimal screening based on germline genetic risk factors.

Investigating mechanisms of MHC-I autoimmune allele protection

One possible explanation for a protective effect of selected HLA alleles would be a stronger affinity for neoantigens or tumor associated antigens that cannot easily be suppressed by melanomas. In psoriasis, melanocyte antigens such as ADAMTS-like protein 5 presented by HLA-C*06:02 (one of the seven AI alleles) can induce a targeted CD8$^+$ T cell response against melanocytes.[59] Similarly, in vitiligo, CD8$^+$ T cells target antigens from melanosomal proteins such as PMEL, MLANA/MART1, TYR, TYRP1, and DCT.[130,151,152] Interestingly melanoma-specific CD8$^+$ T cells appear to recognize peptides derived from these conserved melanocytic antigens more often than melanoma-specific

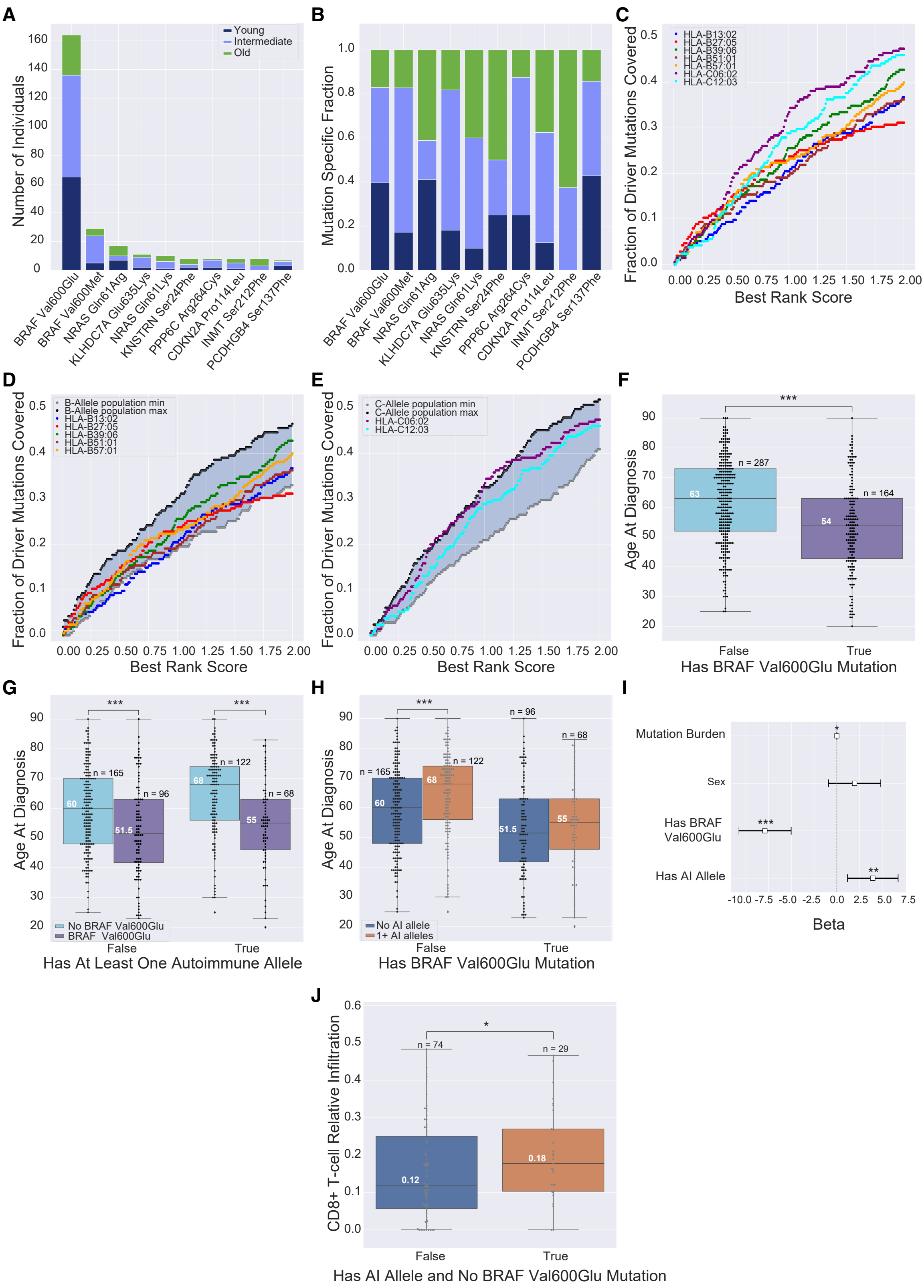

Figure 3. Identification and coverage of melanoma driver mutations including BRAF p.Val600Glu and its impact on age at diagnosis and immune infiltration

(A) Frequency of the ten most recurrent mutations across the TCGA by age group. Young (<50) and old (≥69) age groups correspond to the bottom and top 30% of individuals by age, respectively. The intermediate (50 ≤ x < 69) age group corresponds to the remaining 40% of individuals.

(B) Relative age group distribution of the ten most recurrent mutations across the TCGA.

(legend continued on next page)

antigens.[130,153–155] Given this, a protective effect in cancer could indicate that AI alleles mediate more effective immune surveillance against conserved cancer antigens (i.e., self-antigens overexpressed in tumors) or even against somatic mutations that promote tumor development.

We reasoned that a protective effect manifesting as delayed age of diagnosis would require more effective immune surveillance against early driver mutations or conserved melanocyte-specific antigens expressed by melanomas. For neoantigens, we identified a set of 215 mutations that exhibited joint high DNA and RNA variant allelic fraction coupled with either recurrence or driver likelihood as predicted by the CHASM algorithm[92,93] (identifying driver mutations). This included well-known driver mutations in *BRAF*, *NRAS* (MIM: 164790), and *CDKN2A* (MIM: 600160) (Figures 3A and 3B), and BRAF p.Val600Glu (V600E) was the most frequent mutation across the discovery set at 164 unique occurrences (Figure 3A). For conserved antigens, we included peptides derived from antigens associated with melanocytes[130,151] and melanoma, including the melanoma antigen gene (MAGE) family[156–162] and genes constitutively expressed in melanocytes (identification and differential expression of conserved antigens) resulting in 87 genes (Table S4). We evaluated whether MHC-I AI alleles could better expose driver neoantigens and conserved antigens for immune surveillance than 2,908 other common MHC-I alleles based on NetMHCPan-4.1 binding affinity scores for 8- to 11-mer peptides, using 0.5 and 2 percentile ranks as strong and weak binding cutoffs, respectively (predicting binding affinities).[163]

There was variability in the potential of MHC-I AI alleles to present peptides containing driver neoantigens in general: HLA-C*06:02 and HLA-C*12:03 presented the largest proportion of mutations across all affinities and HLA-B*27:05 presented the largest proportion at stronger affinities (Figure 3C). As compared to common alleles (HLA population allele representations), MHC-I AI alleles did not show particularly better binding affinity for neoantigens for either HLA-B or HLA-C alleles (Figures 3D and 3E), although HLA-C*06:02 had coverage close to and briefly exceeding the common allele maximum (Figure 3E). In general, maximum and minimum population B-alleles exhibited greater coverage than their corresponding C alleles at lower rank scores, but C alleles outperformed B alleles as the rank score exceeded 0.5 (Figure S13A).

There were also differences in which mutations generated neopeptides with the best specificity. While common and AI alleles on average exhibited specific mutation binding preferences (Figure S14) among the 215 drivers, there was no single mutation that was more effectively presented by all MHC-I AI alleles versus common alleles. One mutation, BRAF p.Val600Glu, showed significant age differences: mutation carriers were diagnosed with melanoma on average 9 years earlier than those without (Figure 3F, $p_{adj} = 7.58 \times 10^{-6}$). However, rather than observing a correlation between lack of AI-allele presence and having a BRAF p.Val600Glu mutation, BRAF p.Val600Glu status significantly shifted age of diagnosis earlier regardless of AI-allele status (Figure 3G). Moreover, it appeared to counter the AI-allele protective effect with a reduced age gap between those with and without AI alleles in BRAF p.Val600Glu tumors (Figure 3H). Regression analysis showed similar results, as p.Val600Glu mutation presence had a larger effect size than AI carrier status by almost 4 years (Figure 3I). Finally, we assessed whether either factor in isolation associated with increased $CD8^+$ T cell infiltration. We observed that while AI carrier status and BRAF p.Val600Glu mutation status shifted $CD8^+$ T cell infiltration levels insignificantly toward higher and lower means, respectively (Figure S15, $p_{autoimmune} = 0.090$, $p_{BRAF\ p.Val600Glu} = 0.147$), the conjunction of AI carrier status and the absence of a BRAF p.Val600Glu mutation significantly associated with increased $CD8^+$ T cell immune infiltration levels (Figure 3J, $p = 0.041$). Replacing TMB with neoantigen burden and BRAF p.Val600Glu status gave an estimated increase in $CD8^+$ T cell infiltration of 2.75% per MHC-I AI allele carried ($p = 0.039$).

(C) Fraction of driver mutations presented by AI alleles as a function of best rank score.
(D) Fraction of driver mutations presented by HLA-B AI alleles relative to common (≥1% population frequency; 19 alleles) HLA-B alleles. Maximum and minimum population allele coverage corresponds to the maximum and minimum fraction of driver mutations capable of being presented across common alleles at each best rank score, respectively.
(E) Fraction of driver mutations presented by HLA-C AI alleles relative to common (≥1% population frequency; 13 alleles) HLA-C alleles.
(F) TCGA: individuals with a BRAF p.Val600Glu mutation show a significant 9-years-earlier age of melanoma diagnosis relative to those without this mutation ($p_{adj} = 7.58 \times 10^{-6}$).
(G) TCGA: BRAF p.Val600Glu significantly reduces melanoma age of diagnosis across individuals independent of AI allele presence with median earlier ages of diagnosis of 8.5 years in those without an AI allele ($p = 7.39 \times 10^{-4}$) and 13 years in those with an AI allele ($p = 8.12 \times 10^{-7}$).
(H) TCGA: individuals with an AI allele show a significant median later age of diagnosis of 8 years in the absence of a BRAF p.Val600Glu mutation ($p = 2.98 \times 10^{-4}$). However BRAF p.Val600Glu mutation presence appears to counter the AI-allele-protective effect with a loss of significance between those with and without AI alleles ($p = 0.232$).
(I) TCGA: BRAF p.Val600Glu mutation presence is significantly associated with 7.91 earlier years to melanoma diagnosis ($p_{BRAF\ p.Val600Glu} = 7.12 \times 10^{-8}$, CI = [5.075, 10.750]), while having at least one MHC-I-linked AI allele is significantly associated with 3.97 delayed years to melanoma diagnosis ($p_{autoimmune} = 4.59 \times 10^{-3}$, CI = [1.230, 6.700]) after controlling for sex and mutation burden. Mutation burden also remained significant but had a minimal contribution to age of diagnosis with 0.003 delayed years until melanoma diagnosis ($p_{mutation} = 0.014$, CI = [0.001, 0.005]).
(J) TCGA: having at least one MHC-I-linked AI allele coupled with the absence of a BRAF p.Val600Glu mutation significantly associated with a 6% increase in $CD8^+$ T cell infiltration in primary melanoma tumors ($p = 0.041$).

Across AI alleles, only three were predicted to present BRAF p.Val600Glu. HLA-B*27:05 was the only allele with a predicted affinity below the strong binding cutoff (best rank score = 0.22). HLA-B*27:05-restricted cytotoxic T cell responses have been observed against p.Val600Glu.[164] HLA-B*39:06 and HLA-B*57:01 had scores of 1.78 and 0.61, respectively, showing potential for weaker p.Val600-Glu binding. We found no association with occurrence (OR = 1.04, $p = 0.843$) or expression of BRAF p.Val600Glu ($p = 0.336$) in individuals carrying AI alleles in general or those carrying one or more of these three AI alleles specifically. An association might have been expected if the mutant allele was subject to strong counter selection by immune surveillance. Notably, BRAF p.Val600Glu has been suggested to avoid immune surveillance by accelerating internalization of cell surface MHC-I.[165]

We next compared the affinity of MHC-I AI alleles for conserved antigens versus common alleles. Here, we considered differences in NetMHCPan-4.1 affinities both regionally and on average for 87 proteins, including known melanoma cancer antigens[130,151,156–162] and 52 genes that were both stably and specifically expressed in melanocytes and expressed in melanomas (Table S4). These 52 included four well-known melanocyte genes, *PMEL*, *MLANA*, *TYRP1*, and *TYR*. The rest were enriched for the folate metabolism pathway, which is important for DNA repair in melanocytes.[166] Evaluating expression in melanomas relative to melanocytes, we observed that two MAGE genes, *MAGEA10* and *MAGEE1* (MIM: 300759), were significantly upregulated and that several canonical melanocyte and stably expressed genes were downregulated (Figure 4A). This is consistent with reports that MAGE genes are specific to reproductive tissues[167] and tumors[156–162] and canonical melanocyte genes such as *TYRP1* are minimally expressed, if not undetectable, in melanoma.[154,168,169]

To evaluate broad allele-specific differences in conserved antigen-derived peptide repertoires, we compared the fraction of 8- to 11-mers from each conserved antigen predicted to bind at a given percentile rank (predicting binding affinities). Across AI alleles, we observed minimal variation; HLA-B*51:01 was the most promiscuous AI allele and HLA-B*13:02 was the second most promiscuous across the binding range (Figure 4B). However, in general, AI alleles exhibited a narrower conserved antigen repertoire relative to common alleles (Figures 4C and 4D). HLA-B generally presented a broader range of peptides across conserved antigens than HLA-C (Figure S13B). Considering individual conserved genes, AI alleles were generally predicted to bind a smaller fraction of derived peptides than common alleles. Comparison of the distributions of the top ten differences by mean at a gene-specific level showed that almost all MHC-I AI alleles present conserved antigens no better than common alleles (Figure S16), with the notable exception of HLA-B*27:05's affinity for SRSF8 (MIM: 603269) and TOMM6 (MIM: 616168) (Figures S16A and S16B).

Since MHC-I presented peptides are not uniformly sampled across the entire parent protein,[170] we revisited our analysis with a focus on regional differences by calculating position-wise best percentile ranks across all 8- to 11-mers overlapping a position in each gene (Figure S2A). We did not observe any region of a conserved antigen that was consistently more effectively presented by MHC-I AI alleles relative to common alleles, however there were short regions where specific MHC-I AI alleles had stronger affinity than common alleles (Figure S2B). In addition to binding SRSF8 more generally, HLA-B*27:05 also exhibited the greatest position-wise advantage to SRSF8 over common alleles (Figure S2B). This is in spite of having the smallest conserved antigen repertoire overall (Figures 4B and 4C). Several proteins had regions with affinity differences between HLA-B AI and common alleles, including the canonical melanocyte genes *PMEL*, *GPNMB* (MIM: 604368), and *TYR* and the MAGE genes *MAGEA10*, *MAGEE1*, *MAGED4* (MIM: 300702), and *MAGEA8* (MIM: 300341) (Figures S2B and S17). However, regional affinity advantages of AI alleles over common alleles tended to be allele, gene, and position specific with little overlap across AI alleles. Overall, these analyses suggest that the protective effect of MHC-I AI alleles does not derive from a shared specificity for a conserved antigen or neoantigen but cannot rule out that differences in specificity contribute to the effect.

Finally, we looked for trends suggesting selective pressure to evade immune responses on the basis of AI-allele presentation of conserved antigens. While we did not detect consistent downregulation of conserved antigens in tumors with AI alleles, LOH of AI alleles accounted for the greatest loss of potential to present peptides derived from conserved antigens in multiple cases (calculating hla loss of heterozygosity). In particular, for tumor antigens NSMCE3 (MIM: 608243), MTERF3 (MIM: 616930), MAGEF1 (MIM: 609267), MTX1 (MIM: 600605), GPR89B (MIM: 612806), MTFR1L, MAGED1 (MIM: 300224), and MTFR1 (MIM: 619414), loss of AI alleles resulted in a significant reduction in potential to present peptides from these proteins relative to other HLA alleles carried by the same individuals (Figures 4E and 4F, $p_{adj} < 0.05$). This suggests that LOH affecting AI alleles could allow tumors to evade T cell responses directed at certain conserved antigens.

Discussion

Immunosurveillance has been implicated in melanomagenesis prevention,[16,17,31] yet HLA contributions to melanoma risk have largely remained uncharacterized. Here, we investigated whether predisposing MHC-I alleles for $CD8^+$ T cell-driven skin-associated AI disorders (vitiligo and psoriasis) could protect against melanoma. Our findings support this hypothesis: AI-allele carriers exhibit a significant later age of melanoma diagnosis in the TCGA and a decreased risk of developing melanoma among individuals in the MVP. Moreover, AI-allele-specific protection appears

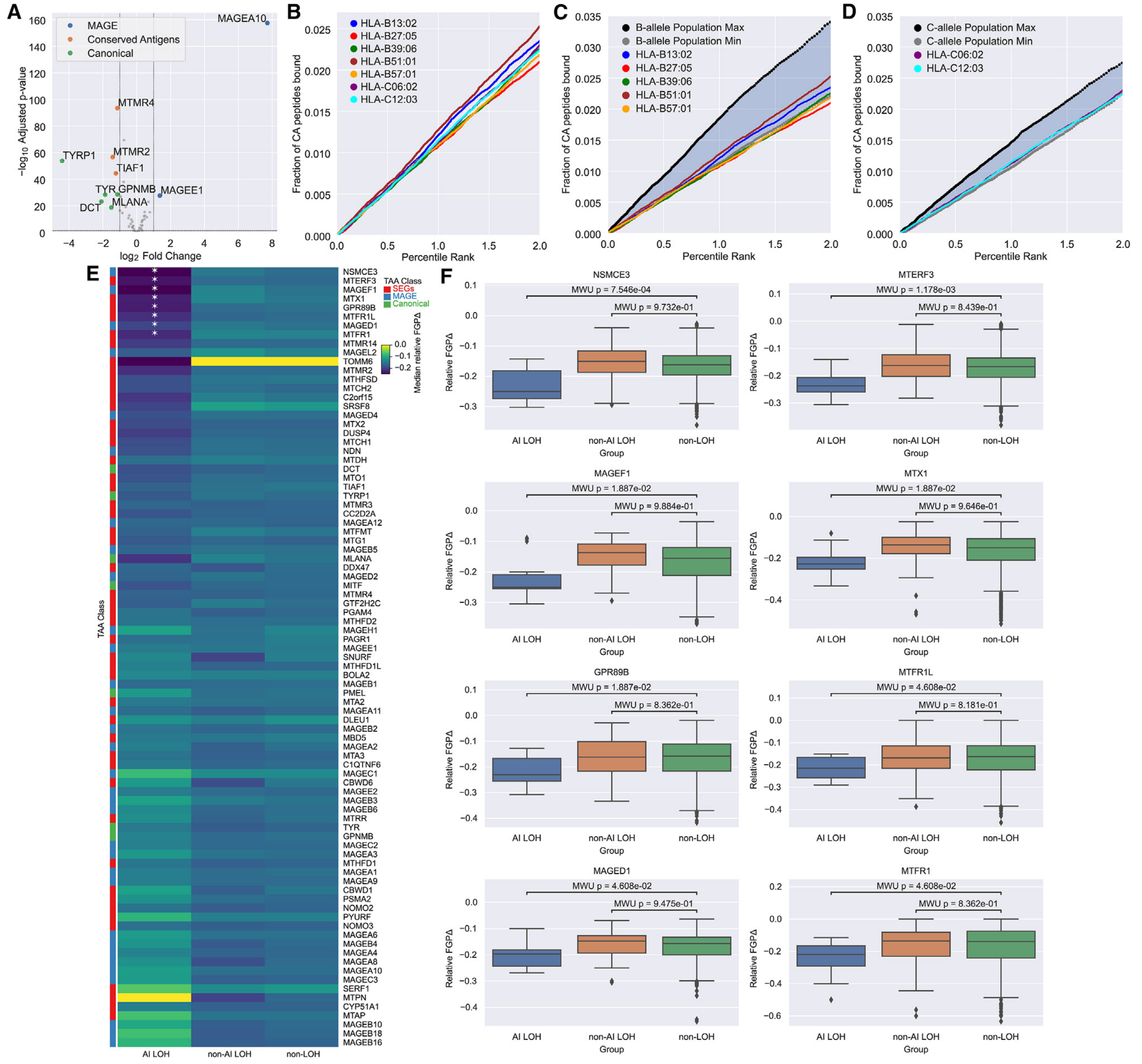

Figure 4. Conserved antigen coverage characterization including HLA LOH effects

(A) Differential expression of conserved antigens between normal melanocytes and melanoma. Labeled genes have an adjusted p value < 0.05 and an absolute fold change > 2.

(B) Fraction of peptides from conserved antigens (CAs) presented by each AI allele.

(C) Fraction of peptides from conserved antigens (CAs) presented by HLA-B AI alleles relative to common HLA-B alleles (≥ 1% population frequency; 19 alleles). Maximum and minimum population allele representations correspond to the maximum and minimum fraction of conserved antigen peptides bound across common alleles at each rank score, respectively.

(D) Fraction of peptides from conserved antigens (CAs) presented by HLA-C AI alleles relative to common HLA-C alleles (≥ 1% population frequency; 13 alleles).

(E) Median relative decrease in the fraction of a gene presented (FGP) by respective removal of AI alleles that were affected by loss of heterozygosity (AI LOH), non-AI alleles with LOH, and other alleles that were not affected by LOH from the same individuals. The "★" symbols denote decreases in FGP that are significantly lower than the effect of losing other alleles on the same genotype not affected by LOH by Mann-Whitney U test with a false discovery rate < 0.05.

(F) Distributions of the relative decrease in FGP for the eight genes with the greatest loss in presentation attributable to observed LOH of AI alleles.

not only to be uncaptured by current melanoma PRSs[76] but also can augment PRS performance in relative risk stratification. While at least six vitiligo risk SNPs are protective from melanoma,[28,29] our results show that AI-risk effects can be extended to MHC-I as well and suggest a broader space of joint AI predisposition and melanoma protection.

We further investigated potential mechanisms linking MHC-I AI alleles to delayed age at diagnosis. Immune

activity against melanoma has been linked to high mutation burden due to UV exposure,[21,22] suggesting neoantigens could provide a potential substrate. Focusing on mutations that drive melanomagenesis, we did not see obvious differences in the affinity for MHC-I AI alleles for neopeptides relative to other alleles. While AI alleles did not seem to interact with specific mutations, we did note that BRAF p.Val600Glu was associated with an earlier age at diagnosis independent of AI-allele carrier status. Potential of AI alleles to present BRAF p.Val600Glu did not appear to impact the incidence of the mutation, which is consistent with reports that this mutation associates with impaired immune surveillance.[165]

As CD8$^+$ T cells target healthy melanocytes through conserved antigens in both psoriasis and vitiligo, responses against conserved antigens could also provide an explanation. We observed positions better presented by AI alleles in genes spanning all subcategories of conserved antigens including canonical melanocyte genes, melanoma antigen genes, and genes stably expressed in melanocytes. In particular, certain amino acids in PMEL and TYR (Figure S2B), which are melanocyte-specific antigens recognized by CD8$^+$ T cells in melanoma-associated vitiligo,[130,151] were better presented by AI alleles. However, we did not observe position-wise advantages for AI alleles in other CD8$^+$ T cell targets, such as MLANA or DCT. MHC-I AI alleles also uniquely target positions in the melanoma antigen genes *MAGEA10* and *MAGEE1*, which are upregulated in melanoma relative to melanocytes (Figures 4A and S2B). Finally, we observed positions uniquely targeted by AI alleles within melanocyte stably expressed genes. Notably, SRSF8 exhibited the position most favored by AI alleles and is overall substantially better targeted by the AI allele, HLA-B*27:05 (Figure S16). This suggests that melanocyte-specific conserved antigens, other than those already identified in melanoma-associated vitiligo, may also contribute to the protective effect of AI alleles. Additionally, while canonical melanocyte genes have been known to be recognized by CD8$^+$ T cells, they are typically downregulated in melanoma (Figure 4A) or otherwise inconsistently expressed.[171,172] In contrast, the tumor-specific antigens considered in this study exhibit stable expression across melanocytes and melanoma and might serve as more consistent immune targets.

Altogether, these findings support that AI alleles' unique immunopeptidomes could contribute to their protective effect against melanoma. However, further investigation is needed. In particular, some studies have suggested that stability of the MHC-I-peptide complex distinguishes AI alleles from other MHC-I alleles,[173–175] so it is possible that the mechanism is not fully dependent on antigen specificity. There is also evidence that different alleles result in qualitatively different T cell responses. While the seven AI alleles studied here associated with lower levels of regulatory T cell infiltration (Figure 1B), *HLA-A*02:01* did not (Figures S5F and S5G), despite documented associations with vitiligo. This may relate to increased expression of TIM-3 by CD8$^+$ T cells induced by HLA-A*02:01 restriction.[146] TIM-3 interacts with galectin-9 (MIM: 601879) expressed by regulatory T cells (and certain tumor cells) to induce CD8$^+$ T cell apoptosis.[176–178] In contrast, CD8$^+$ T cells restricted by HLA-B*27 or HLA-B*57, upon epitope recognition, do not upregulate TIM-3[146] but instead upregulate granzyme B[179] (MIM: 123910), killing the regulatory T cells they encounter. More generally, it has been observed that healthy individuals carrying *HLA-B*27/B*57* alleles have lower levels of regulatory T cells.[146] Overall, protective effects were not shared by broader HLA supertype groupings, supporting that allele-specific characteristics, whether related to unique antigen specificities, stability, or some other characteristic, are likely to account for any protective effect.

In general, AI alleles presented a narrower peptide repertoire compared to common alleles (Figures 4C and 4D). Notably, HLA-B*27:05 and HLA-B*57:01, both of which covered a smaller fraction of potential conserved peptides than the minimum HLA-B population allele representation, are known for their fastidiousness or narrow peptide-binding repertoire, as studied in the context of progression from HIV infection (MIM: 609423) to AIDS.[180] Košmrlj et al. also observed that fastidious alleles present peptides not found in common alleles' repertoires. We similarly observed that the most fastidious AI alleles, HLA-B*27:05 and HLA-B*57:01, exhibited the greatest position-wise advantages over common alleles. Additionally, fastidious class I alleles are expressed on the cell surface at much higher levels than their promiscuous class I counterparts [181] and therefore most likely offer more opportunities for both neoepitope and self-antigen presentation from their binding repertoires. Moreover, CD8$^+$ T cells restricted by fastidious alleles (e.g., HLA-B*57:01) may also be more cross-reactive as they are subject to less negative selection in the thymus given the narrower set of self-peptides to which they are exposed.[180] Interestingly, we note that outside of AI associations four of our seven AI-alleles (*HLA-B*27:05*, *HLA-B*51:01*, *HLA-C*06:02*, and *HLA-B*57:01*) are among the strongest HIV-protective alleles.[124,182,183]

Peptide-MHC (pMHC) affinity is driven largely by MHC-I sequence variation where specific polymorphisms shape the binding pockets in the peptide-binding groove. Unsurprisingly, given the skin-specific AI associations for which these alleles were selected, certain AI-alleles have similar binding pocket characteristics. For example, HLA-C*06:02 and HLA-C*12:03 both share a strongly negative E-pocket.[184] HLA-C*06:02 shares an electronegative B-pocket with HLA-B*27:05 as well.[184,185] An allele's affinity-based presentable peptide repertoire, though, is an idealistic representation that fails to account for antigen processing pathway contributions. Ultimately the space of bound peptides presented on the cell surface is far narrower. Endoplasmic reticulum aminopeptidases (ERAP) 1 (MIM: 606832) and 2 (MIM: 609497) play an essential role in MHC-I antigen processing. They function to clip peptides to the appropriate size for MHC-I binding[186,187] but can also destroy potential ligands through overtrimming.[188–191] *ERAP1* and *ERAP2* are prominent risk factors

in MHC-I linked AI conditions and both are associated with psoriasis risk.[192–194] Epistatic effects between *ERAP1* and AI-risk alleles have been observed, particularly with *HLA-C*06:02* in psoriasis,[192] and suggest this risk interaction is tied to an increased likelihood of specific autoantigens making it to the cell surface. Taken together, this suggests skin-specific AI predisposing *ERAP* genes may also confer melanoma protection and is an interesting area for further pursuit.

Work by Chowell et al.[49] and Cummings et al.[195] showed that in melanoma the B44 supertype associates with extended survival after treatment with immune-checkpoint inhibitors (ICPIs). While none of our AI alleles fall within this supertype, we surprisingly observed that the B44 supertype trended toward an earlier age of diagnosis in our discovery set (Figure S7). The B44 supertype has an electropositive B-pocket, with an affinity for negatively charged residues at P2 such as glutamic acid (Glu).[196] Given this strong glutamic acid affinity, it may be that in the context of ICPI, the B44 supertype is capable of inducing an immune response through the binding and presentation of the highly recurrent BRAF p.Val600Glu mutation. In contrast, the MHC-I AI alleles associated with later age at diagnosis do not bind negatively charged residues at P2. In fact, HLA-B*27:05 and HLA-C*06:02 have electronegative B-pockets with strong affinities for the positively charged arginine at position 2[184,185] and effectively serve as B44 antonyms. This suggests peptide repertoire differences between the B44 supertype and the AI allele set and further suggests the potential for a dichotomy between MHC-I-associated melanoma protection and ICPI response, which is associated with somatic mutation presentation and high tumor mutation burden.

In conclusion, our study supports that skin-specific AI MHC alleles have a protective effect in melanoma. Additional assessments of these alleles' effect on age of onset in further independent cohorts as they become available would be of future interest. We also note the potential for MHC-I-mediated autoimmunity to interact with cancer development more broadly. Selecting alleles associated with AI disease(s) affecting the tissue type under investigation may yield similar findings across cancer types. For example, some MHC-I alleles are associated with multiple AI conditions, such as *HLA-B*27:05* in both psoriasis and ankylosing spondylitis (MIM: 106300).[123,124,126,127,197,198] Ankylosing spondylitis is a targeted form of spinal arthritis primarily affecting the entheses and leads to bone erosion and broad vertebral fusion[199] and makes for a conceivable AI counterpart to osteosarcomas. Our study did not address MHC-II (MIM: 142860) alleles, which are generally expressed more specifically by antigen-presenting cells but have nonetheless been implicated in both skin AI disorders and immunosurveillance. Taken together, tissue-specific MHC AI carrier status may broaden the scope of the AI-cancer risk interplay and remains an interesting area for further exploration.

Data and code availability

All data for this project were obtained from public sources. Discovery set: data were obtained from The Cancer Genome Atlas (TCGA) Research Network (http://cancergenome.nih.gov/). Normal exome sequences and clinical data were downloaded from the GDC on June 23–26, 2018 and April 25, respectively, with the gdc-client v1.3.0. Somatic mutations were accessed from the NCI Genomic Data Commons (https://portal.gdc.cancer.gov/) on May 14, 2017. Genotype calls for TCGA were accessed from GDC on April 26, 2019. Validation and stably expressed gene datasets: dbGaP data from datasets dbGaP: phs000452.v2.p1.c2, phs001550.v2.p1.c1, phs000933.v2.p1.c1, and phs001500.v1.p1.c1 were obtained with AsperaConnect v3.9.5.172984. SRA toolkit v2.9.2 was used to obtain WXS/WGS data from the Sequence Read Archive (SRA) (including from the following studies SRA: SRP067938 and SRA: SRP090294). UKBB data was retrieved under project ID 37671. Million Veteran Program: genotype and phenotype information were obtained through application. Melanostrum dataset: genotype data from Gu et al.[76] were obtained by direct communication with the authors. Code for all analyses can be found at https://github.com/cartercompbio/MelMHC/.

Supplemental information

Supplemental information can be found online at https://doi.org/10.1016/j.ajhg.2023.05.013.

Acknowledgments

This work was supported by a grant from the Harry J. Lloyd Charitable Trust (20191857) to H.C., an Emerging Leader Award from The Mark Foundation for Cancer Research (18-022-ELA) to H.C., NIH (National Institutes of Health) grant RO1 CA220009 grant to M.Z. and H.C., NIH grant R01 MH122688-02 grant to W.K.T., an NIH National Library of Medicine training grant (T15LM011271) to A.C., and NCI (National Cancer Institute) grant (U24CA248138). Computational infrastructure was funded by NIH grant 2P41GM103504-11. The results shown here are in large part based upon data generated by the TCGA Research Network (https://www.cancer.gov/tcga), the UKBB (project #37671), and the following studies: dbGaP: phs000452.v2.p1.c2, phs000933.v2.p1.c1, phs001550.v2.p1.c1, phs001500.v1.p1.c1, phs000424.v7.p2, and GEO: GSE78220. This research also used data from the Million Veteran Program (MVP), Office of Research and Development, Veterans Health Administration. This research was supported by the Million Veteran Program MVP022 award #I01 CX001727 (PI: R.H.). MVP data access was provided under the Genisis Core Project. This publication does not represent the views of the Department of Veterans Affairs or the United States Government. The graphical abstract was created with BioRender.com.

Author contributions

Original concept, J.V.T. and H.C.; project supervision, H.C.; project planning and design, J.V.T. and H.C.; data acquisition, processing, and analysis, J.V.T., D.L., M.S.P., and A.C.; melanoma sojourn time estimation, M.L., G.E.L., and K.C.; statistical advising, W.K.T.; UKBB data acquisition, R.M.S.; MVP data acquisition and processing, B.R.G., C.P., F.N.D., K.M., R.H., C.C.T., J.L., S.P., and P.S.T.; immunological interpretation advising, G.P.M. and M.Z.; preparation of the manuscript, J.V.T., D.L., and H.C.

Declaration of interests

G.P.M. receives research support from Thermo Fisher, CareDx, and PIRCHE. M.Z. is a board member of Invectys Inc.

Received: July 13, 2022
Accepted: May 22, 2023
Published: June 19, 2023

References

1. Ali, Z., Yousaf, N., and Larkin, J. (2013). Melanoma epidemiology, biology and prognosis. EJC Suppl. *11*, 81–91.
2. Matthews, N.H., Li, W.-Q., Qureshi, A.A., Weinstock, M.A., and Cho, E. (2018). Epidemiology of Melanoma. In Cutaneous Melanoma: Etiology and Therapy, W.H. Ward and J.M. Farma, eds. (Codon Publications)), pp. 3–22.
3. Howlader, N., Noone, A.M., Krapcho, M., Miller, D., Brest, A., Yu, M., Ruhl, J., Tatalovich, Z., Mariotto, A., Lewis, D.R., et al (eds) SEER Cancer Statistics Review, 1975-2017. National Cancer Institute. Bethesda, MD, https://seer.cancer.gov/csr/1975_2017/, based on November 2019 SEER data submission, posted to the SEER web site, April 2020.
4. Gilchrest, B.A., Eller, M.S., Geller, A.C., and Yaar, M. (1999). The pathogenesis of melanoma induced by ultraviolet radiation. N. Engl. J. Med. *340*, 1341–1348.
5. Lea, C.S., Scotto, J.A., Buffler, P.A., Fine, J., Barnhill, R.L., and Berwick, M. (2007). Ambient UVB and melanoma risk in the United States: a case-control analysis. Ann. Epidemiol. *17*, 447–453.
6. Garibyan, L., and Fisher, D.E. (2010). How sunlight causes melanoma. Curr. Oncol. Rep. *12*, 319–326.
7. Lin, J.Y., and Fisher, D.E. (2007). Melanocyte biology and skin pigmentation. Nature *445*, 843–850.
8. Rees, J.L. (2003). Genetics of hair and skin color. Annu. Rev. Genet. *37*, 67–90.
9. Olsen, C.M., Carroll, H.J., and Whiteman, D.C. (2010). Estimating the attributable fraction for melanoma: a meta-analysis of pigmentary characteristics and freckling. Int. J. Cancer *127*, 2430–2445.
10. Chang, Y.-M., Newton-Bishop, J.A., Bishop, D.T., Armstrong, B.K., Bataille, V., Bergman, W., Berwick, M., Bracci, P.M., Elwood, J.M., Ernstoff, M.S., et al. (2009). A pooled analysis of melanocytic nevus phenotype and the risk of cutaneous melanoma at different latitudes. Int. J. Cancer *124*, 420–428.
11. Olsen, C.M., Carroll, H.J., and Whiteman, D.C. (2010). Estimating the attributable fraction for cancer: A meta-analysis of nevi and melanoma. Cancer Prev. Res. *3*, 233–245.
12. Schuchter, L., Schultz, D.J., Synnestvedt, M., Trock, B.J., Guerry, D., Elder, D.E., Elenitsas, R., Clark, W.H., and Halpern, A.C. (1996). A prognostic model for predicting 10-year survival in patients with primary melanoma. The Pigmented Lesion Group. Ann. Intern. Med. *125*, 369–375.
13. Williams, L.H., Shors, A.R., Barlow, W.E., Solomon, C., and White, E. (2011). Identifying Persons at Highest Risk of Melanoma Using Self-Assessed Risk Factors. J. Clin. Exp. Dermatol. Res. *2*, 1000129.
14. Han, J., Qureshi, A.A., Prescott, J., Guo, Q., Ye, L., Hunter, D.J., and De Vivo, I. (2009). A prospective study of telomere length and the risk of skin cancer. J. Invest. Dermatol. *129*, 415–421.
15. Bataille, V., Kato, B.S., Falchi, M., Gardner, J., Kimura, M., Lens, M., Perks, U., Valdes, A.M., Bennett, D.C., Aviv, A., and Spector, T.D. (2007). Nevus Size and Number Are Associated with Telomere Length and Represent Potential Markers of a Decreased Senescence In vivo. Cancer Epidemiol. Biomarkers Prev. *16*, 1499–1502.
16. Green, A.C., and Olsen, C.M. (2015). Increased risk of melanoma in organ transplant recipients: systematic review and meta-analysis of cohort studies. Acta Derm. Venereol. *95*, 923–927.
17. Greene, M.H., Young, T.I., and Clark, W.H., Jr. (1981). Malignant melanoma in renal-transplant recipients. Lancet *1*, 1196–1199.
18. Olsen, C.M., Carroll, H.J., and Whiteman, D.C. (2010). Familial melanoma: a meta-analysis and estimates of attributable fraction. Cancer Epidemiol. Biomarkers Prev. *19*, 65–73.
19. Ford, D., Bliss, J.M., Swerdlow, A.J., Armstrong, B.K., Franceschi, S., Green, A., Holly, E.A., Mack, T., MacKie, R.M., and Osterlind, A. (1995). Risk of cutaneous melanoma associated with a family history of the disease. The International Melanoma Analysis Group (IMAGE). Int. J. Cancer *62*, 377–381.
20. Mucci, L.A., Hjelmborg, J.B., Harris, J.R., Czene, K., Havelick, D.J., Scheike, T., Graff, R.E., Holst, K., Möller, S., Unger, R.H., et al. (2016). Familial Risk and Heritability of Cancer Among Twins in Nordic Countries. JAMA *315*, 68–76.
21. Greenman, C., Stephens, P., Smith, R., Dalgliesh, G.L., Hunter, C., Bignell, G., Davies, H., Teague, J., Butler, A., Stevens, C., et al. (2007). Patterns of somatic mutation in human cancer genomes. Nature *446*, 153–158.
22. Alexandrov, L.B., Nik-Zainal, S., Wedge, D.C., Aparicio, S.A.J.R., Behjati, S., Biankin, A.V., Bignell, G.R., Bolli, N., Borg, A., Børresen-Dale, A.L., et al. (2013). Signatures of mutational processes in human cancer. Nature *500*, 415–421.
23. Clemente, C.G., Mihm, M.C., Jr., Bufalino, R., Zurrida, S., Collini, P., and Cascinelli, N. (1996). Prognostic value of tumor infiltrating lymphocytes in the vertical growth phase of primary cutaneous melanoma. Cancer *77*, 1303–1310.
24. Morton, D., Eilber, F.R., Malmgren, R.A., and Wood, W.C. (1970). Immunological factors which influence response to immunotherapy in malignant melanoma. Surgery *68*, 158–163.
25. Orrù, V., Steri, M., Sole, G., Sidore, C., Virdis, F., Dei, M., Lai, S., Zoledziewska, M., Busonero, F., Mulas, A., et al. (2013). Genetic variants regulating immune cell levels in health and disease. Cell *155*, 242–256.
26. Mangino, M., Roederer, M., Beddall, M.H., Nestle, F.O., and Spector, T.D. (2017). Innate and adaptive immune traits are differentially affected by genetic and environmental factors. Nat. Commun. *8*, 13850–13857.
27. Marderstein, A.R., Uppal, M., Verma, A., Bhinder, B., Tayyebi, Z., Mezey, J., Clark, A.G., and Elemento, O. (2020). Demographic and genetic factors influence the abundance of infiltrating immune cells in human tissues. Nat. Commun. *11*, 2213–2214.
28. Jin, Y., Birlea, S.A., Fain, P.R., Ferrara, T.M., Ben, S., Riccardi, S.L., Cole, J.B., Gowan, K., Holland, P.J., Bennett, D.C., et al. (2012). Genome-wide association analyses identify 13 new susceptibility loci for generalized vitiligo. Nat. Genet. *44*, 676–680.
29. Jin, Y., Andersen, G., Yorgov, D., Ferrara, T.M., Ben, S., Brownson, K.M., Holland, P.J., Birlea, S.A., Siebert, J.,

Hartmann, A., et al. (2016). Genome-wide association studies of autoimmune vitiligo identify 23 new risk loci and highlight key pathways and regulatory variants. Nat. Genet. *48*, 1418–1424.
30. Landi, M.T., Bishop, D.T., MacGregor, S., Machiela, M.J., Stratigos, A.J., Ghiorzo, P., Brossard, M., Calista, D., Choi, J., Fargnoli, M.C., et al. (2020). Genome-wide association meta-analyses combining multiple risk phenotypes provide insights into the genetic architecture of cutaneous melanoma susceptibility. Nat. Genet. *52*, 494–504.
31. Corthay, A. (2014). Does the immune system naturally protect against cancer? Front. Immunol. *5*, 197.
32. Teng, M.W.L., Swann, J.B., Koebel, C.M., Schreiber, R.D., and Smyth, M.J. (2008). Immune-mediated dormancy: an equilibrium with cancer. J. Leukoc. Biol. *84*, 988–993.
33. Gregersen, P.K., and Behrens, T.W. (2006). Genetics of autoimmune diseases–disorders of immune homeostasis. Nat. Rev. Genet. *7*, 917–928.
34. Nanda, N.K., and Sercarz, E.E. (1995). Induction of anti-self-immunity to cure cancer. Cell *82*, 13–17.
35. Pardoll, D.M. (1999). Inducing autoimmune disease to treat cancer. Proc. Natl. Acad. Sci. USA *96*, 5340–5342.
36. Spritz, R.A. (2010). The genetics of generalized vitiligo: autoimmune pathways and an inverse relationship with malignant melanoma. Genome Med. *2*, 78.
37. Young, A., Quandt, Z., and Bluestone, J.A. (2018). The Balancing Act between Cancer Immunity and Autoimmunity in Response to Immunotherapy. Cancer Immunol. Res. *6*, 1445–1452.
38. Chat, V., Ferguson, R., Simpson, D., Kazlow, E., Lax, R., Moran, U., Pavlick, A., Frederick, D., Boland, G., Sullivan, R., et al. (2019). Autoimmune genetic risk variants as germline biomarkers of response to melanoma immune-checkpoint inhibition. Cancer Immunol. Immunother. *68*, 897–905.
39. Khan, Z., Di Nucci, F., Kwan, A., Hammer, C., Mariathasan, S., Rouilly, V., Carroll, J., Fontes, M., Ley Acosta, S., Guardino, E., et al. (2020). Polygenic risk for skin autoimmunity impacts immune checkpoint blockade in bladder cancer. Proc. Natl. Acad. Sci. USA *117*, 12288–12294.
40. Askling, J., Fored, C.M., Brandt, L., Baecklund, E., Bertilsson, L., Feltelius, N., Cöster, L., Geborek, P., Jacobsson, L.T., Lindblad, S., et al. (2005). Risks of solid cancers in patients with rheumatoid arthritis and after treatment with tumour necrosis factor antagonists. Ann. Rheum. Dis. *64*, 1421–1426.
41. Song, L., Wang, Y., Zhang, J., Song, N., Xu, X., and Lu, Y. (2018). The risks of cancer development in systemic lupus erythematosus (SLE) patients: a systematic review and meta-analysis. Arthritis Res. Ther. *20*, 270.
42. Teulings, H.E., Overkamp, M., Ceylan, E., Nieuweboer-Krobotova, L., Bos, J.D., Nijsten, T., Wolkerstorfer, A.W., Luiten, R.M., and van der Veen, J.P.W. (2013). Decreased risk of melanoma and nonmelanoma skin cancer in patients with vitiligo: a survey among 1307 patients and their partners. Br. J. Dermatol. *168*, 162–171.
43. Rock, K.L., Reits, E., and Neefjes, J. (2016). Present Yourself! By MHC Class I and MHC Class II Molecules. Trends Immunol. *37*, 724–737.
44. Durgeau, A., Virk, Y., Corgnac, S., and Mami-Chouaib, F. (2018). Recent Advances in Targeting CD8 T-Cell Immunity for More Effective Cancer Immunotherapy. Front. Immunol. *9*, 14.
45. Woodsworth, D.J., Dunsing, V., and Coombs, D. (2015). Design Parameters for Granzyme-Mediated Cytotoxic Lymphocyte Target-Cell Killing and Specificity. Biophys. J. *109*, 477–488.
46. Marty, R., Kaabinejadian, S., Rossell, D., Slifker, M.J., van de Haar, J., Engin, H.B., de Prisco, N., Ideker, T., Hildebrand, W.H., Font-Burgada, J., and Carter, H. (2017). MHC-I Genotype Restricts the Oncogenic Mutational Landscape. Cell *171*, 1272–1283.e15.
47. Marty Pyke, R., Thompson, W.K., Salem, R.M., Font-Burgada, J., Zanetti, M., and Carter, H. (2018). Evolutionary Pressure against MHC Class II Binding Cancer Mutations. Cell *175*, 1991–2428.e13.
48. Yang, F., Kim, D.-K., Nakagawa, H., Hayashi, S., Imoto, S., Stein, L., and Roth, F.P. (2019). Quantifying immune-based counterselection of somatic mutations. PLoS Genet. *15*, e1008227.
49. Chowell, D., Morris, L.G.T., Grigg, C.M., Weber, J.K., Samstein, R.M., Makarov, V., Kuo, F., Kendall, S.M., Requena, D., Riaz, N., et al. (2018). Patient HLA class I genotype influences cancer response to checkpoint blockade immunotherapy. Science *359*, 582–587.
50. Chowell, D., Krishna, C., Pierini, F., Makarov, V., Rizvi, N.A., Kuo, F., Morris, L.G.T., Riaz, N., Lenz, T.L., and Chan, T.A. (2019). Evolutionary divergence of HLA class I genotype impacts efficacy of cancer immunotherapy. Nat. Med. *25*, 1715–1720.
51. Goodman, A.M., Castro, A., Pyke, R.M., Okamura, R., Kato, S., Riviere, P., Frampton, G., Sokol, E., Zhang, X., Ball, E.D., et al. (2020). MHC-I genotype and tumor mutational burden predict response to immunotherapy. Genome Med. *12*, 45.
52. McGranahan, N., Rosenthal, R., Hiley, C.T., Rowan, A.J., Watkins, T.B.K., Wilson, G.A., Birkbak, N.J., Veeriah, S., Van Loo, P., Herrero, J., et al. (2017). Allele-Specific HLA Loss and Immune Escape in Lung Cancer Evolution. Cell *171*, 1259–1271.e11.
53. Castro, A., Ozturk, K., Pyke, R.M., Xian, S., Zanetti, M., and Carter, H. (2019). Elevated neoantigen levels in tumors with somatic mutations in the HLA-A, HLA-B, HLA-C and B2M genes. BMC Med. Genomics *12*, 107.
54. van den Boorn, J.G., Konijnenberg, D., Dellemijn, T.A.M., van der Veen, J.P.W., Bos, J.D., Melief, C.J.M., Vyth-Dreese, F.A., and Luiten, R.M. (2009). Autoimmune destruction of skin melanocytes by perilesional T cells from vitiligo patients. J. Invest. Dermatol. *129*, 2220–2232.
55. Boehncke, W.-H., and Brembilla, N.C. (2019). Autoreactive T-Lymphocytes in Inflammatory Skin Diseases. Front. Immunol. *10*, 1198.
56. Conrad, C., Boyman, O., Tonel, G., Tun-Kyi, A., Laggner, U., de Fougerolles, A., Kotelianski, V., Gardner, H., and Nestle, F.O. (2007). α 1 β 1 integrin is crucial for accumulation of epidermal T cells and the development of psoriasis. Nat. Med. *13*, 836–842.
57. Cai, Y., Fleming, C., and Yan, J. (2012). New insights of T cells in the pathogenesis of psoriasis. Cell. Mol. Immunol. *9*, 302–309.
58. Prinz, J.C. (2018). Human Leukocyte Antigen-Class I Alleles and the Autoreactive T Cell Response in Psoriasis Pathogenesis. Front. Immunol. *9*, 954.
59. Arakawa, A., Siewert, K., Stöhr, J., Besgen, P., Kim, S.-M., Rühl, G., Nickel, J., Vollmer, S., Thomas, P., Krebs, S., et al.

(2015). Melanocyte antigen triggers autoimmunity in human psoriasis. J. Exp. Med. *212*, 2203–2212.
60. Zhu, K.-J., Lv, Y.-M., Yin, X.-Y., Wang, Z.-X., Sun, L.-D., He, S.-M., Cheng, H., Hu, D.-Y., Zhang, Z., Li, Y., et al. (2011). Psoriasis regression analysis of MHC loci identifies shared genetic variants with vitiligo. PLoS One *6*, e23089.
61. Sharquie, K.E., Salman, H.A., and Yaseen, A.K. (2017). Psoriasis and vitiligo are close relatives. Clin. Cosmet. Investig. Dermatol. *10*, 341–345.
62. Vashist, S., Mahajan, V.K., Mehta, K.S., Chauhan, P.S., Yadav, R.S., Sharma, S.B., Sharma, V., Sharma, A., Chowdhary, B., and Kumar, P. (2020). Association of Psoriasis with Autoimmune Disorders: Results of a Pilot Study. Indian Dermatol. Online J. *11*, 753–759.
63. Rosenberg, S.A., and White, D.E. (1996). Vitiligo in patients with melanoma: normal tissue antigens can be targets for cancer immunotherapy. J. Immunother. Emphasis Tumor Immunol. *19*, 81–84.
64. Hua, C., Boussemart, L., Mateus, C., Routier, E., Boutros, C., Cazenave, H., Viollet, R., Thomas, M., Roy, S., Benannoune, N., et al. (2016). Association of Vitiligo With Tumor Response in Patients With Metastatic Melanoma Treated With Pembrolizumab. JAMA Dermatol. *152*, 45–51.
65. Nakamura, Y., Tanaka, R., Asami, Y., Teramoto, Y., Imamura, T., Sato, S., Maruyama, H., Fujisawa, Y., Matsuya, T., Fujimoto, M., and Yamamoto, A. (2017). Correlation between vitiligo occurrence and clinical benefit in advanced melanoma patients treated with nivolumab: A multi-institutional retrospective study. J. Dermatol. *44*, 117–122.
66. Indini, A., Di Guardo, L., Cimminiello, C., Prisciandaro, M., Randon, G., De Braud, F., and Del Vecchio, M. (2019). Immune-related adverse events correlate with improved survival in patients undergoing anti-PD1 immunotherapy for metastatic melanoma. J. Cancer Res. Clin. Oncol. *145*, 511–521.
67. Hofmann, L., Forschner, A., Loquai, C., Goldinger, S.M., Zimmer, L., Ugurel, S., Schmidgen, M.I., Gutzmer, R., Utikal, J.S., Göppner, D., et al. (2016). Cutaneous, gastrointestinal, hepatic, endocrine, and renal side-effects of anti-PD-1 therapy. Eur. J. Cancer *60*, 190–209.
68. Bonigen, J., Raynaud-Donzel, C., Hureaux, J., Kramkimel, N., Blom, A., Jeudy, G., Breton, A.-L., Hubiche, T., Bedane, C., Legoupil, D., et al. (2017). Anti-PD1-induced psoriasis: a study of 21 patients. J. Eur. Acad. Dermatol. Venereol. *31*, e254–e257.
69. Shukla, S.A., Rooney, M.S., Rajasagi, M., Tiao, G., Dixon, P.M., Lawrence, M.S., Stevens, J., Lane, W.J., Dellagatta, J.L., Steelman, S., et al. (2015). Comprehensive analysis of cancer-associated somatic mutations in class I HLA genes. Nat. Biotechnol. *33*, 1152–1158.
70. Kawaguchi, S., Higasa, K., Shimizu, M., Yamada, R., and Matsuda, F. (2017). HLA-HD: An accurate HLA typing algorithm for next-generation sequencing data. Hum. Mutat. *38*, 788–797.
71. Hugo, W., Zaretsky, J.M., Sun, L., Song, C., Moreno, B.H., Hu-Lieskovan, S., Berent-Maoz, B., Pang, J., Chmielowski, B., Cherry, G., et al. (2016). Genomic and Transcriptomic Features of Response to Anti-PD-1 Therapy in Metastatic Melanoma. Cell *165*, 35–44.
72. Van Allen, E.M., Miao, D., Schilling, B., Shukla, S.A., Blank, C., Zimmer, L., Sucker, A., Hillen, U., Foppen, M.H.G., Goldinger, S.M., et al. (2015). Genomic correlates of response to CTLA-4 blockade in metastatic melanoma. Science *350*, 207–211.
73. Krauthammer, M., Kong, Y., Bacchiocchi, A., Evans, P., Pornputtapong, N., Wu, C., McCusker, J.P., Ma, S., Cheng, E., Straub, R., et al. (2015). Exome sequencing identifies recurrent mutations in NF1 and RASopathy genes in sun-exposed melanomas. Nat. Genet. *47*, 996–1002.
74. Lazova, R., Pornputtapong, N., Halaban, R., Bosenberg, M., Bai, Y., Chai, H., and Krauthammer, M. (2017). Spitz nevi and Spitzoid melanomas: exome sequencing and comparison with conventional melanocytic nevi and melanomas. Mod. Pathol. *30*, 640–649.
75. Shain, A.H., Yeh, I., Kovalyshyn, I., Sriharan, A., Talevich, E., Gagnon, A., Dummer, R., North, J., Pincus, L., Ruben, B., et al. (2015). The Genetic Evolution of Melanoma from Precursor Lesions. N. Engl. J. Med. *373*, 1926–1936.
76. Gu, F., Chen, T.-H., Pfeiffer, R.M., Fargnoli, M.C., Calista, D., Ghiorzo, P., Peris, K., Puig, S., Menin, C., De Nicolo, A., et al. (2018). Combining common genetic variants and non-genetic risk factors to predict risk of cutaneous melanoma. Hum. Mol. Genet. *27*, 4145–4156.
77. Fang, H., Hui, Q., Lynch, J., Honerlaw, J., Assimes, T.L., Huang, J., Vujkovic, M., Damrauer, S.M., Pyarajan, S., Gaziano, J.M., et al. (2019). Harmonizing Genetic Ancestry and Self-identified Race/Ethnicity in Genome-wide Association Studies. Am. J. Hum. Genet. *105*, 763–772.
78. Denny, J.C., Bastarache, L., Ritchie, M.D., Carroll, R.J., Zink, R., Mosley, J.D., Field, J.R., Pulley, J.M., Ramirez, A.H., Bowton, E., et al. (2013). Systematic comparison of phenome-wide association study of electronic medical record data and genome-wide association study data. Nat. Biotechnol. *31*, 1102–1110.
79. Zheng, X., Shen, J., Cox, C., Wakefield, J.C., Ehm, M.G., Nelson, M.R., and Weir, B.S. (2014). HIBAG—HLA genotype imputation with attribute bagging. Pharmacogenomics J. *14*, 192–200.
80. Degenhardt, F., Wendorff, M., Wittig, M., Ellinghaus, E., Datta, L.W., Schembri, J., Ng, S.C., Rosati, E., Hübenthal, M., Ellinghaus, D., et al. (2019). Construction and benchmarking of a multi-ethnic reference panel for the imputation of HLA class I and II alleles. Hum. Mol. Genet. *28*, 2078–2092.
81. Hunter-Zinck, H., Shi, Y., Li, M., Gorman, B.R., Ji, S.-G., Sun, N., Webster, T., Liem, A., Hsieh, P., Devineni, P., et al. (2020). Genotyping Array Design and Data Quality Control in the Million Veteran Program. Am. J. Hum. Genet. *106*, 535–548.
82. Newman, A.M., Steen, C.B., Liu, C.L., Gentles, A.J., Chaudhuri, A.A., Scherer, F., Khodadoust, M.S., Esfahani, M.S., Luca, B.A., Steiner, D., et al. (2019). Determining cell type abundance and expression from bulk tissues with digital cytometry. Nat. Biotechnol. *37*, 773–782.
83. Newman, A.M., Liu, C.L., Green, M.R., Gentles, A.J., Feng, W., Xu, Y., Hoang, C.D., Diehn, M., and Alizadeh, A.A. (2015). Robust enumeration of cell subsets from tissue expression profiles. Nat. Methods *12*, 453–457.
84. Patro, R., Mount, S.M., and Kingsford, C. (2014). Sailfish enables alignment-free isoform quantification from RNA-seq reads using lightweight algorithms. Nat. Biotechnol. *32*, 462–464.
85. Koboldt, D.C., Zhang, Q., Larson, D.E., Shen, D., McLellan, M.D., Lin, L., Miller, C.A., Mardis, E.R., Ding, L., and Wilson, R.K. (2012). VarScan 2: somatic mutation and copy number

alteration discovery in cancer by exome sequencing. Genome Res. *22*, 568–576.
86. Fan, Y., Xi, L., Hughes, D.S.T., Zhang, J., Zhang, J., Futreal, P.A., Wheeler, D.A., and Wang, W. (2016). MuSE: accounting for tumor heterogeneity using a sample-specific error model improves sensitivity and specificity in mutation calling from sequencing data. Genome Biol. *17*, 178.
87. Cibulskis, K., Lawrence, M.S., Carter, S.L., Sivachenko, A., Jaffe, D., Sougnez, C., Gabriel, S., Meyerson, M., Lander, E.S., and Getz, G. (2013). Sensitive detection of somatic point mutations in impure and heterogeneous cancer samples. Nat. Biotechnol. *31*, 213–219.
88. Larson, D.E., Harris, C.C., Chen, K., Koboldt, D.C., Abbott, T.E., Dooling, D.J., Ley, T.J., Mardis, E.R., Wilson, R.K., and Ding, L. (2012). SomaticSniper: identification of somatic point mutations in whole genome sequencing data. Bioinformatics *28*, 311–317.
89. Hodis, E., Watson, I.R., Kryukov, G.V., Arold, S.T., Imielinski, M., Theurillat, J.-P., Nickerson, E., Auclair, D., Li, L., Place, C., et al. (2012). A landscape of driver mutations in melanoma. Cell *150*, 251–263.
90. Cancer Genome Atlas Network (2015). Genomic Classification of Cutaneous Melanoma. Cell *161*, 1681–1696.
91. Conway, J.R., Dietlein, F., Taylor-Weiner, A., AlDubayan, S., Vokes, N., Keenan, T., Reardon, B., He, M.X., Margolis, C.A., Weirather, J.L., et al. (2020). Integrated molecular drivers coordinate biological and clinical states in melanoma. Nat. Genet. *52*, 1373–1383.
92. Carter, H., Chen, S., Isik, L., Tyekucheva, S., Velculescu, V.E., Kinzler, K.W., Vogelstein, B., and Karchin, R. (2009). Cancer-specific high-throughput annotation of somatic mutations: computational prediction of driver missense mutations. Cancer Res. *69*, 6660–6667.
93. Tokheim, C., and Karchin, R. (2019). CHASMplus Reveals the Scope of Somatic Missense Mutations Driving Human Cancers. Cell Syst. *9*, 9–23.e8.
94. Lin, Y., Ghazanfar, S., Wang, K.Y.X., Gagnon-Bartsch, J.A., Lo, K.K., Su, X., Han, Z.-G., Ormerod, J.T., Speed, T.P., Yang, P., and Yang, J.Y.H. (2019). scMerge leverages factor analysis, stable expression, and pseudoreplication to merge multiple single-cell RNA-seq datasets. Proc. Natl. Acad. Sci. USA *116*, 9775–9784.
95. Mi, H., Ebert, D., Muruganujan, A., Mills, C., Albou, L.-P., Mushayamaha, T., and Thomas, P.D. (2021). PANTHER version 16: a revised family classification, tree-based classification tool, enhancer regions and extensive API. Nucleic Acids Res. *49*, D394–D403.
96. Jassal, B., Matthews, L., Viteri, G., Gong, C., Lorente, P., Fabregat, A., Sidiropoulos, K., Cook, J., Gillespie, M., Haw, R., et al. (2020). The reactome pathway knowledgebase. Nucleic Acids Res. *48*, D498–D503.
97. Aguiar, V.R.C., Masotti, C., Camargo, A.A., and Meyer, D. (2020). HLApers: HLA Typing and Quantification of Expression with Personalized Index. In Bioinformatics for Cancer Immunotherapy: Methods and Protocols, S. Boegel, ed. (Springer US), pp. 101–112.
98. Bray, N.L., Pimentel, H., Melsted, P., and Pachter, L. (2016). Near-optimal probabilistic RNA-seq quantification. Nat. Biotechnol. *34*, 525–527.
99. Frankish, A., Diekhans, M., Ferreira, A.-M., Johnson, R., Jungreis, I., Loveland, J., Mudge, J.M., Sisu, C., Wright, J., Armstrong, J., et al. (2019). GENCODE reference annotation for the human and mouse genomes. Nucleic Acids Res. *47*, D766–D773.
100. Robinson, J., Barker, D.J., Georgiou, X., Cooper, M.A., Flicek, P., and Marsh, S.G.E. (2020). IPD-IMGT/HLA Database. Nucleic Acids Res. *48*, D948–D955.
101. Love, M.I., Huber, W., and Anders, S. (2014). Moderated estimation of fold change and dispersion for RNA-seq data with DESeq2. Genome Biol. *15*, 550.
102. Aran, D., Sirota, M., and Butte, A.J. (2015). Systematic pancancer analysis of tumour purity. Nat. Commun. *6*, 8971.
103. Pérez-Guijarro, E., Yang, H.H., Araya, R.E., El Meskini, R., Michael, H.T., Vodnala, S.K., Marie, K.L., Smith, C., Chin, S., Lam, K.C., et al. (2020). Multimodel preclinical platform predicts clinical response of melanoma to immunotherapy. Nat. Med. *26*, 781–791.
104. Jiang, P., Gu, S., Pan, D., Fu, J., Sahu, A., Hu, X., Li, Z., Traugh, N., Bu, X., Li, B., et al. (2018). Signatures of T cell dysfunction and exclusion predict cancer immunotherapy response. Nat. Med. *24*, 1550–1558.
105. Reynisson, B., Alvarez, B., Paul, S., Peters, B., and Nielsen, M. (2020). NetMHCpan-4.1 and NetMHCIIpan-4.0: improved predictions of MHC antigen presentation by concurrent motif deconvolution and integration of MS MHC eluted ligand data. Nucleic Acids Res. *48*, W449–W454.
106. Purcell, S., Neale, B., Todd-Brown, K., Thomas, L., Ferreira, M.A.R., Bender, D., Maller, J., Sklar, P., de Bakker, P.I.W., Daly, M.J., and Sham, P.C. (2007). PLINK: a tool set for whole-genome association and population-based linkage analyses. Am. J. Hum. Genet. *81*, 559–575.
107. Chang, C.C., Chow, C.C., Tellier, L.C., Vattikuti, S., Purcell, S.M., and Lee, J.J. (2015). Second-generation PLINK: rising to the challenge of larger and richer datasets. GigaScience *4*, 7.
108. Armitage, P., and Doll, R. (1954). The age distribution of cancer and a multi-stage theory of carcinogenesis. Br. J. Cancer *8*, 1–12.
109. Moolgavkar, S.H., and Knudson, A.G., Jr. (1981). Mutation and cancer: a model for human carcinogenesis. J. Natl. Cancer Inst. *66*, 1037–1052.
110. Moolgavkar, S.H., Dewanji, A., and Venzon, D.J. (1988). A stochastic two-stage model for cancer risk assessment. I. The hazard function and the probability of tumor. Risk Anal. *8*, 383–392.
111. Moolgavkar, S.H., and Luebeck, G. (1990). Two-event model for carcinogenesis: biological, mathematical, and statistical considerations. Risk Anal. *10*, 323–341.
112. Brouwer, A.F., Meza, R., and Eisenberg, M.C. (2017). Parameter estimation for multistage clonal expansion models from cancer incidence data: A practical identifiability analysis. PLoS Comput. Biol. *13*, e1005431.
113. Meza, R., Jeon, J., Moolgavkar, S.H., and Luebeck, E.G. (2008). Age-specific incidence of cancer: Phases, transitions, and biological implications. Proc. Natl. Acad. Sci. USA *105*, 16284–16289.
114. Tan, W.-Y. (2020). Stochastic Models of Carcinogenesis (CRC Press).
115. SEER Research Data 1975-2018 when Using SEER*Stat: Surveillance, Epidemiology, and End Results (SEER) Program (www.seer.cancer.gov) SEER*Stat Database: Incidence - SEER Research Data, 9 Registries, Nov 2020 Sub (1975-2018) - Linked To County Attributes - Time Dependent (1990-2018) Income/Rurality, 1969-2019 Counties, National

Cancer Institute, DCCPS, Surveillance Research Program, released April 2021, based on the November 2020 submission.
116. Luebeck, E.G., Curtius, K., Jeon, J., and Hazelton, W.D. (2013). Impact of tumor progression on cancer incidence curves. Cancer Res. *73*, 1086–1096.
117. Luebeck, G.E., Vaughan, T.L., Curtius, K., and Hazelton, W.D. (2021). Modeling historic incidence trends implies early field cancerization in esophageal squamous cell carcinoma. PLoS Comput. Biol. *17*, e1008961.
118. Gerstung, M., Jolly, C., Leshchiner, I., Dentro, S.C., Gonzalez, S., Rosebrock, D., Mitchell, T.J., Rubanova, Y., Anur, P., Yu, K., et al. (2020). The evolutionary history of 2,658 cancers. Nature *578*, 122–128.
119. Alexandrov, L.B., Jones, P.H., Wedge, D.C., Sale, J.E., Campbell, P.J., Nik-Zainal, S., and Stratton, M.R. (2015). Clock-like mutational processes in human somatic cells. Nat. Genet. *47*, 1402–1407.
120. Steele, C.D., Abbasi, A., Islam, S.M.A., Bowes, A.L., Khandekar, A., Haase, K., Hames-Fathi, S., Ajayi, D., Verfaillie, A., Dhami, P., et al. (2022). Signatures of copy number alterations in human cancer. Nature *606*, 984–991.
121. Favero, F., Joshi, T., Marquard, A.M., Birkbak, N.J., Krzystanek, M., Li, Q., Szallasi, Z., and Eklund, A.C. (2015). Sequenza: allele-specific copy number and mutation profiles from tumor sequencing data. Ann. Oncol. *26*, 64–70.
122. Seabold, S., and Perktold, J. (2010). Statsmodels: Econometric and statistical modeling with python. In Proceedings of the 9th Python in Science Conference, p. 61.
123. Okada, Y., Han, B., Tsoi, L.C., Stuart, P.E., Ellinghaus, E., Tejasvi, T., Chandran, V., Pellett, F., Pollock, R., Bowcock, A.M., et al. (2014). Fine mapping major histocompatibility complex associations in psoriasis and its clinical subtypes. Am. J. Hum. Genet. *95*, 162–172.
124. Chen, H., Hayashi, G., Lai, O.Y., Dilthey, A., Kuebler, P.J., Wong, T.V., Martin, M.P., Fernandez Vina, M.A., McVean, G., Wabl, M., et al. (2012). Psoriasis patients are enriched for genetic variants that protect against HIV-1 disease. PLoS Genet. *8*, e1002514.
125. Helms, C., Saccone, N.L., Cao, L., Daw, J.A.W., Cao, K., Hsu, T.M., Taillon-Miller, P., Duan, S., Gordon, D., Pierce, B., et al. (2005). Localization of PSORS1 to a haplotype block harboring HLA-C and distinct from corneodesmosin and HCR. Hum. Genet. *118*, 466–476.
126. Winchester, R., Minevich, G., Steshenko, V., Kirby, B., Kane, D., Greenberg, D.A., and FitzGerald, O. (2012). HLA associations reveal genetic heterogeneity in psoriatic arthritis and in the psoriasis phenotype. Arthritis Rheum. *64*, 1134–1144.
127. FitzGerald, O., Haroon, M., Giles, J.T., and Winchester, R. (2015). Concepts of pathogenesis in psoriatic arthritis: genotype determines clinical phenotype. Arthritis Res. Ther. *17*, 115.
128. Ogg, G.S., Rod Dunbar, P., Romero, P., Chen, J.L., and Cerundolo, V. (1998). High frequency of skin-homing melanocyte-specific cytotoxic T lymphocytes in autoimmune vitiligo. J. Exp. Med. *188*, 1203–1208.
129. Lang, K.S., Caroli, C.C., Muhm, A., Wernet, D., Moris, A., Schittek, B., Knauss-Scherwitz, E., Stevanovic, S., Rammensee, H.G., and Garbe, C. (2001). HLA-A2 restricted, melanocyte-specific CD8(+) T lymphocytes detected in vitiligo patients are related to disease activity and are predominantly directed against MelanA/MART1. J. Invest. Dermatol. *116*, 891–897.
130. Palermo, B., Campanelli, R., Garbelli, S., Mantovani, S., Lantelme, E., Brazzelli, V., Ardigó, M., Borroni, G., Martinetti, M., Badulli, C., et al. (2001). Specific cytotoxic T lymphocyte responses against Melan-A/MART1, tyrosinase and gp100 in vitiligo by the use of major histocompatibility complex/peptide tetramers: the role of cellular immunity in the etiopathogenesis of vitiligo. J. Invest. Dermatol. *117*, 326–332.
131. Jin, Y., Ferrara, T., Gowan, K., Holcomb, C., Rastrou, M., Erlich, H.A., Fain, P.R., and Spritz, R.A. (2012). Next-generation DNA re-sequencing identifies common variants of TYR and HLA-A that modulate the risk of generalized vitiligo via antigen presentation. J. Invest. Dermatol. *132*, 1730–1733.
132. Zhang, X.-J., Liu, H.-S., Liang, Y.-H., Sun, L.-D., Wang, J.-Y., Yang, S., Liu, J.-B., Gao, M., He, P.-P., Cui, Y., and Yang, Q. (2004). Association of HLA class I alleles with vitiligo in Chinese Hans. J. Dermatol. Sci. *35*, 165–168.
133. Quan, C., Ren, Y.-Q., Xiang, L.-H., Sun, L.-D., Xu, A.-E., Gao, X.-H., Chen, H.-D., Pu, X.-M., Wu, R.-N., Liang, C.-Z., et al. (2010). Genome-wide association study for vitiligo identifies susceptibility loci at 6q27 and the MHC. Nat. Genet. *42*, 614–618.
134. Nair, R.P., Stuart, P.E., Nistor, I., Hiremagalore, R., Chia, N.V.C., Jenisch, S., Weichenthal, M., Abecasis, G.R., Lim, H.W., Christophers, E., et al. (2006). Sequence and haplotype analysis supports HLA-C as the psoriasis susceptibility 1 gene. Am. J. Hum. Genet. *78*, 827–851.
135. Gudjonsson, J.E., Karason, A., Antonsdottir, A., Runarsdottir, E.H., Hauksson, V.B., Upmanyu, R., Gulcher, J., Stefansson, K., and Valdimarsson, H. (2003). Psoriasis patients who are homozygous for the HLA-Cw*0602 allele have a 2.5-fold increased risk of developing psoriasis compared with Cw6 heterozygotes. Br. J. Dermatol. *148*, 233–235.
136. Yamamoto, T., Yokozeki, H., and Nishioka, K. (2005). Psoriasis arthropathy and HLA-B51: report of 5 cases. J. Dermatol. *32*, 606–610.
137. Nejentsev, S., Howson, J.M.M., Walker, N.M., Szeszko, J., Field, S.F., Stevens, H.E., Reynolds, P., Hardy, M., King, E., Masters, J., et al. (2007). Localization of type 1 diabetes susceptibility to the MHC class I genes HLA-B and HLA-A. Nature *450*, 887–892.
138. Noble, J.A., Valdes, A.M., Varney, M.D., Carlson, J.A., Moonsamy, P., Fear, A.L., Lane, J.A., Lavant, E., Rappner, R., Louey, A., et al. (2010). HLA class I and genetic susceptibility to type 1 diabetes: results from the Type 1 Diabetes Genetics Consortium. Diabetes *59*, 2972–2979.
139. Howson, J.M.M., Walker, N.M., Clayton, D., Todd, J.A.; and Type 1 Diabetes Genetics Consortium (2009). Confirmation of HLA class II independent type 1 diabetes associations in the major histocompatibility complex including HLA-B and HLA-A. Diabetes Obes. Metab. *11*, 31–45.
140. Ohno, S., Ohguchi, M., Hirose, S., Matsuda, H., Wakisaka, A., and Aizawa, M. (1982). Close Association of HLA-Bw51 With Behcet's Disease. Arch. Ophthalmol. *100*, 1455–1458.
141. Kaufman, J. (2018). Generalists and Specialists: A New View of How MHC Class I Molecules Fight Infectious Pathogens. Trends Immunol. *39*, 367–379.
142. Bashirova, A.A., Viard, M., Naranbhai, V., Grifoni, A., Garcia-Beltran, W., Akdag, M., Yuki, Y., Gao, X., O'hUigin, C.,

Raghavan, M., et al. (2020). HLA tapasin independence: broader peptide repertoire and HIV control. Proc. Natl. Acad. Sci. USA *117*, 28232–28238.
143. Gonzalez-Galarza, F.F., McCabe, A., Santos, E.J.M.D., Jones, J., Takeshita, L., Ortega-Rivera, N.D., Cid-Pavon, G.M.D., Ramsbottom, K., Ghattaoraya, G., Alfirevic, A., et al. (2020). Allele frequency net database (AFND) 2020 update: gold-standard data classification, open access genotype data and new query tools. Nucleic Acids Res. *48*, D783–D788.
144. Magnus, K. (1977). Prognosis in malignant melanoma of the skin. Significance of stage of disease, anatomical site, sex, age and period of diagnosis. Cancer *40*, 389–397.
145. Natale, C.A., Li, J., Zhang, J., Dahal, A., Dentchev, T., Stanger, B.Z., and Ridky, T.W. (2018). Activation of G protein-coupled estrogen receptor signaling inhibits melanoma and improves response to immune checkpoint blockade. Elife *7*, e31770.
146. Elahi, S., Dinges, W.L., Lejarcegui, N., Laing, K.J., Collier, A.C., Koelle, D.M., McElrath, M.J., and Horton, H. (2011). Protective HIV-specific CD8+ T cells evade Treg cell suppression. Nat. Med. *17*, 989–995.
147. Wolfe, D., Dudek, S., Ritchie, M.D., and Pendergrass, S.A. (2013). Visualizing genomic information across chromosomes with PhenoGram. BioData Min. *6*, 18.
148. Gaziano, J.M., Concato, J., Brophy, M., Fiore, L., Pyarajan, S., Breeling, J., Whitbourne, S., Deen, J., Shannon, C., Humphries, D., et al. (2016). Million Veteran Program: A mega-biobank to study genetic influences on health and disease. J. Clin. Epidemiol. *70*, 214–223.
149. Carlson, J.A. (2003). Tumor doubling time of cutaneous melanoma and its metastasis. Am. J. Dermatopathol. *25*, 291–299.
150. Kay, K., Dolcy, K., Bies, R., and Shah, D.K. (2019). Estimation of Solid Tumor Doubling Times from Progression-Free Survival Plots Using a Novel Statistical Approach. AAPS J. *21*, 27.
151. Teulings, H.-E., Willemsen, K.J., Glykofridis, I., Krebbers, G., Komen, L., Kroon, M.W., Kemp, E.H., Wolkerstorfer, A., van der Veen, J.P.W., Luiten, R.M., and Tjin, E.P.M. (2014). The antibody response against MART-1 differs in patients with melanoma-associated leucoderma and vitiligo. Pigment Cell Melanoma Res. *27*, 1086–1096.
152. Rodrigues, M., Ezzedine, K., Hamzavi, I., Pandya, A.G., Harris, J.E.; and Vitiligo Working Group (2017). New discoveries in the pathogenesis and classification of vitiligo. J. Am. Acad. Dermatol. *77*, 1–13.
153. Kawakami, Y., Eliyahu, S., Delgado, C.H., Robbins, P.F., Sakaguchi, K., Appella, E., Yannelli, J.R., Adema, G.J., Miki, T., and Rosenberg, S.A. (1994). Identification of a human melanoma antigen recognized by tumor-infiltrating lymphocytes associated with in vivo tumor rejection. Proc. Natl. Acad. Sci. USA *91*, 6458–6462.
154. Chen, Y.T., Stockert, E., Tsang, S., Coplan, K.A., and Old, L.J. (1995). Immunophenotyping of melanomas for tyrosinase: implications for vaccine development. Proc. Natl. Acad. Sci. USA *92*, 8125–8129.
155. Wang, R.F., Appella, E., Kawakami, Y., Kang, X., and Rosenberg, S.A. (1996). Identification of TRP-2 as a human tumor antigen recognized by cytotoxic T lymphocytes. J. Exp. Med. *184*, 2207–2216.
156. Brasseur, F., Rimoldi, D., Liénard, D., Lethé, B., Carrel, S., Arienti, F., Suter, L., Vanwijck, R., Bourlond, A., and Humblet, Y. (1995). Expression of MAGE genes in primary and metastatic cutaneous melanoma. Int. J. Cancer *63*, 375–380.
157. Mori, M., Inoue, H., Mimori, K., Shibuta, K., Baba, K., Nakashima, H., Haraguchi, M., Tsuji, K., Ueo, H., Barnard, G.F., and Akiyoshi, T. (1996). Expression of MAGE genes in human colorectal carcinoma. Ann. Surg. *224*, 183–188.
158. Scarcella, D.L., Chow, C.W., Gonzales, M.F., Economou, C., Brasseur, F., and Ashley, D.M. (1999). Expression of MAGE and GAGE in high-grade brain tumors: a potential target for specific immunotherapy and diagnostic markers. Clin. Cancer Res. *5*, 335–341.
159. Tajima, K., Obata, Y., Tamaki, H., Yoshida, M., Chen, Y.-T., Scanlan, M.J., Old, L.J., Kuwano, H., Takahashi, T., Takahashi, T., and Mitsudomi, T. (2003). Expression of cancer/testis (CT) antigens in lung cancer. Lung Cancer *42*, 23–33.
160. Gure, A.O., Chua, R., Williamson, B., Gonen, M., Ferrera, C.A., Gnjatic, S., Ritter, G., Simpson, A.J.G., Chen, Y.-T., Old, L.J., and Altorki, N.K. (2005). Cancer-testis genes are coordinately expressed and are markers of poor outcome in non-small cell lung cancer. Clin. Cancer Res. *11*, 8055–8062.
161. Barrow, C., Browning, J., MacGregor, D., Davis, I.D., Sturrock, S., Jungbluth, A.A., and Cebon, J. (2006). Tumor antigen expression in melanoma varies according to antigen and stage. Clin. Cancer Res. *12*, 764–771.
162. Kim, Y.-D., Park, H.-R., Song, M.-H., Shin, D.-H., Lee, C.-H., Lee, M.-K., and Lee, S.-Y. (2012). Pattern of cancer/testis antigen expression in lung cancer patients. Int. J. Mol. Med. *29*, 656–662.
163. Nielsen, M., and Andreatta, M. (2016). NetMHCpan-3.0; improved prediction of binding to MHC class I molecules integrating information from multiple receptor and peptide length datasets. Genome Med. *8*, 33.
164. Andersen, M.H., Fensterle, J., Ugurel, S., Reker, S., Houben, R., Guldberg, P., Berger, T.G., Schadendorf, D., Trefzer, U., Bröcker, E.B., et al. (2004). Immunogenicity of constitutively active V599EBRaf. Cancer Res. *64*, 5456–5460.
165. Bradley, S.D., Chen, Z., Melendez, B., Talukder, A., Khalili, J.S., Rodriguez-Cruz, T., Liu, S., Whittington, M., Deng, W., Li, F., et al. (2015). BRAFV600E Co-opts a Conserved MHC Class I Internalization Pathway to Diminish Antigen Presentation and CD8+ T-cell Recognition of Melanoma. Cancer Immunol. Res. *3*, 602–609.
166. Williams, J.D., Jacobson, E.L., Kim, H., Kim, M., and Jacobson, M.K. (2012). Folate in Skin Cancer Prevention. Subcell. Biochem. *56*, 181–197.
167. Simpson, A.J.G., Caballero, O.L., Jungbluth, A., Chen, Y.-T., and Old, L.J. (2005). Cancer/testis antigens, gametogenesis and cancer. Nat. Rev. Cancer *5*, 615–625.
168. Fang, D., Hallman, J., Sangha, N., Kute, T.E., Hammarback, J.A., White, W.L., and Setaluri, V. (2001). Expression of Microtubule-Associated Protein 2 in Benign and Malignant Melanocytes. Am. J. Pathol. *158*, 2107–2115.
169. Vachtenheim, J., Novotna, H., and Ghanem, G. (2001). Transcriptional Repression of the Microphthalmia Gene in Melanoma Cells Correlates with the Unresponsiveness of Target Genes to Ectopic Microphthalmia-Associated Transcription Factor. J. Invest. Dermatol. *117*, 1505–1511.
170. Marcu, A., Bichmann, L., Kuchenbecker, L., Kowalewski, D.J., Freudenmann, L.K., Backert, L., Mühlenbruch, L., Szolek, A., Lübke, M., Wagner, P., et al. (2021). HLA Ligand Atlas: a benign reference of HLA-presented peptides to improve

T-cell-based cancer immunotherapy. J. Immunother. Cancer *9*, e002071.
171. Aris, M., Zubieta, M.R., Colombo, M., Arriaga, J.M., Bianchini, M., Alperovich, M., Bravo, A.I., Barrio, M.M., and Mordoh, J. (2012). MART-1- and gp100-Expressing and -Non-Expressing Melanoma Cells Are Equally Proliferative in Tumors and Clonogenic In Vitro. J. Invest. Dermatol. *132*, 365–374.
172. Journe, F., Id Boufker, H., Van Kempen, L., Galibert, M.-D., Wiedig, M., Salès, F., Theunis, A., Nonclercq, D., Frau, A., Laurent, G., et al. (2011). TYRP1 mRNA expression in melanoma metastases correlates with clinical outcome. Br. J. Cancer *105*, 1726–1732.
173. Wieczorek, M., Abualrous, E.T., Sticht, J., Álvaro-Benito, M., Stolzenberg, S., Noé, F., and Freund, C. (2017). Major Histocompatibility Complex (MHC) Class I and MHC Class II Proteins: Conformational Plasticity in Antigen Presentation. Front. Immunol. *8*, 292.
174. Bodis, G., Toth, V., and Schwarting, A. (2018). Role of Human Leukocyte Antigens (HLA) in Autoimmune Diseases. Rheumatol. Ther. *5*, 5–20.
175. Miyadera, H., Ohashi, J., Lernmark, Å., Kitamura, T., and Tokunaga, K. (2015). Cell-surface MHC density profiling reveals instability of autoimmunity-associated HLA. J. Clin. Invest. *125*, 275–291.
176. Yang, R.-Y., Rabinovich, G.A., and Liu, F.-T. (2008). Galectins: structure, function and therapeutic potential. Expert Rev. Mol. Med. *10*, e17.
177. Crux, N.B., and Elahi, S. (2017). Human Leukocyte Antigen (HLA) and Immune Regulation: How Do Classical and Non-Classical HLA Alleles Modulate Immune Response to Human Immunodeficiency Virus and Hepatitis C Virus Infections? Front. Immunol. *8*, 832.
178. Wang, F., He, W., Zhou, H., Yuan, J., Wu, K., Xu, L., and Chen, Z.K. (2007). The Tim-3 ligand galectin-9 negatively regulates CD8+ alloreactive T cell and prolongs survival of skin graft. Cell. Immunol. *250*, 68–74.
179. Migueles, S.A., Osborne, C.M., Royce, C., Compton, A.A., Joshi, R.P., Weeks, K.A., Rood, J.E., Berkley, A.M., Sacha, J.B., Cogliano-Shutta, N.A., et al. (2008). Lytic granule loading of CD8+ T cells is required for HIV-infected cell elimination associated with immune control. Immunity *29*, 1009–1021.
180. Košmrlj, A., Read, E.L., Qi, Y., Allen, T.M., Altfeld, M., Deeks, S.G., Pereyra, F., Carrington, M., Walker, B.D., and Chakraborty, A.K. (2010). Effects of thymic selection of the T-cell repertoire on HLA class I-associated control of HIV infection. Nature *465*, 350–354.
181. Chappell, P., Meziane, E.K., Harrison, M., Magiera, Ł., Hermann, C., Mears, L., Wrobel, A.G., Durant, C., Nielsen, L.L., Buus, S., et al. (2015). Expression levels of MHC class I molecules are inversely correlated with promiscuity of peptide binding. Elife *4*, e05345.
182. International HIV Controllers Study, Pereyra, F., Jia, X., McLaren, P.J., Telenti, A., de Bakker, P.I.W., Walker, B.D., Ripke, S., Brumme, C.J., Pulit, S.L., et al. (2010). The major genetic determinants of HIV-1 control affect HLA class I peptide presentation. Science *330*, 1551–1557.
183. Zhang, Y., Peng, Y., Yan, H., Xu, K., Saito, M., Wu, H., Chen, X., Ranasinghe, S., Kuse, N., Powell, T., et al. (2011). Multilayered defense in HLA-B51-associated HIV viral control. J. Immunol. *187*, 684–691.
184. Mobbs, J.I., Illing, P.T., Dudek, N.L., Brooks, A.G., Baker, D.G., Purcell, A.W., Rossjohn, J., and Vivian, J.P. (2017). The molecular basis for peptide repertoire selection in the human leucocyte antigen (HLA) C*06:02 molecule. J. Biol. Chem. *292*, 17203–17215.
185. Madden, D.R., Gorga, J.C., Strominger, J.L., and Wiley, D.C. (1991). The structure of HLA-B27 reveals nonamer self-peptides bound in an extended conformation. Nature *353*, 321–325.
186. Saveanu, L., Carroll, O., Lindo, V., Del Val, M., Lopez, D., Lepelletier, Y., Greer, F., Schomburg, L., Fruci, D., Niedermann, G., and van Endert, P.M. (2005). Concerted peptide trimming by human ERAP1 and ERAP2 aminopeptidase complexes in the endoplasmic reticulum. Nat. Immunol. *6*, 689–697.
187. Saric, T., Chang, S.-C., Hattori, A., York, I.A., Markant, S., Rock, K.L., Tsujimoto, M., and Goldberg, A.L. (2002). An IFN-γ–induced aminopeptidase in the ER, ERAP1, trims precursors to MHC class I–presented peptides. Nat. Immunol. *3*, 1169–1176.
188. York, I.A., Chang, S.-C., Saric, T., Keys, J.A., Favreau, J.M., Goldberg, A.L., and Rock, K.L. (2002). The ER aminopeptidase ERAP1 enhances or limits antigen presentation by trimming epitopes to 8-9 residues. Nat. Immunol. *3*, 1177–1184.
189. Hammer, G.E., Gonzalez, F., James, E., Nolla, H., and Shastri, N. (2007). In the absence of aminopeptidase ERAAP, MHC class I molecules present many unstable and highly immunogenic peptides. Nat. Immunol. *8*, 101–108.
190. Reeves, E., Edwards, C.J., Elliott, T., and James, E. (2013). Naturally occurring ERAP1 haplotypes encode functionally distinct alleles with fine substrate specificity. J. Immunol. *191*, 35–43.
191. Martín-Esteban, A., Gómez-Molina, P., Sanz-Bravo, A., and López de Castro, J.A. (2014). Combined effects of ankylosing spondylitis-associated ERAP1 polymorphisms outside the catalytic and peptide-binding sites on the processing of natural HLA-B27 ligands. J. Biol. Chem. *289*, 3978–3990.
192. Genetic Analysis of Psoriasis Consortium & the Wellcome Trust Case Control Consortium 2, Strange, A., Capon, F., Spencer, C.C.A., Knight, J., Weale, M.E., Allen, M.H., Barton, A., Band, G., Bellenguez, C., et al. (2010). A genome-wide association study identifies new psoriasis susceptibility loci and an interaction between HLA-C and ERAP1. Nat. Genet. *42*, 985–990.
193. Tsoi, L.C., Spain, S.L., Knight, J., Ellinghaus, E., Stuart, P.E., Capon, F., Ding, J., Li, Y., Tejasvi, T., Gudjonsson, J.E., et al. (2012). Identification of 15 new psoriasis susceptibility loci highlights the role of innate immunity. Nat. Genet. *44*, 1341–1348.
194. Yin, X., Low, H.Q., Wang, L., Li, Y., Ellinghaus, E., Han, J., Estivill, X., Sun, L., Zuo, X., Shen, C., et al. (2015). Genome-wide meta-analysis identifies multiple novel associations and ethnic heterogeneity of psoriasis susceptibility. Nat. Commun. *6*, 6916–7011.
195. Cummings, A.L., Gukasyan, J., Lu, H.Y., Grogan, T., Sunga, G., Fares, C.M., Hornstein, N., Zaretsky, J., Carroll, J., Bachrach, B., et al. (2020). Mutational landscape influences immunotherapy outcomes among patients with non-small-cell lung cancer with human leukocyte antigen supertype B44. Nat. Cancer *1*, 1167–1175.
196. DiBrino, M., Parker, K.C., Margulies, D.H., Shiloach, J., Turner, R.V., Biddison, W.E., and Coligan, J.E. (1995).

Identification of the peptide binding motif for HLA-B44, one of the most common HLA-B alleles in the Caucasian population. Biochemistry *34*, 10130–10138.

197. Brewerton, D.A., Hart, F.D., Nicholls, A., Caffrey, M., James, D.C., and Sturrock, R.D. (1973). Ankylosing spondylitis and HL-A 27. Lancet *1*, 904–907.

198. Brewerton, D.A., Caffrey, M., Nicholls, A., Walters, D., and James, D.C. (1974). HL-A 27 and arthropathies associated with ulcerative colitis and psoriasis. Lancet *1*, 956–958.

199. Tam, L.-S., Gu, J., and Yu, D. (2010). Pathogenesis of ankylosing spondylitis. Nat. Rev. Rheumatol. *6*, 399–405.

The American Journal of Human Genetics, Volume *110*

Supplemental information

Autoimmune alleles at the major histocompatibility locus modify melanoma susceptibility

James V. Talwar, David Laub, Meghana S. Pagadala, Andrea Castro, McKenna Lewis, Georg E. Luebeck, Bryan R. Gorman, Cuiping Pan, Frederick N. Dong, Kyriacos Markianos, Craig C. Teerlink, Julie Lynch, Richard Hauger, Saiju Pyarajan, Philip S. Tsao, Gerald P. Morris, Rany M. Salem, Wesley K. Thompson, Kit Curtius, Maurizio Zanetti, and Hannah Carter

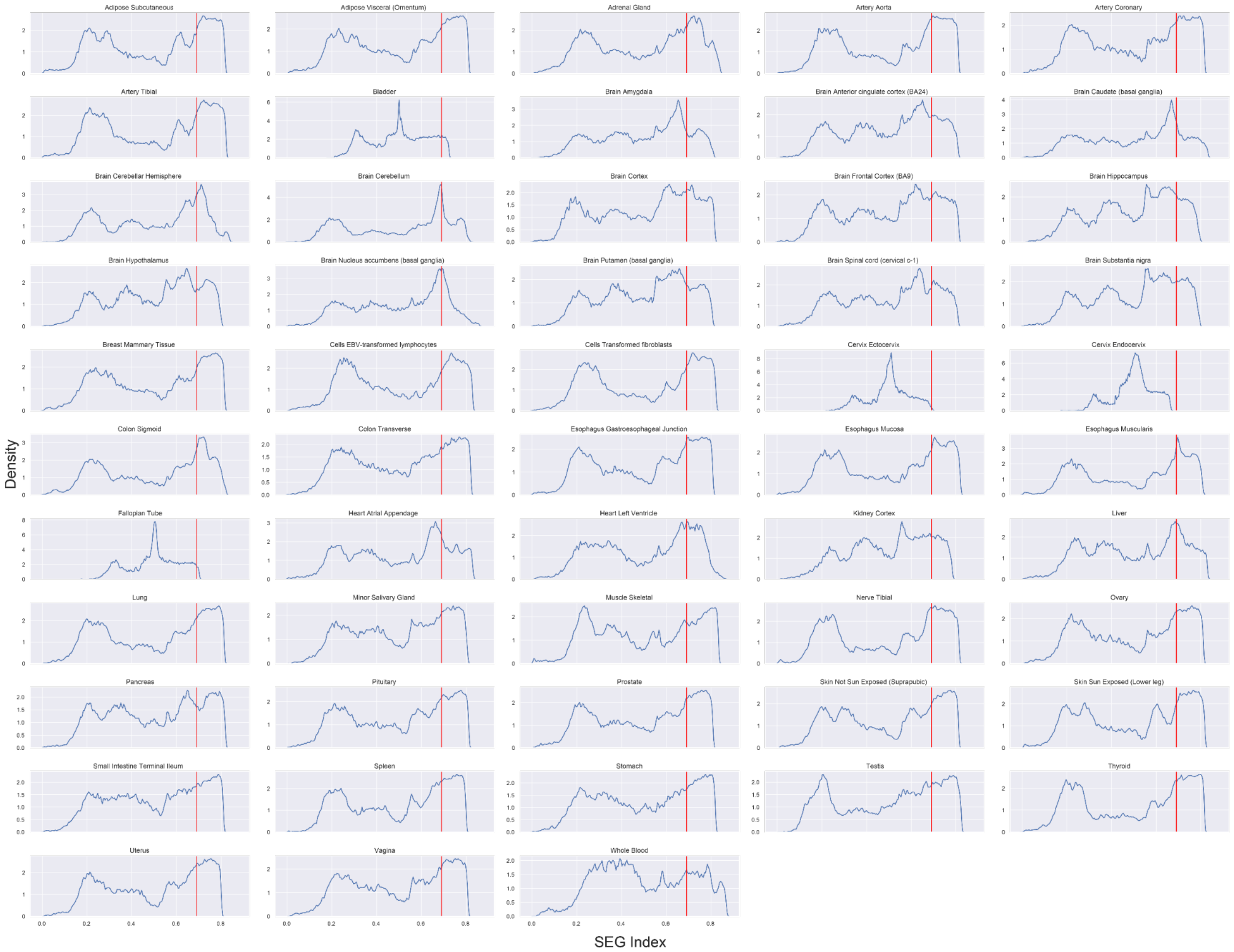

Figure S1: Distributions of stably expressed gene (SEG) scores shown for all 53 tissues in GTEx. The threshold of 0.69 was selected and is shown in red.

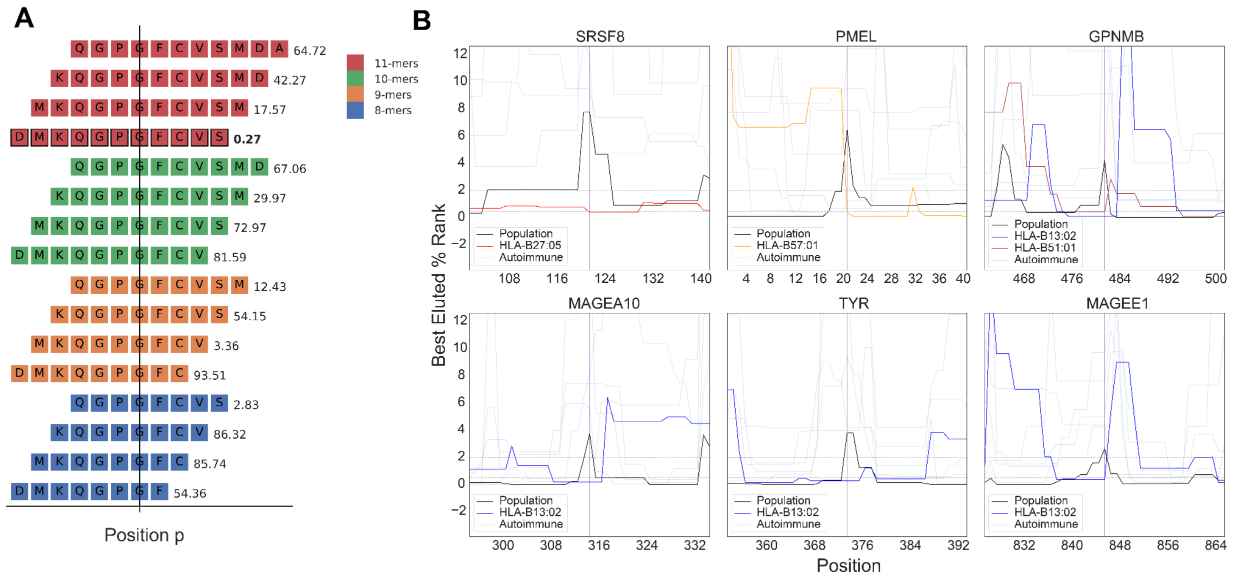

Figure S2: A) Position-wise best percentile rank scores were computed from predictions for 8-11mers by taking the best percentile rank of all overlapping peptides at any given position. B) Greatest position-wise differences in best percentile rank between HLA-B autoimmune and common alleles amongst the top 2 differences (*SRSF8* and the canonical melanocyte gene *PMEL*) and several DE conserved antigens (*GPNMB, MAGEA10, TYR, MAGEE1*). Common alleles are shown in black and autoimmune alleles are shown in transparent blue unless they are predicted to elute at better percentile ranks than common alleles. Vertical purple lines demarcate positions where autoimmune alleles are predicted to elute at better percentile ranks than common alleles. Plots show up to +/- 20 amino acids of the demarcated positions.

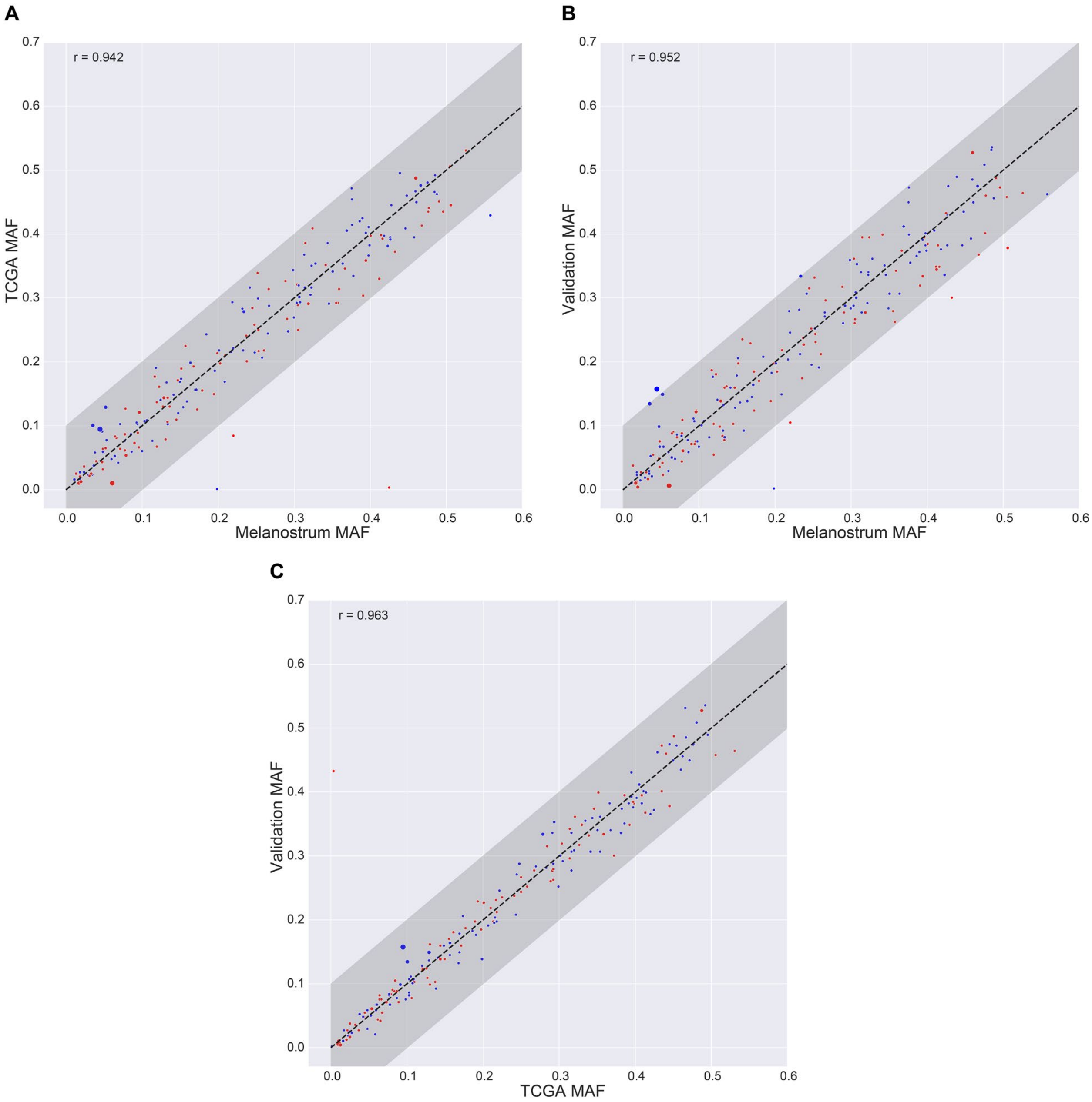

Figure S3: PRS SNP minor allele frequencies (MAFs) relationship between A) Discovery and Melanostrum (Pearson R = 0.942), B) Validation and Melanostrum (Pearson R = 0.952), and C) Validation and Discovery (Pearson R = 0.963). Red points are PRS SNPs with a protective effect in melanoma (negative PRS weight), while blue points are PRS SNPs with a predisposing melanoma effect (positive PRS weight). Points are sized according to the magnitude of their weight in the PRS. SNP effects ranged in PRS weight from -0.4 to 0.49.

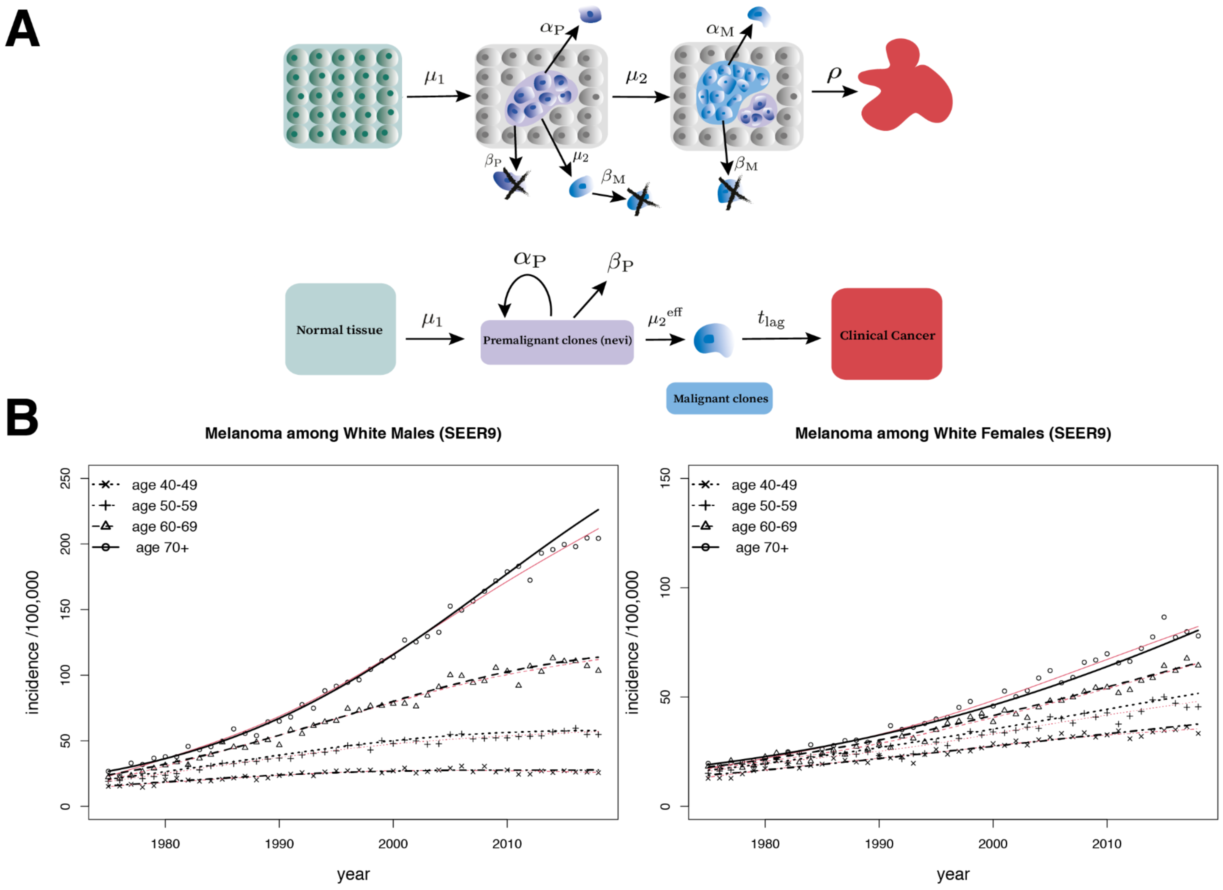

Figure S4: A) *Upper row*: Multistage clonal expansion (MSCE) model - illustration of a stochastic realization of the multi-type branching process. In the two-stage MSCE model, the first 'hit' occurs when normal cells (green) undergo asymmetric division (due to mutation, for example) and create one premalignant daughter cell with rate μ_1 and one normal cell. Premalignant cells can then clonally expand with rates α_P for division and β_P for cell death/differentiation (purple clones, e.g., dysplastic nevi). The second 'hit' occurs at rate μ_2 per cell per year wherein a premalignant cell creates a malignant daughter cell (due to secondary mutation, for example) and 1 premalignant cell. α_M and β_M represent cell division and death/differentiation, respectively, for malignant cells (blue). Clinical detection occurs for malignant clones with rate ρ. *Bottom row*: Mathematical approximation of the two-stage model includes an effective mutation rate μ_2^{eff} for transformation of a malignant cell that survives and a tumor sojourn time/ 'lag-time' representing the time between the first persistent malignant cell and the clinically detected cancer. B) Hazard curves from the MSCE models for melanoma incidence/100,000 (black lines) correspond to US SEER data from 1975-2018 (black shapes) in white females (right) and white males (left) separately by age groups, as represented by the different symbols, over calendar years. Calibrated parameters from the two-stage model yield similar average lag-time (tumor sojourn time) for males and females of ~10 years. For comparison, non-parametric fits using 4*th*-order smoothing splines shown in red.

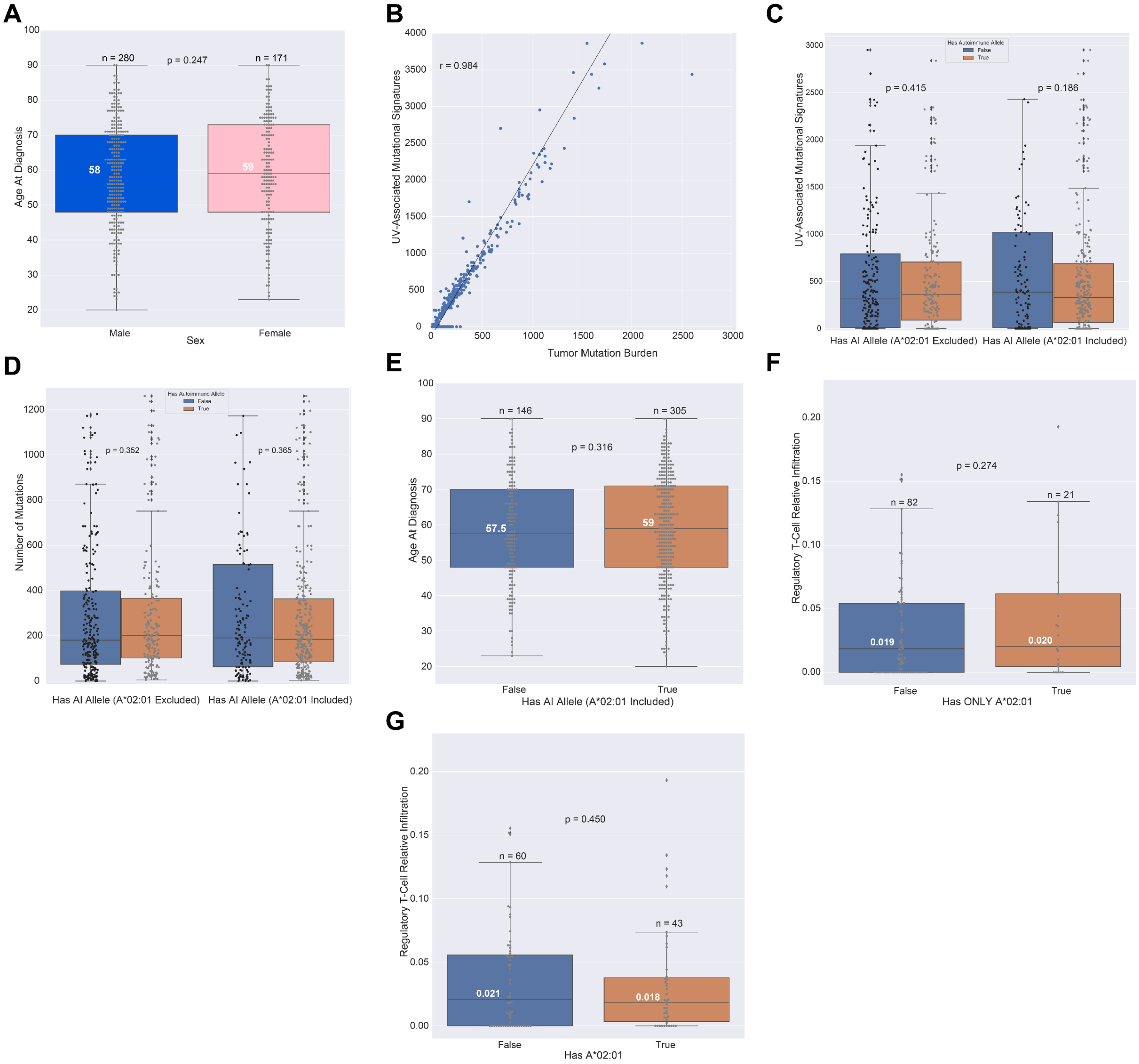

Figure S5: Covariates and associations with melanoma in the TCGA. A) Boxplots comparing sex and melanoma age of diagnosis. B) Correlation between total mutation burden and UV-signature associated mutations. C) Boxplots comparing the number of UV-signature associated mutations across those with and without a MHC-I linked autoimmune allele. D) Boxplots comparing the number of mutations across those with and without a MHC-I linked autoimmune allele. E) Effect of MHC-I autoimmune alleles on age at diagnosis in melanoma including HLA-A*02:01. F) Carriers of HLA-A*02:01 and no other autoimmune alleles do not exhibit a significant difference in regulatory T-cells in primary melanoma tumors. G) Carriers of HLA-A*02:01, independent of other alleles carried, do not exhibit a significant difference in regulatory T-cells in primary melanoma tumors.

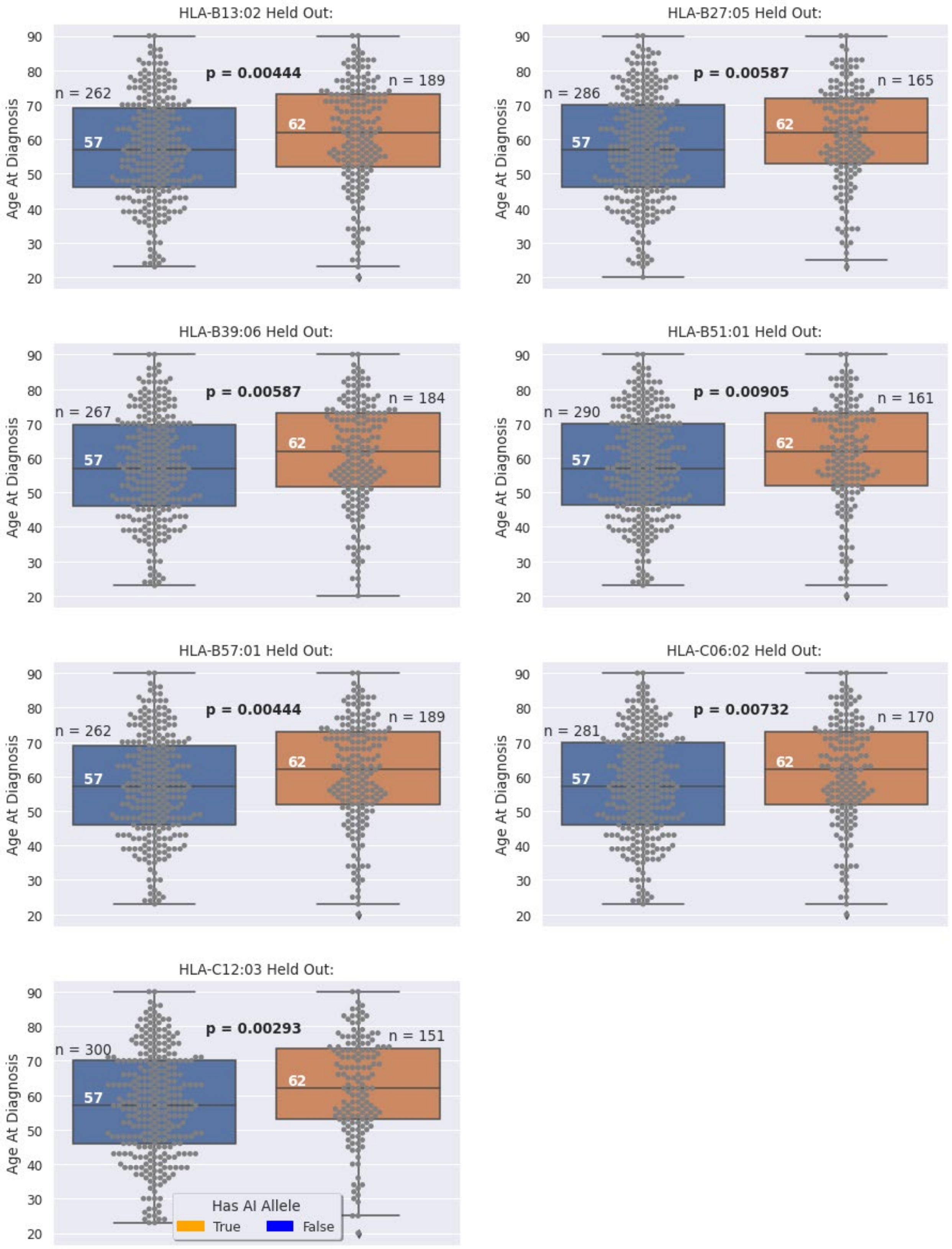
HLA-B13:02 Held Out:
p = 0.00444
n = 262
n = 189
57
62
HLA-B27:05 Held Out:
p = 0.00587
n = 286
n = 165
57
62
HLA-B39:06 Held Out:
p = 0.00587
n = 267
n = 184
57
62
HLA-B51:01 Held Out:
p = 0.00905
n = 290
n = 161
57
62
HLA-B57:01 Held Out:
p = 0.00444
n = 262
n = 189
57
62
HLA-C06:02 Held Out:
p = 0.00732
n = 281
n = 170
57
62
HLA-C12:03 Held Out:
p = 0.00293
n = 300
n = 151
57
62
Age At Diagnosis
20
30
40
50
60
70
80
90
Has AI Allele
True
False

Figure S6: Effect of omitting each of the 7 individual autoimmune alleles from carrier status in TCGA. For each autoimmune allele, individuals carrying only the excluded allele were assigned to non-AI allele carrier status. All p-values are multiple hypotheses corrected by means of the Benjamini-Hochberg method.

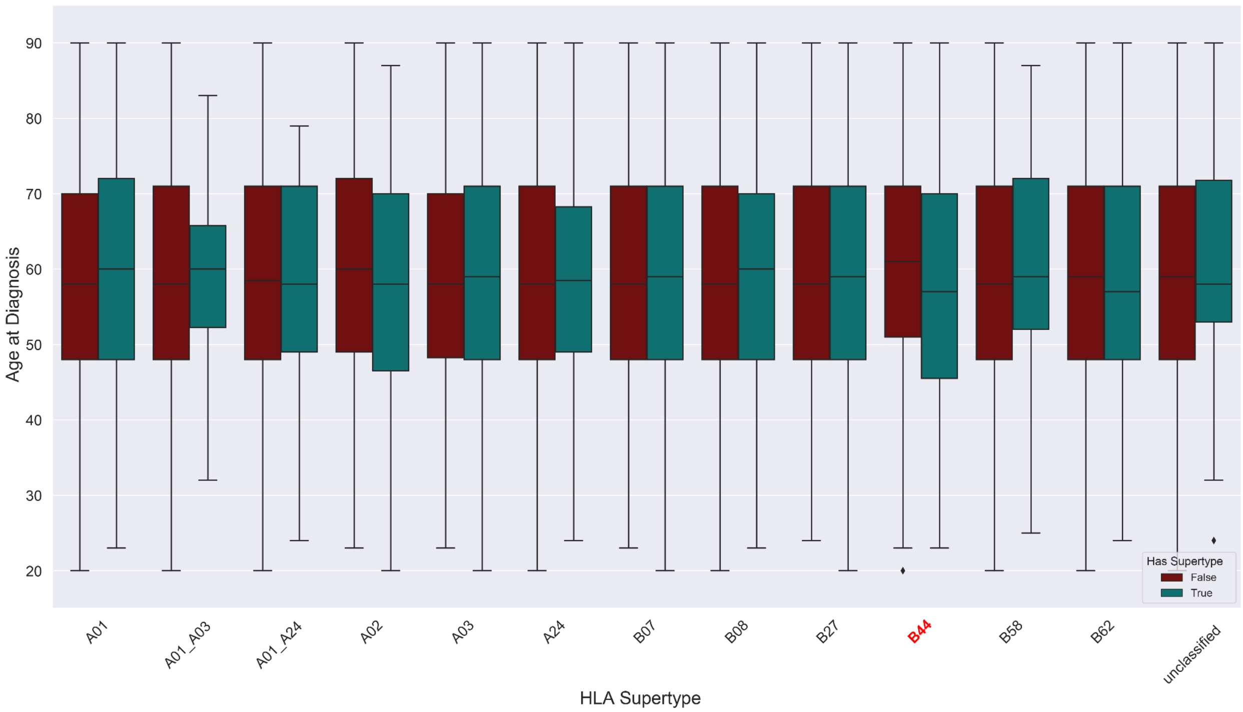

Figure S7: Effect of HLA supertype on age at diagnosis in TCGA. No significant age differences were observed across any supertype, though those with the B44 supertype (red) trended towards an earlier age of diagnosis (p = 0.199, median earlier age of diagnosis difference = 4 years).

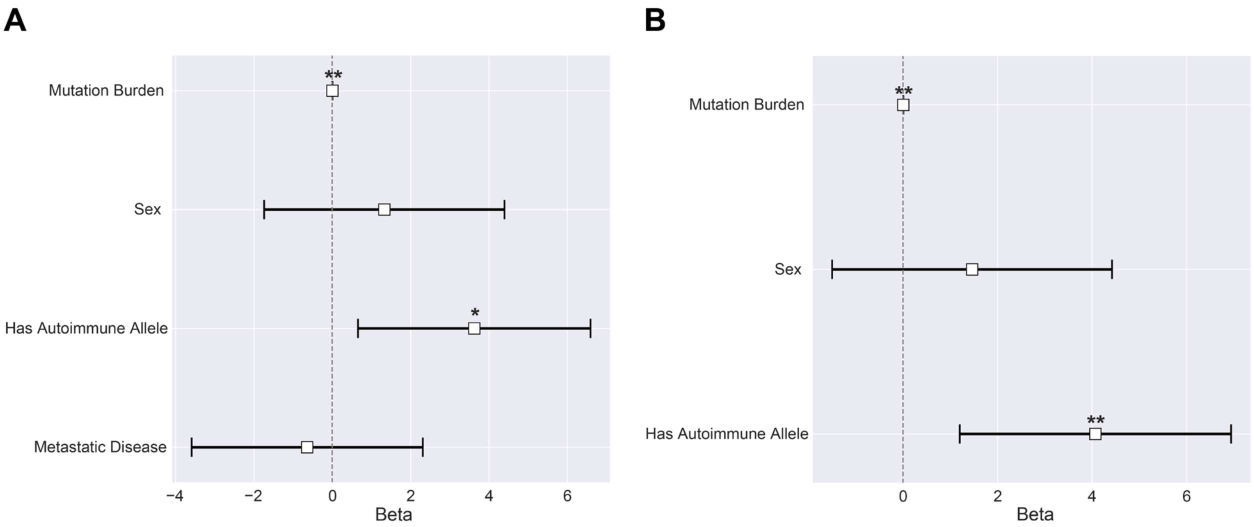

Figure S8: Regression modeling of autoimmune allele effects on melanoma age of diagnosis in TCGA. A) Having at least one MHC-I linked autoimmune allele is significantly associated with 3.62 delayed years to melanoma diagnosis after controlling for primary vs. metastatic disease, sex, and mutation burden (n = 416, $p_{autoimmune}$ = 0.015). Those with an AJCC pathologic tumor stage of II or below were assigned a primary disease label, while those with a tumor stage of III or higher were assigned a metastatic disease label. B) Having at least one MHC-I linked autoimmune allele is significantly associated with 4.07 delayed years to melanoma diagnosis after controlling for sex and mutation burden (n = 451, $p_{autoimmune}$ = 0.005).

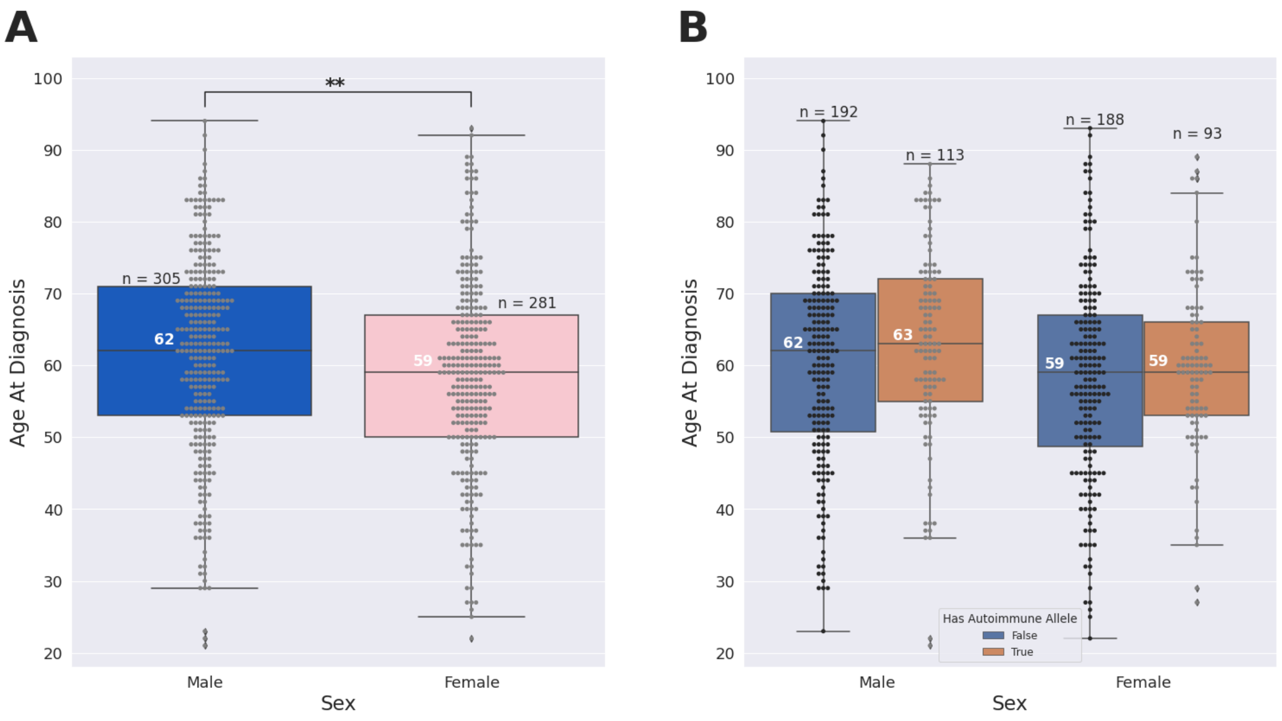

Figure S9: A) Females in the validation set showed a significant earlier age of diagnosis relative to males (p = 0.003, median difference = 3 years) B) Direction of AI carrier status effect in males is consistent with later age of diagnosis findings (p_{males} = 0.055, $p_{females}$= 0.236).

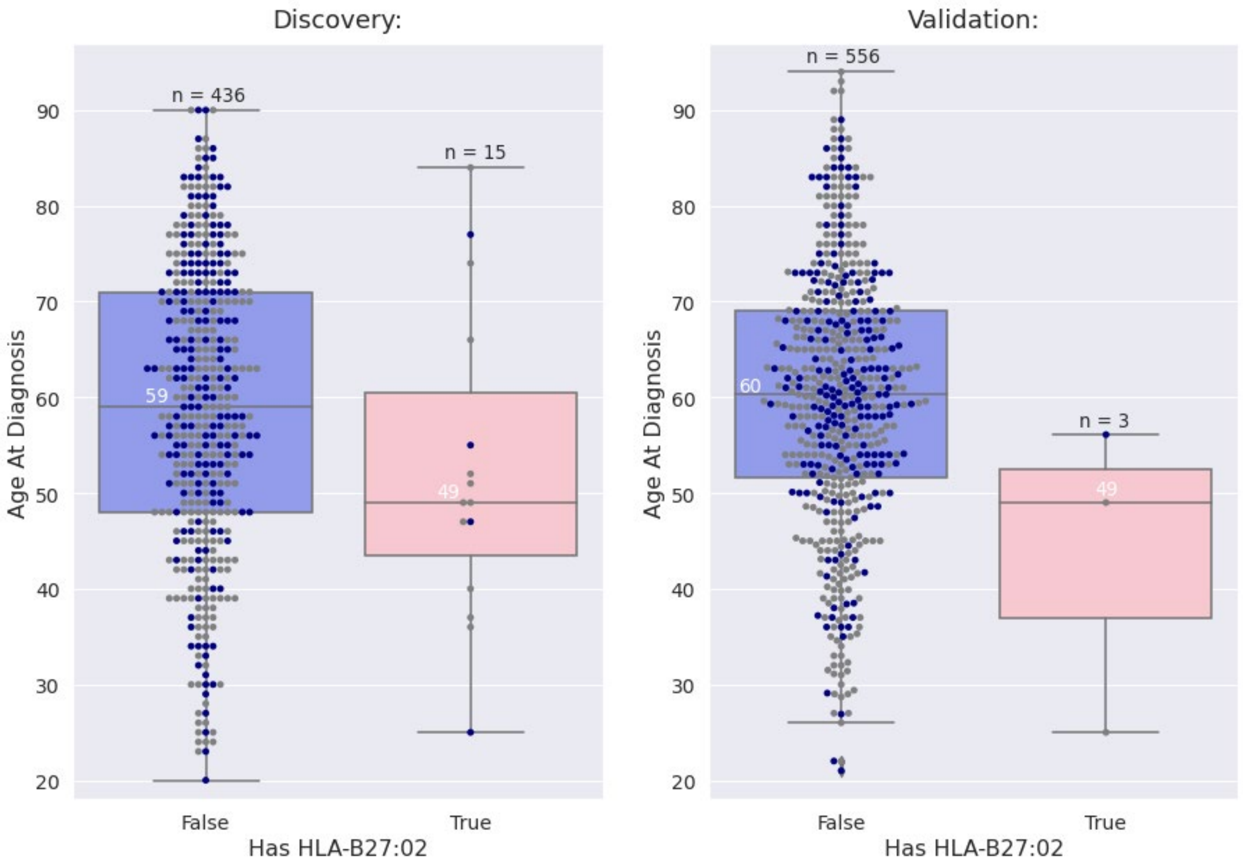

Figure S10: HLA-B*27:02 was associated with an earlier age of diagnosis across both discovery and validation sets. Blue points correspond to MHC-I autoimmune allele carriers, while gray points correspond to those without any MHC-I AI alleles.

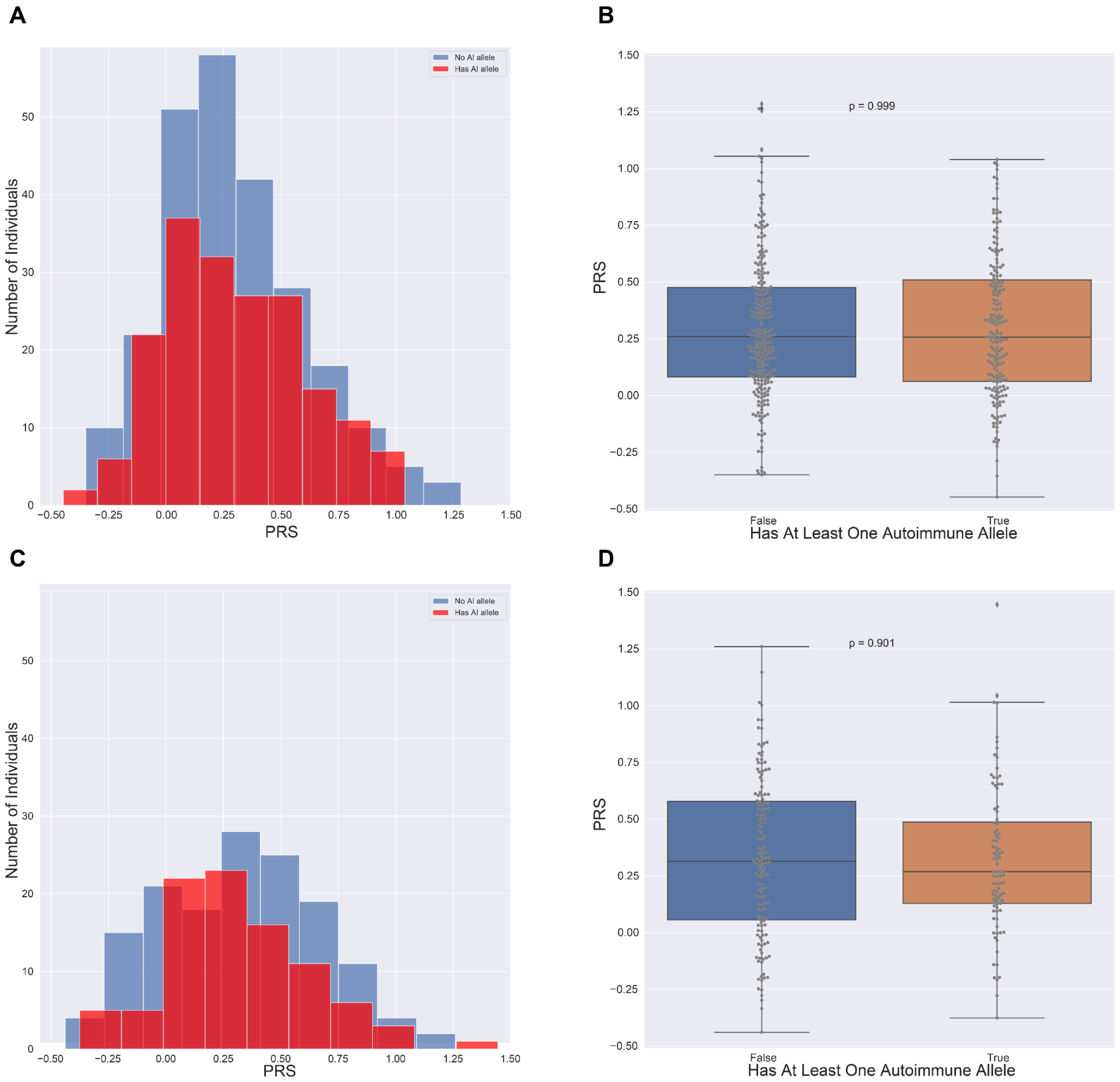

Figure S11: PRS and MHC-I autoimmune alleles exhibit independent effects with age. A) *TCGA*: Histogram of PRS stratified across individuals with and without MHC-I autoimmune alleles. B) *TCGA*: Boxplots stratifying PRS across individuals with and without MHC-I autoimmune alleles (p = 0.999, T-test). C) *Validation*: Histogram of PRS stratified across individuals with and without MHC-I autoimmune alleles. D) *Validation*: Boxplots stratifying PRS across individuals with and without MHC-I autoimmune alleles (p = 0.901, T-test).

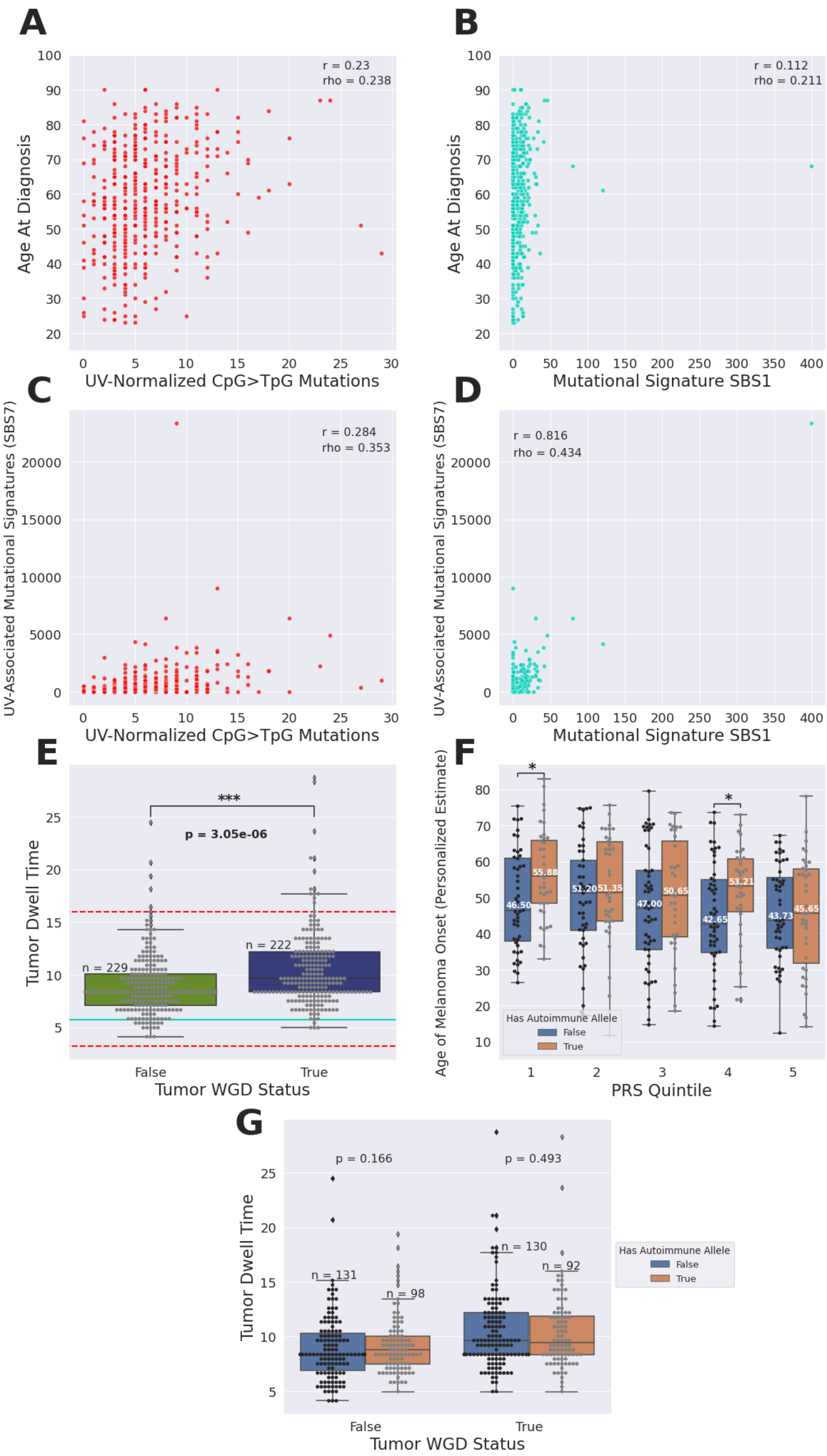

A
r = 0.23
rho = 0.238
Age At Diagnosis
UV-Normalized CpG>TpG Mutations
B
r = 0.112
rho = 0.211
Age At Diagnosis
Mutational Signature SBS1
C
r = 0.284
rho = 0.353
UV-Associated Mutational Signatures (SBS7)
UV-Normalized CpG>TpG Mutations
D
r = 0.816
rho = 0.434
UV-Associated Mutational Signatures (SBS7)
Mutational Signature SBS1
E
***
p = 3.05e-06
n = 229
n = 222
Tumor Dwell Time
False
True
Tumor WGD Status
F
*
*
55.88
46.50
51.20
51.35
47.00
50.65
42.65
53.21
43.73
45.65
Has Autoimmune Allele
False
True
Age of Melanoma Onset (Personalized Estimate)
PRS Quintile
G
p = 0.166
p = 0.493
n = 131
n = 98
n = 130
n = 92
Has Autoimmune Allele
False
True
Tumor Dwell Time
False
True
Tumor WGD Status

Figure S12: *TCGA:* A) Correlation between UV-mutation spectrum corrected CpG>TpG mutations and age of diagnosis. B) Correlation between mutational signature SBS1 and age of diagnosis. C) Correlation between UV-mutation spectrum corrected CpG>TpG mutations and UV-associated mutational signature SBS7. D) Correlation between mutational signature SBS1 and UV-associated mutational signature SBS7. E) Tumor dwell time in WGD and non-WGD melanomas. The solid blue line at 5.7 years equates to literature-reported estimated timing from WGD to melanoma diagnosis. The dotted red lines at 3.2 and 16 years represent the literature-reported WGD to melanoma diagnosis range. F) Boxplots of personalized estimated age of melanoma onset across PRS quintiles. Autoimmune MHC-I carrier status exhibited significant later predicted ages of onset for the lowest (PRS Quintile 1; $p = 0.011$) and second-highest risk quintiles (PRS Quintile 4; $p = 0.019$). G) Autoimmune MHC-I carriers do not show longer tumor dwell times, regardless of tumor WGD status.

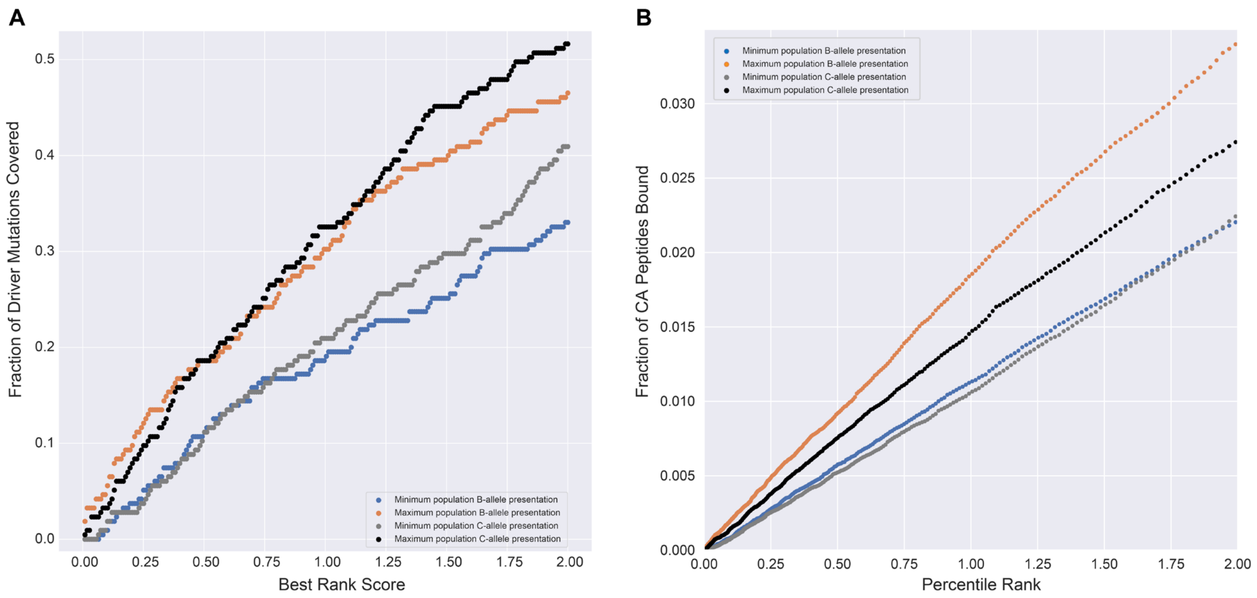

Figure S13: A) Maximum and minimum HLA-B population allele representations (Methods: *HLA Population Allele Representations*) exhibit greater neopeptide coverage than HLA-C maximum and minimum population allele representations at lower rank scores. As rank scores exceed the classical strong binding threshold (0.5), HLA-C population allele representations generally exhibit greater neopeptide coverage. B) For conserved antigens (CA), the maximum and minimum population allele representations for HLA-B presents a greater fraction of peptides than the maximum and minimum population allele representations for HLA-C. This is most pronounced in the maximum representations, where HLA-B presents a greater fraction of CA peptides compared to HLA-C as percentile rank increases.

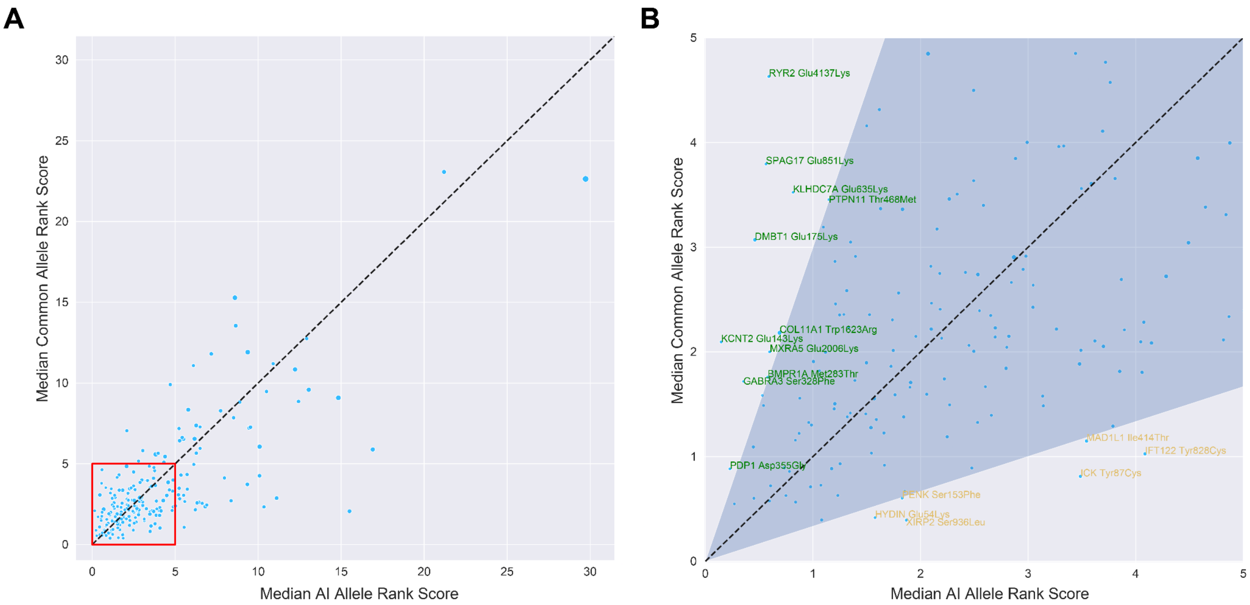

Figure S14: Predicted median driver mutation rank scores for both common and autoimmune MHC-I alleles. The varying point size reflects a mutation's standard deviation across all autoimmune alleles, with larger points having larger standard deviations. A) Predicted median rank scores for all driver mutations (n = 215) for both common and AI alleles. B) Predicted median rank scores for driver mutations in a presentable range (outlined in red in panel A). Mutations with a 3-fold median rank score discrepancy between AI and common alleles in either direction are labeled, and fall outside the shaded region. Green mutations correspond to mutations that are better presented by the median autoimmune allele, while yellow mutations correspond to mutations that are better presented by the median common allele.

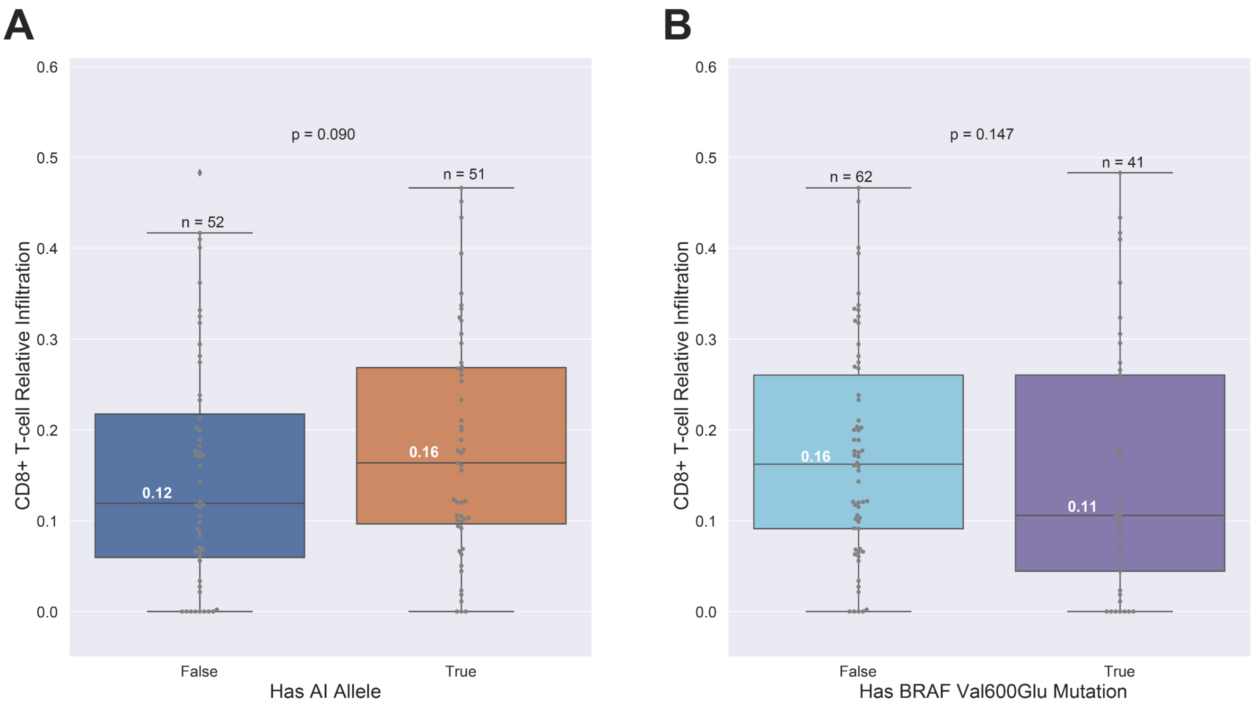

Figure S15: *TCGA*: A) CD8+ T-Cell infiltration in carriers of at least one MHC-I autoimmune allele against non-MHC-I autoimmune allele carriers in primary melanoma tumors. B) CD8+ T-Cell infiltration in individuals with a BRAF p.Val600Glu mutation against individuals without a BRAF p.Val600Glu mutation in primary melanoma tumors.

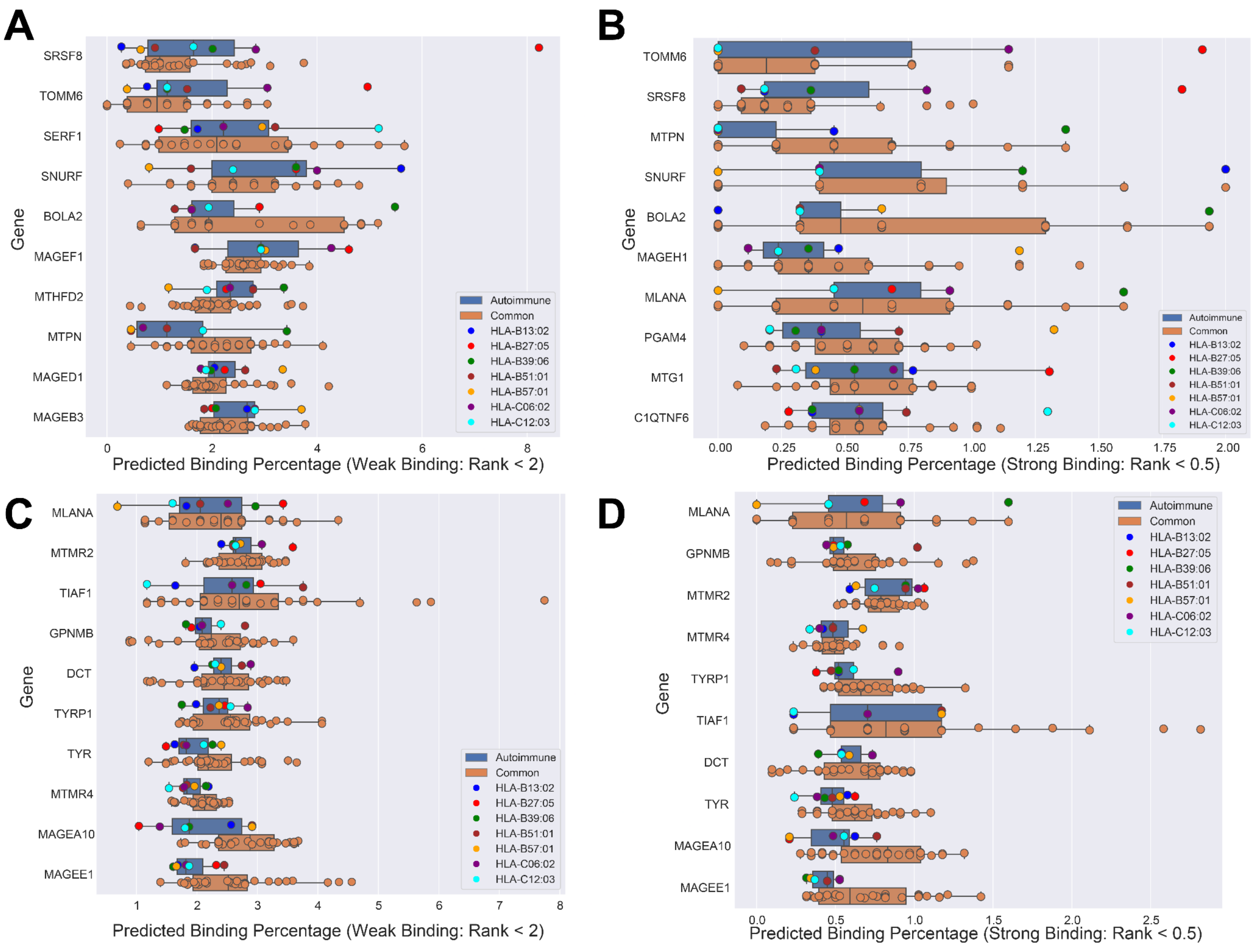

Figure S16: Distributions of the predicted binding repertoire of conserved antigens between MHC-I autoimmune and common alleles. A-B) Top 10 conserved antigens by difference in mean percentage binding (autoimmune - common). Percent binding at the A) 2% rank threshold and B) 0.5% rank threshold. C-D) Differentially expressed conserved antigens sorted as in panels A-B showing percentage binding at the C) 2% rank threshold and D) 0.5% rank threshold.

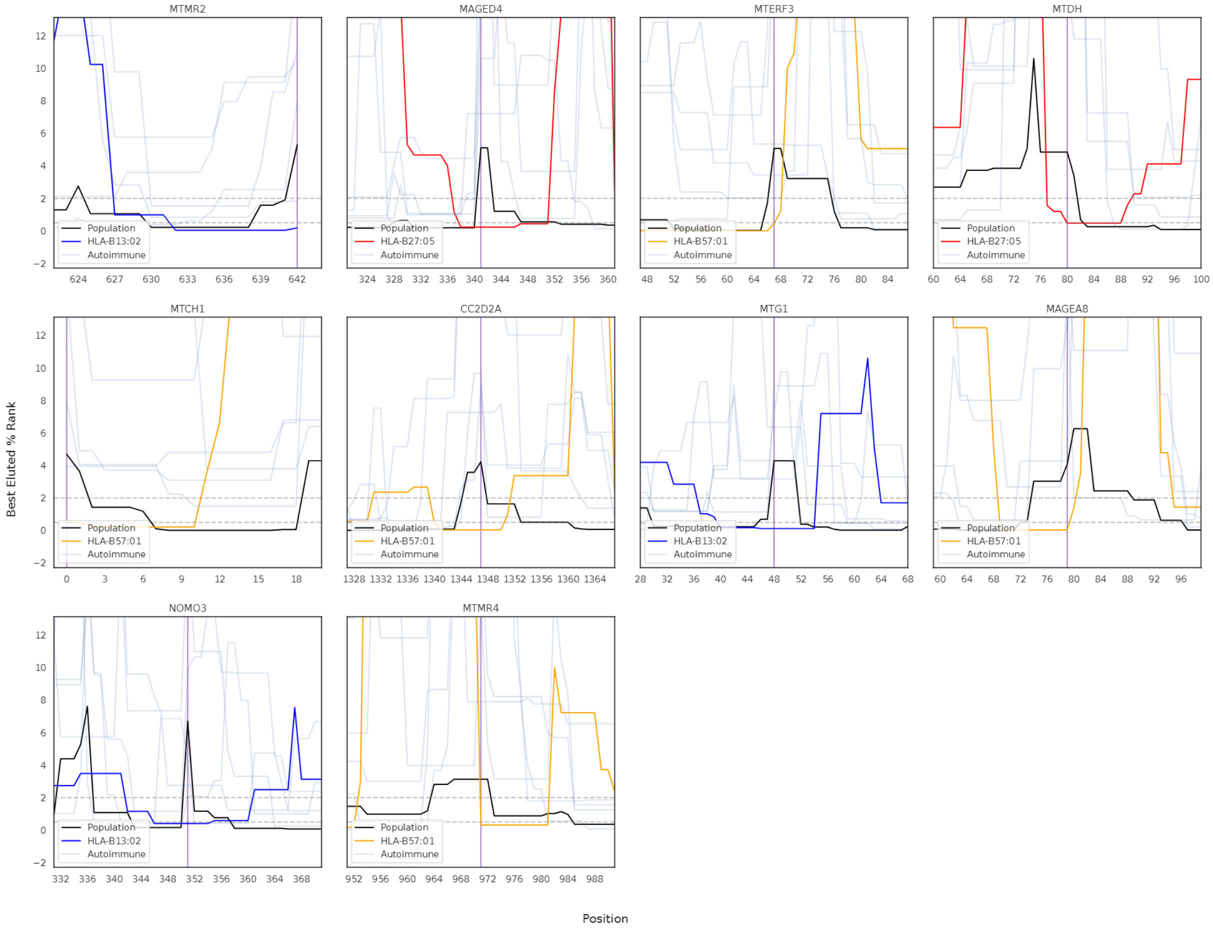

Figure S17: Greatest position-wise differences in predicted binding to conserved antigens between HLA-B autoimmune and common alleles amongst the top 8 differences (*MAGED4, MTERF3, MTDH, MTCH1, CC2D2A, MTG1, MAGEA8, NOMO3*) and DE conserved antigens (*MTMR2, MTMR4*) not shown in Figure S2B. Common alleles are shown in black and autoimmune alleles are shown in transparent blue unless they are predicted to elute at better percentile ranks than common alleles. Vertical purple lines demarcate positions where autoimmune alleles are predicted to elute at better percentile ranks than common alleles. Plots show up to +/- 20 amino acids of the demarcated positions.

	Number of Individuals	Average Age ($\pm$ σ)	Sex (M/F)
TCGA	451	58.66 $\pm$ 15.28	280/171 (37.9%)
Van Allen et al. [1]	108	59 $\pm$ 15.96	76/32 (29.6%)
Hugo et al. [2]	35	61.46 $\pm$ 12.93	24/11 (31.4%)
The Genetic and Transcriptomic Evolution of Melanoma [3]	39	55.49 $\pm$ 16.55	22/17 (43.6%)
Melanoma Exome Sequencing [4,5]	138	66.37 $\pm$ 14.01	84/54 (39.1%)
UKBB	239	56.53 $\pm$ 10.47	79/160 (66.9%)

Table S1: Study specific statistics across both the discovery (TCGA) set and validation subsets. Study statistics are reported for individuals $\geq$ 20 years old, as individuals younger than this were excluded from analyses (See Methods*: Datasets*). Values reported in parentheses correspond to the percentage of females in the particular study.

HLA-Allele	Condition(s)	Reported Odds Ratio	Reference PMID/Citation
HLA-A*02:01	Vitiligo	2.07 (1.67-2.58) [6]	17243956
		1.58 [7]	20410501
		1.52 (1.21-1.90) [8]	27689083
HLA-B*13:02	Vitiligo	1.87 (1.23-2.84) [9]	15265531
		1.88 [10]	7351272
	Psoriasis	2.77 [11]	22577363
HLA-B*27:05	Ankylosing Spondylitis	> 50 [12]	Brown, M.A. & Xu H. "Genetics of axial spondyloarthritis" in *Rheumatology*, Sixth Edition. Ed. Hochberg M.C. (2015). Available at: https://doi.org/10.1016/B978-0-323-09138-1.00116-9
	Psoriasis	3.03 (2.14, 4.28) [13]	26289052
		3.39 (2.35, 4.89) [14]	23916976
	Vitiligo	2.29 (1.30-4.06) [9]	15265531
HLA-B*39:06	Type 1 Diabetes	10.31 [15]	20798335
	Psoriasis	1.46 (1.31–1.62) [16]	25087609
HLA-B*51:01	Behcet's disease	6.04 (4.96–7.35) [17]	Koné-Paut, I. "Chapter 19 - Behçet's Disease" in *Handbook of Systemic Autoimmune Diseases*. Ed. Cimaz, R. & Lehman, T. (2016) Available at: https://www.sciencedirect.com/science/article/pii/B9780444635969000190

		5-10 [18]	34690278
	Psoriasis	N/A [19]	16335880
HLA-B*57:01	Psoriasis	3.61 [11]	22577363
HLA-C*06:02	Vitiligo	3.04 (1.79-5.18) [9]	15265531
	Psoriasis	3.57 [11]	22577363
		3.26 (3.02-3.52) [16]	25087609
		3-10 [20]	29760713
		1.54-∞ [21]	10504461
HLA-C*12:03	Psoriasis	1.38 (1.26-1.52) [16]	25087609

Table S2: Summary of MHC-I linked autoimmune alleles including reported odds ratios for the autoimmune conditions with which they are associated.

	Discovery Set (TCGA) Allele Frequencies	Validation Set Allele Frequencies	Population Allele Frequency	Autoimmune Disease Association
HLA-A*02:01	26.27% (201)	25.31% (240)	27.6%	Vitiligo [22–25]
HLA-B*13:02	2.66% (23)	2.15% (23)	2.4%	Vitiligo [9,26]
HLA-B*27:05	3.66% (33)	4.20% (42)	3.7%	Psoriasis, Ankylosing Spondylitis [11,16,27–30]
HLA-B*39:06	1.00% (9)	0.63% (7)	0.64%	Psoriasis, Type 1 Diabetes[15,16,31,32]
HLA-B*51:01	4.21% (36)	3.49% (38)	4.7%	Psoriasis, Behcet's Disease[19,33]
HLA-B*57:01	4.21% (38)	3.22% (35)	3.6%	Psoriasis [11]
HLA-C*06:02	9.42% (82)	7.60% (80)	9.3%	Vitiligo, Psoriasis [9,26,34,35]
HLA-C*12:03	5.76% (51)	4.20% (44)	4.9%	Psoriasis [16,36]

Table S3: Autoimmune HLA allele frequency in both discovery (TCGA) and validation sets compared against the population distribution as reported by the European Caucasian subset of U.S. National Marrow Donor Program (N = 1,242,890). Values reported in parentheses correspond to the number of individuals in the dataset with the respective allele.

Supplementary References:

1. Van Allen, E.M., Miao, D., Schilling, B., Shukla, S.A., Blank, C., Zimmer, L., Sucker, A., Hillen, U., Foppen, M.H.G., Goldinger, S.M., et al. (2015). Genomic correlates of response to CTLA-4 blockade in metastatic melanoma. Science *350*, 207–211.

2. Hugo, W., Zaretsky, J.M., Sun, L., Song, C., Moreno, B.H., Hu-Lieskovan, S., Berent-Maoz, B., Pang, J., Chmielowski, B., Cherry, G., et al. (2016). Genomic and Transcriptomic Features of Response to Anti-PD-1 Therapy in Metastatic Melanoma. Cell *165*, 35–44.

3. Shain, A.H., Yeh, I., Kovalyshyn, I., Sriharan, A., Talevich, E., Gagnon, A., Dummer, R., North, J., Pincus, L., Ruben, B., et al. (2015). The Genetic Evolution of Melanoma from Precursor Lesions. N. Engl. J. Med. *373*, 1926–1936.

4. Krauthammer, M., Kong, Y., Bacchiocchi, A., Evans, P., Pornputtapong, N., Wu, C., McCusker, J.P., Ma, S., Cheng, E., Straub, R., et al. (2015). Exome sequencing identifies recurrent mutations in NF1 and RASopathy genes in sun-exposed melanomas. Nat. Genet. *47*, 996–1002.

5. Lazova, R., Pornputtapong, N., Halaban, R., Bosenberg, M., Bai, Y., Chai, H., and Krauthammer, M. (2017). Spitz nevi and Spitzoid melanomas: exome sequencing and comparison with conventional melanocytic nevi and melanomas. Mod. Pathol. *30*, 640–649.

6. Liu, J.-B., Li, M., Chen, H., Zhong, S.-Q., Yang, S., Du, W.-D., Hao, J.-H., Zhang, T.-S., Zhang, X.-J., and Zeegers, M.P. (2007). Association of vitiligo with HLA-A2: a meta-analysis. J. Eur. Acad. Dermatol. Venereol. *21*, 205–213.

7. Jin, Y., Birlea, S.A., Fain, P.R., Gowan, K., Riccardi, S.L., Holland, P.J., Mailloux, C.M., Sufit, A.J.D., Hutton, S.M., Amadi-Myers, A., et al. (2010). Variant of TYR and autoimmunity susceptibility loci in generalized vitiligo. N. Engl. J. Med. *362*, 1686–1697.

8. Li, Z., Ren, J., Niu, X., Xu, Q., Wang, X., Liu, Y., and Xiao, S. (2016). Meta-Analysis of the Association between Vitiligo and Human Leukocyte Antigen-A. Biomed Res. Int. *2016*, 5412806.

9. Zhang, X.-J., Liu, H.-S., Liang, Y.-H., Sun, L.-D., Wang, J.-Y., Yang, S., Liu, J.-B., Gao, M., He, P.-P., Cui, Y., et al. (2004). Association of HLA class I alleles with vitiligo in Chinese Hans. J. Dermatol. Sci. *35*, 165–168.

10. Metzker, A., Zamir, R., Gazit, E., David, M., and Feuerman, E.J. (1980). Vitiligo and the HLA system. Dermatologica *160*, 100–105.

11. Chen, H., Hayashi, G., Lai, O.Y., Dilthey, A., Kuebler, P.J., Wong, T.V., Martin, M.P., Fernandez Vina, M.A., McVean, G., Wabl, M., et al. (2012). Psoriasis patients are enriched for genetic variants that protect against HIV-1 disease. PLoS Genet. *8*, e1002514.

12. Brown, M.A., and Xu, H. (2015). Genetics of axial spondyloarthritis. In Rheumatology, M.C. Hochberg, ed. pp. 956–959.

13. Queiro, R., Morante, I., Cabezas, I., and Acasuso, B. (2016). HLA-B27 and psoriatic disease: a modern view of an old relationship. Rheumatology *55*, 221–229.

14. Chandran, V., Bull, S.B., Pellett, F.J., Ayearst, R., Rahman, P., and Gladman, D.D. (2013). Human leukocyte antigen alleles and susceptibility to psoriatic arthritis. Hum. Immunol. *74*, 1333–1338.

15. Noble, J.A., Valdes, A.M., Varney, M.D., Carlson, J.A., Moonsamy, P., Fear, A.L., Lane, J.A., Lavant, E., Rappner, R., Louey, A., et al. (2010). HLA class I and genetic susceptibility to type 1 diabetes: results from the Type 1 Diabetes Genetics Consortium. Diabetes *59*, 2972–2979.

16. Okada, Y., Han, B., Tsoi, L.C., Stuart, P.E., Ellinghaus, E., Tejasvi, T., Chandran, V., Pellett, F., Pollock, R., Bowcock, A.M., et al. (2014). Fine mapping major histocompatibility complex associations in psoriasis and its clinical subtypes. Am. J. Hum. Genet. *95*, 162–172.

17. Koné-Paut, I. (2016). Chapter 19 - Behçet's Disease. Handbook of Systemic Autoimmune Diseases *11*, 409–426.

18. Takeno, M. (2022). The association of Behçet's syndrome with HLA-B51 as understood in 2021. Curr. Opin. Rheumatol. *34*, 4–9.

19. Yamamoto, T., Yokozeki, H., and Nishioka, K. (2005). Psoriasis arthropathy and HLA-B51: report of 5 cases. J. Dermatol. *32*, 606–610.

20. Prinz, J.C. (2018). Human Leukocyte Antigen-Class I Alleles and the Autoreactive T Cell Response in Psoriasis Pathogenesis. Front. Immunol. *9*, 954.

21. Mallon, E., Newson, R., and Bunker, C.B. (1999). HLA-Cw6 and the genetic predisposition to psoriasis: a meta-analysis of published serologic studies. J. Invest. Dermatol. *113*, 693–695.

22. Ogg, G.S., Rod Dunbar, P., Romero, P., Chen, J.L., and Cerundolo, V. (1998). High frequency of skin-homing melanocyte-specific cytotoxic T lymphocytes in autoimmune vitiligo. J. Exp. Med. *188*, 1203–1208.

23. Lang, K.S., Caroli, C.C., Muhm, A., Wernet, D., Moris, A., Schittek, B., Knauss-Scherwitz, E., Stevanovic, S., Rammensee, H.G., and Garbe, C. (2001). HLA-A2 restricted, melanocyte-specific CD8(+) T lymphocytes detected in vitiligo patients are related to disease activity and are predominantly directed against MelanA/MART1. J. Invest. Dermatol. *116*, 891–897.

24. Palermo, B., Campanelli, R., Garbelli, S., Mantovani, S., Lantelme, E., Brazzelli, V., Ardigó, M., Borroni, G., Martinetti, M., Badulli, C., et al. (2001). Specific cytotoxic T lymphocyte responses against Melan-A/MART1, tyrosinase and gp100 in vitiligo by the use of major histocompatibility complex/peptide tetramers: the role of cellular immunity in the etiopathogenesis of vitiligo. J. Invest. Dermatol. *117*, 326–332.

25. Jin, Y., Ferrara, T., Gowan, K., Holcomb, C., Rastrou, M., Erlich, H.A., Fain, P.R., and Spritz, R.A. (2012). Next-generation DNA re-sequencing identifies common variants of TYR and HLA-A that modulate the risk of generalized vitiligo via antigen presentation. J. Invest. Dermatol. *132*, 1730–1733.

26. Quan, C., Ren, Y.-Q., Xiang, L.-H., Sun, L.-D., Xu, A.-E., Gao, X.-H., Chen, H.-D., Pu, X.-M., Wu, R.-N., Liang, C.-Z., et al. (2010). Genome-wide association study for vitiligo identifies susceptibility loci at 6q27 and the MHC. Nat. Genet. *42*, 614–618.

27. Winchester, R., Minevich, G., Steshenko, V., Kirby, B., Kane, D., Greenberg, D.A., and FitzGerald, O. (2012). HLA associations reveal genetic heterogeneity in psoriatic arthritis and in the psoriasis phenotype. Arthritis Rheum. *64*, 1134–1144.

28. FitzGerald, O., Haroon, M., Giles, J.T., and Winchester, R. (2015). Concepts of pathogenesis in psoriatic arthritis: genotype determines clinical phenotype. Arthritis Res. Ther. *17*, 115.

29. Brewerton, D.A., Hart, F.D., Nicholls, A., Caffrey, M., James, D.C., and Sturrock, R.D. (1973). Ankylosing spondylitis and HL-A 27. Lancet *1*, 904–907.

30. Brewerton, D.A., Caffrey, M., Nicholls, A., Walters, D., and James, D.C. (1974). HL-A 27 and arthropathies associated with ulcerative colitis and psoriasis. Lancet *1*, 956–958.

31. Nejentsev, S., Howson, J.M.M., Walker, N.M., Szeszko, J., Field, S.F., Stevens, H.E., Reynolds, P., Hardy, M., King, E., Masters, J., et al. (2007). Localization of type 1 diabetes susceptibility to the MHC class I genes HLA-B and HLA-A. Nature *450*, 887–892.

32. Howson, J.M.M., Walker, N.M., Clayton, D., Todd, J.A., and Type 1 Diabetes Genetics Consortium (2009). Confirmation of HLA class II independent type 1 diabetes associations in the major histocompatibility complex including HLA-B and HLA-A. Diabetes Obes. Metab. *11 Suppl 1*, 31–45.

33. Ohno, S., Ohguchi, M., Hirose, S., Matsuda, H., Wakisaka, A., and Aizawa, M. (1982). Close Association of HLA-Bw51 With Behcet's Disease. Archives of Ophthalmology *100*, 1455–1458.

34. Nair, R.P., Stuart, P.E., Nistor, I., Hiremagalore, R., Chia, N.V.C., Jenisch, S., Weichenthal, M., Abecasis, G.R., Lim, H.W., Christophers, E., et al. (2006). Sequence and haplotype analysis supports HLA-C as the psoriasis susceptibility 1 gene. Am. J. Hum. Genet. *78*, 827–851.

35. Gudjonsson, J.E., Karason, A., Antonsdottir, A., Runarsdottir, E.H., Hauksson, V.B., Upmanyu, R., Gulcher, J., Stefansson, K., and Valdimarsson, H. (2003). Psoriasis patients who are homozygous for the HLA-Cw*0602 allele have a 2.5-fold increased risk of developing psoriasis compared with Cw6 heterozygotes. Br. J. Dermatol. *148*, 233–235.

36. Helms, C., Saccone, N.L., Cao, L., Daw, J.A.W., Cao, K., Hsu, T.M., Taillon-Miller, P., Duan, S., Gordon, D., Pierce, B., et al. (2005). Localization of PSORS1 to a haplotype block harboring HLA-C and distinct from corneodesmosin and HCR. Hum. Genet. *118*, 466–476.
